# Behavioral intervention to reduce opioid overdose among high-risk persons with opioid use disorder: A pilot randomized controlled trial

**DOI:** 10.1371/journal.pone.0183354

**Published:** 2017-10-19

**Authors:** Phillip Oliver Coffin, Glenn-Milo Santos, Tim Matheson, Emily Behar, Chris Rowe, Talia Rubin, Janelle Silvis, Eric Vittinghoff

**Affiliations:** 1 San Francisco Department of Public Health, San Francisco, California, United States of America; 2 University of California San Francisco, San Francisco, California, United States of America; University of New South Wales, AUSTRALIA

## Abstract

**Objective:**

The United States is amidst an opioid epidemic, including synthetic opioids that may result in rapid death, leaving minimal opportunity for bystander rescue. We pilot tested a behavioral intervention to reduce the occurrence of opioid overdose among opioid dependent persons at high-risk for subsequent overdose.

**Materials and methods:**

We conducted a single-blinded randomized-controlled trial of a repeated dose motivational interviewing intervention (REBOOT) to reduce overdose versus treatment as usual, defined as information and referrals, over 16 months at the San Francisco Department of Public Health from 2014–2016. Participants were 18–65 years of age, had opioid use disorder by Structured Clinical Interview, active opioid use, opioid overdose within 5 years, and prior receipt of naloxone kits. The intervention was administered at months 0, 4, 8, and 12, preceded by the assessment which was also administered at month 16. Dual primary outcomes were any overdose event and number of events, collected by computer-assisted personal interview, as well as any fatal overdose events per vital records.

**Results:**

A total of 78 persons were screened and 63 enrolled. Mean age was 43 years, 67% were born male, 65% White, 17% African-American, and 14% Latino. Ninety-two percent of visits and 93% of counseling sessions were completed. At baseline, 33.3% of participants had experienced an overdose in the past four months, with a similar mean number of overdoses in both arms (p = 0.95); 29% overdosed during follow-up. By intention-to-treat, participants assigned to REBOOT were less likely to experience any overdose (incidence rate ratio [IRR] 0.62 [95%CI 0.41–0.92, p = 0.019) and experienced fewer overdose events (IRR 0.46, 95%CI 0.24–0.90, p = 0.023), findings that were robust to sensitivity analyses. There were no differences between arms in days of opioid use, substance use treatment, or naloxone carriage.

**Conclusions:**

REBOOT reduced the occurrence of any opioid overdose and the number of overdoses.

**Trial registration:**

clinicaltrials.gov NCT02093559

## Introduction

The United States is amidst an opioid epidemic. In 2015, over 33,000 people died from opioid overdose in the United States, an across-the-board increase from 2014 in deaths from heroin, prescribed opioids, and other synthetic opioids.[1] Drug poisoning has been the leading cause of injury death among adults since 2009[2, 3] and overtook gun-related deaths in 2010[1]; *opioid* overdose is the leading cause of injury death among women[4] and accounts for over half of mortality among heroin users.[5] Health care utilization and costs of caring for those with problematic opioid use are substantial and increasing.[6–8] Some dependent on opioid analgesics may transition to heroin as opioid prescribing policies change,[9–14] a phenomenon that presaged a spike in heroin overdose deaths.[15] Non-fatal opioid overdose—defined as blue skin coloring, minimal respirations, or inability to be woken up after using opioids—is also a major source of morbidity.[16] Notwithstanding a nationwide effort to address this crisis, opioid overdose rates continue to climb.

Prior overdose is the strongest predictor of subsequent overdose and overdose death.[17–21] Several overdose risk factors are potentially modifiable (e.g. use of alcohol, cocaine, or benzodiazepines with opioids;[18, 22] resumption of prior use after abstinence),[18, 23, 24] yet multiple cross-sectional and longitudinal studies have found that those who have overdosed are at much higher risk of a repeat overdose (adjusted odds ratios [AORs] from 6 to 29).[17, 18, 25] The Australian Treatment Outcome Study found that 27–32% of opioid users with a prior overdose suffered a repeat overdose in any 12-month period over three years (compared to 5% of those who had not overdosed), with increasing risk after each overdose.[26] Annual mortality among those who have overdosed may be as high as 14%, more than 19 times the overall age-adjusted mortality rate in the United States.[27, 28] In San Francisco, over half of opioid users obtaining naloxone report a prior overdose.[29] Moreover, overdose is associated with seeking substance abuse treatment: 20% of persons who inject drugs (PWIDs) and overdosed in Baltimore enrolled in substance abuse treatment within 30 days of the event.[30] An intervention addressing overdose may be able to capitalize on post-overdose concerns about substance use.

While naloxone is a critical tool in responding to opioid overdose, it is insufficient alone to address the epidemic. Naloxone distribution is associated with reduced overdose death,[31–35] and co-prescription from primary care clinics has been associated with reduced opioid-related emergency department visits.[36] Nonetheless, mathematical modeling suggests that naloxone distribution alone would prevent just 6–7% of overdose deaths and may actually increase the number of non-fatal overdoses because high-risk people–those who have overdosed–remain alive.[37] Opioid overdose mortality persists, even in areas with robust naloxone programming, such as San Francisco CA and Boston MA, likely driven by a blend of access to prescription opioids, changing markets of street opioids, and social isolation among older persons.[38] Several regions of the country have also been struck by an epidemic of clandestinely-manufactured fentanyl and fentanyl analogues contaminating or replacing other street opioids, resulting in death within minutes[39] and drastically limiting the opportunity for bystander naloxone administration.

Furthermore, it is clear from prior research into behavioral interventions that, while a single dose of intervention does not have lasting effects, repeated doses may improve upon initial effectiveness.[40–46] [45, 47] [48] [49] [50] Even research on naloxone receipt, associated with increased empowerment,[51, 52] has suggested that booster sessions are needed to ensure proper use of naloxone.[51] Seeking additional strategies to augment naloxone distribution, we designed and tested a behavioral intervention to reduce opioid overdose events among high-risk individuals with prior access to lay naloxone.

## Materials and methods

To test the hypothesis that a repeated-dose brief behavioral intervention addressing opioid overdose and related risk behaviors (REBOOT) would reduce opioid overdose events, we conducted a single-blinded randomized trial of REBOOT compared to treatment as usual (TAU) among 63 persons with opioid use disorder, prior overdose, and prior receipt of take-home naloxone. As this was a pilot study also assessing feasibility and acceptability, the study was not powered for efficacy. This study took place at the San Francisco Department of Public Health and was approved by the University of California San Francisco Committee on Human Research (Study Number 13–11767).

### Recruitment

Potential participants were recruited from sites of San Francisco’s naloxone distribution program, the Drug Overdose Prevention and Education (DOPE) Project. Interested individuals were administered a questionnaire to establish preliminary eligibility and those who prescreened as eligible were scheduled for in-person screening and enrollment. At screening, participants gave informed consent and were evaluated for eligibility. Eligible participants were 18–65 years of age; were opioid dependent by Structured Clinical Interview for the DSM IV (SCID); had an opioid overdose in the preceding 5 years and had previously received take-home naloxone, both by self-report; and were able/willing to provide informed consent, communicate in English, and adhere to the visit schedule. In addition, eligible participants were positive for opioids by urine during screening, excluding opioids prescribed for agonist treatment, due to the reduced risk of overdose while engaged in agonist treatment, unless cocaine or methamphetamine were also present in the urine test. Exclusion criteria included suicidal ideation by SCID, to minimize the likelihood of intentional overdose, planning to leave the area during the study period, any medical condition that the medical director felt would likely result in death during the study period, or any other condition felt to interfere with safe participation in the study.

### Procedures

At screening, potential participants were assessed by a medical clinician for a brief medical history, opioid use disorder and suicidality by SCID, and presence of opioids by urine toxicology via MedTox EZ-Screen rapid qualitative tests (MedTox Scientific, St. Paul, MN). Eligible individuals were randomized to receive REBOOT or TAU in 2:1 block randomization. The study biostatistician generated the allocation sequence and staff not involved in the study prepared treatment allocation cards in opaque sequentially-numbered envelopes; as a participant was enrolled, the envelope that corresponded with their sequence was opened by study staff to reveal their assigned condition. Following randomization, participants who were of negative or unknown HCV or HIV serostatus received Oraquick Rapid HCV testing and/or Oraquick Advance Rapid HIV ½ testing, respectively. Those with preliminarily positive HIV results received confirmatory Stat-Pak HIV ½ testing. All participants were then administered the survey instrument by computer-assisted personal interview (CAPI), always by staff blinded to participant study arm, followed by the REBOOT or TAU intervention. Participants were seen, urine toxicology screening was completed, and follow-up CAPI and intervention were administered at months 4, 8, and 12. At month 16, CAPI was administered, urine toxicology was completed, and rapid HIV and HCV testing was done for those who were negative at baseline.

### Intervention

REBOOT was delivered by masters- and bachelors-level counselors trained by a clinical psychologist with expertise in motivational interviewing and behavioral interventions targeting substance use and HIV. Counselors delivered a 45-minute intervention based on the information-motivation-behavior skills model of behavior change.[53, 54] First, counselors reviewed opioid overdose risk factors and response, based on the Skills and Knowledge on Overdose Prevention curriculum, which has demonstrated efficacy in training drug users to properly recognize and respond to overdose.[55] Counselors then discussed personal and witnessed overdose events in detail with the participant, in an attempt to help the participant identify risk behaviors that contributed to the overdose events, such as substance use patterns or other related behaviors. Counselors then inquired as to interest in substance use disorder treatment, emphasizing the utility of medications such as methadone and buprenorphine to reduce the risk of overdose events. Counselors then assisted the participant in developing a plan to reduce the risk of future overdoses, such as waiting several hours between using opioids and other sedating drugs, engaging with a methadone program, or other strategies. Finally, counselors reviewed HIV and HCV risk behaviors and risk reduction strategies with the participant.

TAU consisted of a packet of information provided at baseline and offered at follow-up visits including information about harm reduction sites and substance use disorder treatment programs, as well as an offer to assist with referrals to any services the participant requested. We designed TAU to reflect the minimal attention dedicated to opioid overdose prevention in extant substance use services.

All REBOOT and TAU sessions were audiotaped with participant consent and >10% were randomly selected for review by a clinical psychologist to ensure intervention fidelity and facilitate counselor feedback, if appropriate. Each required activity for both the REBOOT (e.g., review participants overdose experience and risk behaviors, provide information regarding overdose risks and prevention) and TAU (e.g., provide resource packet or inquire whether participant wants or needs referrals) groups were rated on a 4-point scale for completion: 0 (not at all); 1 (somewhat); 2 (mostly); and 3 (completely). Consistent with prior behavioral trials,[56, 57] median ratings between 1.5 and 2.5 were classified as good and greater than or equal to 2.5 were classified as excellent.

### Measures

Behavioral assessments via CAPI were completed by participants at enrollment and follow-up visits every 4 months (4, 8, 12 and 16 month visits), with questions on overdose events, substance use, substance use treatment, dependence and HIV-related risk behaviors.[58–60] Reporting periods were 120 days at baseline and the time between consecutive study visits at follow-up visits. The primary outcome was number of overdose events, determined by self-report through a standard question structure that has proven reliable in multiple settings.[18, 24] After defining opioid overdose as minimal to no breathing or not being able to wake up without assistance, participants were asked how many overdoses they had experienced during the reporting period, then asked detailed questions regarding the most recent overdose events (see S1 File for study quetionnaire).

### Data analysis

Primary outcome data were analyzed by intention-to-treat, without regard to adherence, using generalized estimating equations (GEE) Poisson models for the number of OD events in each reporting period, with robust standard errors to account for within-subject correlation and over-dispersion. We also used GEE Poisson models[61] to estimate treatment effects on risk of any OD events in each reporting period, again with robust standard errors, in this case to accommodate the binary outcome. To obtain direct estimates of risk ratios (RRs), log-link models were used. The analysis compared trends in overdose events from baseline through month 16 modeled as group-specific linear functions of time since randomization (visits coded in 4 month-scale). In both models, the linear effect of treatment was captured by the divergence of REBOOT and TAU trends at month 16, net of the fitted baseline difference, and was assessed using a test for the time-by-treatment interaction. Use of robust standard errors allowed us to account for within-subject correlation of the responses without making parametric assumptions. Fit of the model was informally assessed by plotting the group-specific fitted trends along with observed values. Sensitivity analyses were conducted: 1) excluding participants who did not report injection drug use; 2) imputing an overdose event for all missing data (for the log-link model only); 3) sequentially adjusting for imbalanced baseline characteristics (evaluated using Fisher’s exact and Wilcoxon rank-sums tests for categorical and continuous variables, respectively) and baseline correlates of overdose events; 4) trimming outliers for overdose events to the 99^th^ percentile value of overdose events; 5) assuming a constant treatment effect. All available data were included in each analysis.

We used linear, logistic and negative binomial GEE models to assess treatment effects on secondary outcomes, including overdose risk behaviors such as concurrent alcohol and substance use with opioids; HIV-related sexual (i.e., sexual intercourse with partners with discordant or unknown HIV status) and injection risk behaviors (i.e., shared needles, shared a cooker, or reported backloading and two composite variables, one for any sharing of needles or cooker, and one for any sharing of needles or cooker or backloading); opioid use and type (based on qualitative urine screening tests); days of no opioid use; number of days on which participants were involved with any drug or alcohol treatment; whether or not the participant reported receiving any drug or alcohol treatment; whether or not the participant carried naloxone; and whether or not the participant administered naloxone during witnessed overdose events. Acceptability was evaluated as percent of visit and counseling sessions completed.

## Results

### Subjects

**Fig 1:**
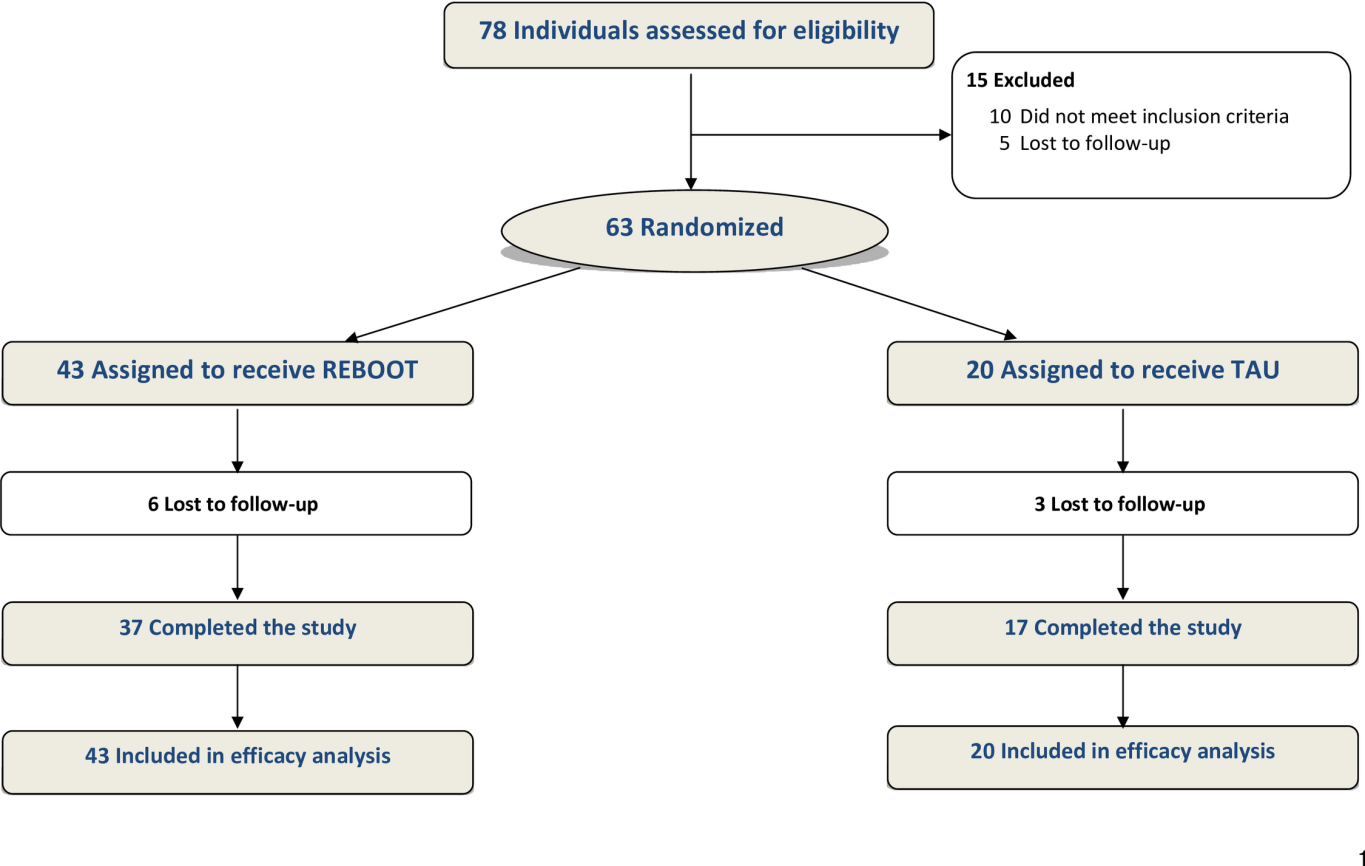
Participant flow diagram for REBOOT study, San Francisco 2014–2016.

Following an initial pilot among 7 individuals (4 injected heroin, 3 used illicit opioid analgesics) in which the intervention was found to be acceptable, engaging, and of immediate relevance to their health concerns, 63 participants were enrolled (43 REBOOT, 20 TAU; 60 PWID, 3 non-injectors [all randomized to REBOOT]). Data were collected from August 2014 to December 2016. Fig 1 shows results for screening, study arm assignment and retention; 78 participants provided written informed consent for screening. The reasons for ineligibility included (some participants had more than one reason): no positive urine result for opioids during screening (n = 7); only using prescribed opioids (n = 3); having a medical condition that precluded safe study participation, based on clinical judgement (n = 1); no overdose event in the past 5 years (n = 1); not opioid dependent (n = 1). Five participants were eligible but were lost to follow-up between their screening and enrolment visits.

Participant mean age was 43.3 years (SD 11.7), 67% were born male, 65% white, 17% African-American, and 14% Latino. Participant characteristics were similar in both arms (Table 1).

**Table 1:** Baseline characteristics of REBOOT participants, by treatment arm, San Francisco 2014–2016.

		Control (N = 20)		REBOOT (N = 43)		Overall (N = 63)		p
		N	%	N	%	N	%	
*Age*								
* *	*mean (SD)*, *years*	42.6	(12.5)	43.6	(11.4)	43.3	(11.70)	0.78
*Gender*								
* *	*Female*	10	(50.0)	11	(25.6)	21	(33.30)	0.09
* *	*Male*	10	(50.0)	32	(74.4)	42	(66.70)	
*Race*								
* *	*African American*	4	(20.0)	7	(16.3)	11	(17.50)	0.32
* *	*White*, *Caucasian*	12	(60.0)	29	(67.4)	41	(65.10)	
* *	*Other*	4	(20.0)	7	(16.3)	11	(17.50)	
*Ethnicity*								0.25
* *	*Non-Latino/Non-Hispanic*	19	(95.0)	35	(81.4)	54	(85.70)	
* *	*Latino/Hispanic*	1	(5.0)	8	(18.6)	9	(14.30)	
*HIV Status*								>0.99
* *	*Negative*	19	(95.0)	39	(90.7)	58	(92.10)	
* *	*Positive*	1	(5.0)	4	(9.3)	5	(7.90)	
*HCV Status*								0.33
* *	*Negative*	4	(20.0)	7	(16.3)	11	(17.50)	
* *	*Positive*	14	(70.0)	35	(81.4)	49	(77.80)	
* *	*Unknown*	1	(5.0)	0	0.0	1	(1.60)	
*Education*								0.95
* *	*Less than high school*	6	(30.0)	10	(23.3)	16	(25.40)	
* *	*High school graduate*	6	(30.0)	15	(34.9)	21	(33.30)	
* *	*Some college*, *2-year*	7	(35.0)	15	(34.9)	22	(34.90)	
* *	*Bachelor's degree*	1	(5.0)	3	(7.0)	4	(6.30)	
*Income*								0.35
* *	*No income*	6	(30.0)	14	(32.6)	20	(31.70)	
* *	*$1 to $9999*	4	(20.0)	12	(27.9)	16	(25.40)	
* *	*$10*,*000 to 19*,*999*	5	(25.0)	13	(30.2)	18	(28.60)	
* *	*$20*,*000 to 29*,*999*	2	(10.0)	1	(2.3)	3	(4.80)	
* *	*$30*,*000+*	3	(15.0)	3	(7.0)	6	(9.50)	
*Health Insurance*								0.08
* *	*No*	4	(20.0)	2	(4.7)	6	(9.50)	
* *	*Yes*	16	(80.0)	41	(95.3)	57	(90.50)	
*Health Provider*								0.78
* *	*No*	7	(35.0)	18	(41.9)	25	(39.70)	
* *	*Yes*	13	(65.0)	25	(58.1)	38	(60.30)	
*History homelessness*								>0.99
* *	*No*	1	(5.0)	2	(4.7)	3	(4.80)	
* *	*Yes*	19	(95.0)	41	(95.3)	60	(95.20)	
*Ever received drug use treatment*								>0.99
	No	2	(10.0)	6	(14.0)	8	(12.70)	
	Yes	18	(90.0)	37	(86.0)	55	(87.30)	
*Participated in methadone treatment program in past 4 months*								0.14
	No	17	(85.0)	28	(65.1)	45	(71.4)	
	Yes	3	(15.0)	15	(34.9)	18	(28.6)	
*Participated in buprenorphine treatment program in past 4 months*								0.54
	No	19	(95.0)	42	(97.7)	61	(96.8)	
	Yes	1	(5.0)	1	(2.3)	2	(3.2)	
*Opiates Used in the past 4 months*								
	*Heroin*	20	(100.0)	42	(97.7)	62	(98.4)	>0.99
	*Oxymorphone*	4	(20.0)	7	(16.3)	11	(17.5)	0.732
	*Morphine*	13	(65.0)	28	(65.1)	41	(65.1)	>0.99
	*Hydrocodone*	6	(30.0)	11	(25.6)	17	(27.0)	0.765
	*Fentanyl*	10	(50.0)	11	(25.6)	21	(33.3)	0.085
	*Hydromorphone*	7	(35.0)	13	(30.2)	20	(31.7)	0.775
	*Oxycodone*	4	(20.0)	9	(20.9)	13	(20.6)	>0.99
	*Methadone*	8	(40.0)	19	(44.2)	27	(42.9)	0.791
	*Buprenorphine*	3	(15.0)	3	(7.0)	6	(9.5)	0.372
	*Propoxyphene*	1	(5.0)	0	0.0	1	(1.6)	0.317
	*Meperidine*	0	0.0	2	(4.7)	2	(3.2)	>0.99
	*Codeine*	5	(25.0)	7	(16.3)	12	(19.0)	0.496
*Other substances Used in past 4 months*								
	*Alcohol*	10	(50.0)	26	(60.5)	36	(57.1)	0.585
	*Benzodiazepines*	9	(45.0)	23	(53.5)	32	(50.8)	0.595
	*Crack Cocaine*	11	(55.0)	22	(51.2)	33	(52.4)	0.794
	*Powder Cocaine*	8	(40.0)	18	(41.9)	26	(41.3)	>0.99
	*Methamphetamine*	11	(55.0)	25	(58.1)	36	(57.1)	>0.99
	*Other tranquilizers/barbiturates*	0	0.0	1	(2.3)	1	(1.6)	>0.99
*Opioid used concurrent with substances below in past 4 months*								
	*Alcohol*	8	(40.0)	25	(58.1)	33	(52.4)	0.278
	*Benzodiazepines*	8	(40.0)	22	(51.2)	30	(47.6)	0.432
	*Crack Cocaine*	11	(55.0)	22	(51.2)	33	(52.4)	0.794
	*Powder Cocaine*	8	(40.0)	16	(37.2)	24	(38.1)	>0.99
	*Methamphetamine*	11	(55.0)	24	(55.8)	35	(55.6)	>0.99

### Study participation, acceptability, and fidelity

Ninety-two percent of 315 possible visits were completed, with 93% of 172 possible counselling sessions completed among the intervention participants (90% if half-completed sessions are excluded). The majority of participants reported that they would definitely recommend the study to peers (87%), were very satisfied with the amount of help they received from being part of the study (87%), and would definitely come back to participate in a future study (81%). In regards to intervention fidelity, all assessed REBOOT counselling (n = 19) and TAU sessions (n = 36) were classified as excellent.

### Overdose event outcomes

**Fig 2:**
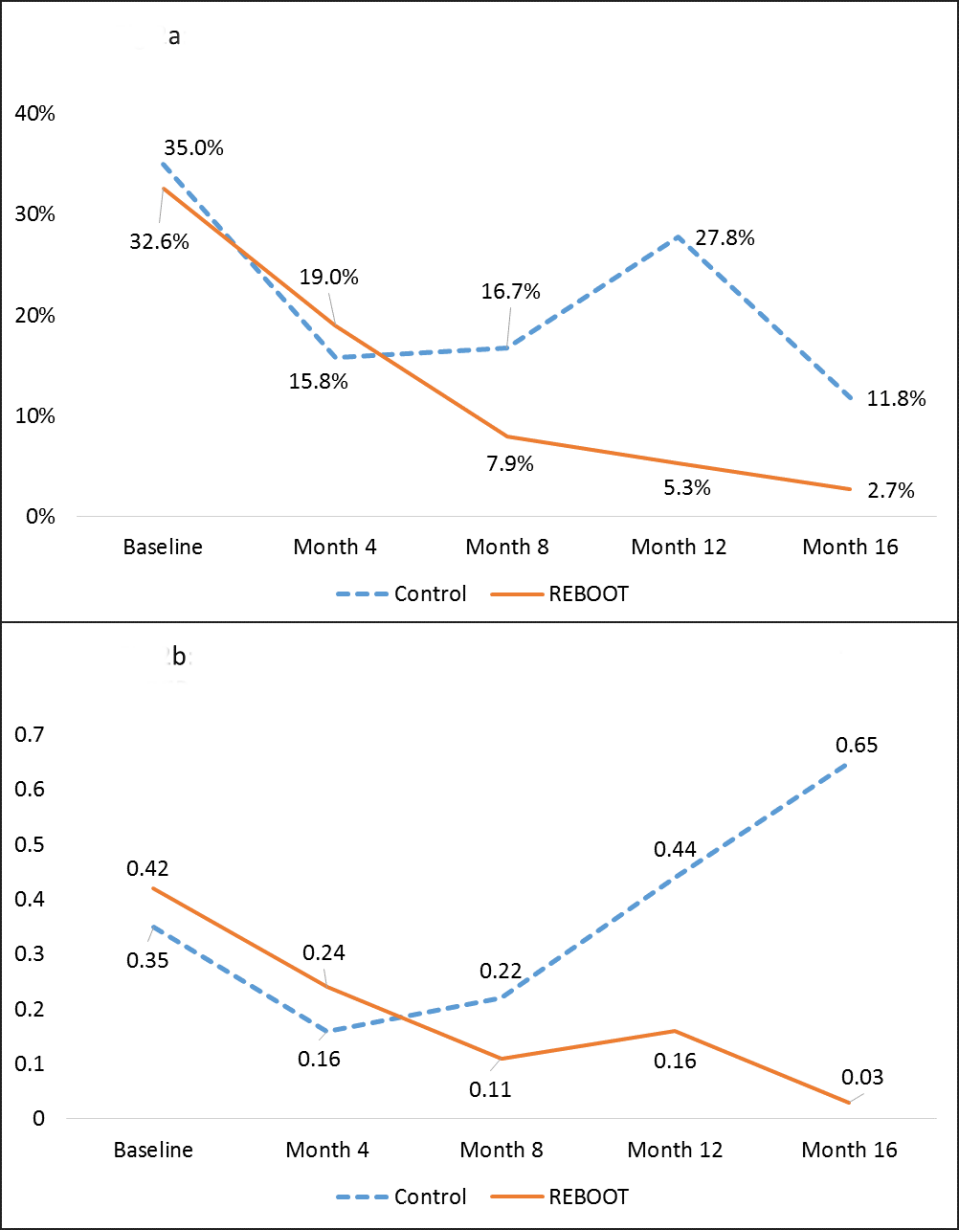
(a) Proportion of REBOOT and control participants with any overdose in preceding 4 months, San Francisco 2014–2016; (b) Mean number of overdoses in preceding 4 months per REBOOT and control participant, San Francisco 2014–2016.

At baseline, 49.2% of participants had suffered an opioid overdose in the past 12 months and 33.3% had overdosed in the past 4 months. During 16 months of follow-up, 29% of participants experienced an overdose. There were no fatal overdose events. The mean number of overdose events in the past 4 months reported at baseline was similar for both arms (REBOOT = 0.42 [SD = 0.66] vs. TAU = 0.35 [SD = 0.49]; p = 0.95). At month-16 follow-up visits, the mean number of overdose events decreased significantly among REBOOT participants, compared to TAU (see Fig 2A and 2B).

The results of the intention-to-treat GEE analyses on overdose outcomes are shown in Table 2. The incidence rate ratio (IRR) for number of overdose events among intervention compared to TAU participants assuming a linear treatment effect was 0.46 (95%CI 0.24–0.9, p = 0.023). There was no evidence of departure from linearity (p = 0.31). This protective effect in favour of REBOOT on number of overdose events remained statistically significant in sensitivity analyses after excluding non-injecting participants (p = 0.023); adjusting for baseline correlates of any overdose events (p<0.001); and trimming outliers of number of overdose events (p = 0.023). In a sensitivity analysis assuming a constant treatment effect instead of a linear treatment effect, we also observed an IRR associated with a protective treatment effect (IRR = 0.32, 95%CI 0.11–0.93, p = 0.036).

**Table 2:** Opioid overdose outcomes and sensitivity analyses among REBOOT compared to control participants, San Francisco 2014–2016.

*Outcome*	*Model*	*IRR*	*95% CI*			*p-value*
**Number of Overdose Events**	Linear treatment effect	0.46	0.24	-	0.90	0.023
	*Sensitivity analyses*:					
	Linear treatment effect, excluding non-injectors	0.47	0.24	-	0.91	0.026
	Linear treatment effect, adjusting for baseline correlates of number of overdose events^α^	0.47	0.34	-	0.65	<0.001
	Linear treatment effect, trimming outliers for number of overdose events^†^	0.54	0.31	-	0.92	0.023
	Linear treatment effect, adjusting for baseline imbalances	(no baseline imbalances between arms)				
	Constant treatment effect	0.32	0.11	-	0.93	0.04
**Any Overdose Events**	Linear treatment effect	0.62	0.41	-	0.92	0.019
	*Sensitivity analyses*:					
	Linear treatment effect, excluding non-injectors	0.64	0.43	-	0.94	0.024
	Linear treatment effect, adjusting for baseline correlates of any overdose^α^	0.58	0.41	-	0.81	0.001
	Linear treatment effect, adjusting for baseline imbalances	(no baseline imbalances between arms)				
	Linear treatment effect, singularly imputing missing outcomes as any overdose	0.62	0.41	-	0.92	0.019
	Constant treatment effect	0.54	0.21	-	1.42	0.21

^† ^One observation in the TAU control group reported 10 overdose events, which was trimmed to 5 (the 99^th^ percentile value for number of overdose events).

^α ^Baseline demographic, social and behavioral characteristics fitted in GEE models for number of overdose events and any overdose events to screen for correlates, using a p-value cut-off of <0.05. Adjusted baseline correlates included: oxymorphone use, alcohol use, alcohol use concurrent with opiates, and methamphetamine use concurrent with opiates.

The GEE analyses assuming a linear treatment effect for any overdose event also showed significant reductions among REBOOT participants, compared to TAU (IRR = 0.62; 95%CI 0.41–0.92, p = 0.019). There was no evidence of departure from linearity (p = 0.62). This protective effect in favour of REBOOT on any overdose events remained statistically significant in sensitivity analyses after excluding non-injecting participants (p = 0.024); and adjusting for baseline correlates of any overdose events (p = 0.001). In a sensitivity analysis singly imputing missing outcomes as an overdose event, the IRR was also associated with a protective effect in favour of the intervention arm (p = 0.019). In a sensitivity analysis assuming a constant treatment effect instead of a linear treatment effect, we observed an IRR suggestive of a protective effect (IRR = 0.54, 95%CI 0.21–1.42) that was not statistically significant (p = 0.212).

### Secondary outcomes

There were no statistically significant baseline differences between arms for the secondary outcomes on concurrent alcohol and substance use with opioids; HIV-related sexual and injection risk behaviors; opioid use based on qualitative urine test results; days of no opioid use; number of days on which participants were involved with any drug or alcohol treatment; whether or not the participant reported receiving any drug or alcohol treatment; whether or not the participant carried naloxone; and whether or not the participant administered naloxone during witnessed overdose events (all p>0.05). In addition, there were no statistically significant treatment effects on these secondary outcomes during follow-up when we compared REBOOT to TAU (all p>0.05).

### Adverse events

There were no study-related adverse events. Review of vital records at the conclusion of the study identified one death, a participant who died from endocarditis during follow-up.

## Discussion

REBOOT enrolled with a low screening to enrollment ratio, maintained remarkably high visit completion for a study of such duration in a substance-using population, and was well-received by subjects. Most notably, the outcomes of any opioid overdose event and the number of events were both significantly reduced among REBOOT participants compared to controls. These data support the implementation of REBOOT in a full, formal efficacy trial.

There are no proven behavioral interventions to reduce the risk of an overdose event occurring among people at high risk of opioid overdose. While some data suggest that providing naloxone in a primary care setting may reduce opioid-related emergency department visits, that study was not powered to detect an effect on opioid overdose events and was limited to primary care patients.[36] Moreover, studies of naloxone distribution to illicit opioid users have failed to demonstrate a reduction in overdose events[31] and mathematical modeling suggests that naloxone may increase overdose events by helping to sustain those opioid users at highest risk for future overdose.[62] Given the ongoing epidemic of opioid overdose, interventions that reduce the risk of an overdose event, not just the likelihood of subsequent death, are needed.

The results of this intervention study are particularly relevant as we attempt to respond to the epidemic of clandestinely-manufactured synthetic opioids that has swept the eastern U.S. and western Canada.[38] Given the rapidity with which use of fentanyl or its analogues results in overdose and death,[38, 63] there may be insufficient time to respond to overdose and a more urgent need to prevent its occurrence in the first place. While modifications may be needed to adapt this intervention to use of fentanyl and analogs, these results show great promise.

Drug overdose has yet to be systematically addressed in substance use disorder services. Although the Substance Use and Mental Health Services Administration has recommended providing naloxone to patients leaving detoxification services since 2015,[64] few programs provide such service. Moreover, there are no data collection requirements or standards regarding overdose in substance use treatment settings, even though death from such events is a frequent cause of death when clients are discharged.[65] Efficacy of this intervention in a full trial would support incorporating discussion of opioid overdose into multiple clinical interactions with persons suffering from opioid use disorder.

Our study has some limitations. First, this was a pilot study and results are subject to influence from a few participants. It is possible that a full trial could fail to show positive results. Second, the primary outcome of overdose events was collected by participant self-report and thus subject to both social desirability and recall bias. To mitigate the possibility of social desirability-related underreporting of overdose events and risk behaviors among participants, CAPI interviews were conducted prior to counseling sessions and by study staff who had no other interaction with the participants. In addition, we have no reason to suspect differential recall of overdose events between treatment arms. Third, study results may not be generalizable beyond San Francisco, a city with robust services including not only naloxone distribution, but also agonist treatment for opioid use disorder available within 48 hours of request. Finally, a 45-minute intervention focused on overdose may be challenging to implement, thus effectiveness may differ from efficacy.

## Conclusion

In summary, a pilot study of REBOOT demonstrated ready recruitment, high retention, and significant reductions in occurrence of any overdose and the number of overdose events among intervention participants compared to treatment as usual.

## Supporting information

S1 File REBOOT questionnaire. (PDF)

S2 File Trial protocol. (DOCX)

S3 File CONSORT checklist. (PDF)
