# Supplementary material for: Behavioral intervention to reduce opioid overdose among high-risk persons with opioid use disorder: A pilot randomized controlled trial
Source: PLoS One. 2017 Oct 19;12(10):e0183354. doi: 10.1371/journal.pone.0183354 (PMC5648110; doi:10.1371/journal.pone.0183354)
Supplement: S1 File — (PDF) [file pone.0183354.s001.pdf]

Supplemental Information File 1:

REBOOT Information Sheet and Questionnaire

[Information sheet reviewed with participants and available throughout administration of questionnaire]

HELPFUL DEFINITIONS

OPIATE

Opiates are either **heroin** or **any prescription opiates**, including:

- Oxymorphone (Opana)
- Morphine (MSContin, Kadian, Embeda, Avinza)
- Hydrocodone (Vicodin, Lorcet, Lortab, Norco, Zohydro)
- Fentanyl (Duragesic)
- Hydromorphone (Dilaudid, Palladone)
- Oxycodone (Percocet, OxyContin, Roxicodone, Percodan)
- Methadone (Dolophine)
- Buprenorphine (Suboxone, Subutex)
- Propoxyphene (Darvocet)
- Meperidine (Demerol)
- Codeine (Tylenor with Codeine, TyCo, Tylenol #3)

OPIATE OVERDOSE

Opiate overdose means someone takes opiates and then:

- The person is unresponsive when shaken or their name is called
- The person CAN'T be woken up without help (for example CPR or Naloxone)
- The person's skin, lips, or fingers turn blue
- The person stops breathing, or breathes really slowly

NALOXONE,

Naloxone is also called **NARCAN**. It is a medication that reverses an opiate overdose. It can be injected or sprayed up someone's nose.

If you have questions about any terms in the survey, please ask staff for help.

REBOOT QUESTIONNAIRE

Q1a. Participant I.D.

__ __ __ __

Q1b. Confirm Participant I.D.

__ __ __ __

If Q1a is not equal to Q1b then Participant ID numbers did not match. Please re-enter. and skip to Q1a.

Q2. What is your date of birth? mm / dd / yyyy

__ __ / __ __ / __ __ __ __

Q3a. Visit type (Choose one)

1 Baseline visit
2 Week 4
3 Week 8
4 PT week 12
5 PT week 36
5 Early Termination

Q4b. ARE YOU SURE? Confirm visit type. (Choose one)

1 Baseline visit
2 Week 4
3 Week 8
4 PT week 12
5 PT week 36
5 Early Termination

If Q3a is not equal to Q4b then Visit type was not confirmed. Please re-enter. and skip to Q3a.
If Q3a is equal to 1, then skip to instruction before Q7.

Q5. Enter the date of the participant's last ACASI. mm / dd / yyyy

__ __ / __ __ / __ __ __ __

Q6. Re-enter the date of the participant's last ACASI. mm / dd / yyyy

__ __ / __ __ / __ __ __ __

If Q5 is not equal to Q6 then Please enter matching visit dates. and skip to Q5.

<u>This is the TASP-C ACASI! Please quit if you did not mean to launch TASP-C.</u>

Q7. Staff ID code

__ __ __

Q8. CAPI Administrator ID code

__ __ __

Q9. Computer number

__

We will now begin the questionnaire.

Demographics

If Q3a is greater than 1, then skip to A15.

The first several questions will ask some general things about you, such as your age and income level. Please answer each question as carefully as you can. Remember, there are no right or wrong answers. Also, your counselor and clinician will NOT have access to your responses to these questions.

A1. What is your Gender? (Choose one)
0 Female
1 Male
2 Transfemale (Transgender Male to Female)
3 Transmale (Transgender Female to Male)

A2. What is your Ethnicity? (Choose one)
0 Non-Latino/Non-Hispanic
1 Latino/Hispanic
8 Refuse to Answer

A3. What is your primary race? (Choose one)
1 African American or Black
2 Asian American or Pacific Islander
3 Native American, American Indian, or Alaskan Native
4 White, Caucasian, or European American
5 Mixed or Multi-racial
6 Other
8 Refuse to Answer

If A3 is not equal to 5, then skip to instruction before A3b.

A3a. Mixed race: please specify

__ __

If A3 is not equal to 6, then skip to A4.

A3b. Other race: please specify

__ __

A4. What is your date of birth? __ __ / __ __ / __ __ __ __ mm / dd / yyyy

If AGE is less than 18 or AGE is greater than 65 then You reported you are under 18 or over 65. Please confirm your date of birth. If you are under 18 or over 65, please contact a study staff person. and skip to A3b.

A5. Were you born in the U.S.?
1 Yes
0 No
8 Refuse to Answer

If A5 is equal to 1, then skip to A7.

A6. Please specify your country of birth:

__ __

A7. What is the highest level of education you have completed? (Choose one)
1 Less than high school graduate
2 High school graduate or GED
3Some college, 2-year college degree, or Associate's Degree
4 Bachelor's degree
5 Master's degree or higher
8 Refuse to Answer

A8. Are you currently enrolled in school? (Choose one)
0 No
1 Yes, full-time
2 Yes, part-time

A9. Please identify your current employment status? (Choose one)
1 Not employed
2 Employed full-time
3 Employed part-time
4 Currently a student, not employed
5 Currently a student, employed full or part-time
6 Self employed
8 Refuse to Answer

A10. Which of these is closest to your individual yearly income, before taxes? (Choose one)
00 No income
01 $1 to $9999
02 $10,000 to 19,999
03 $20,000 to 29,999
04 $30,000 to 39,999
05 $40,000 to 49,999
06 $50,000 to 59,999
07 $60,000 to 99,999
08 $100,000 or more
98 Refuse to Answer

A11. Including yourself, how many people depend on this income?
— —

A12. What is your primary type of health insurance? (Choose one)
1 No health insurance
2 Private, (HMO, PPO, Kaiser, Blue Shield, Aetna, etcetera)
3 Student health insurance
4 Medicare or MediCal or Medicaid
5 Healthy SF
6 Veterans' Administration
7 Other
8 Refuse to Answer

If A12 is not equal to 7, then skip to A13.
A12a. Please specify your primary health insurance
— —

A13. Do you have a regular health care provider?
1 Yes
0 No
8 Refuse to Answer

A14. Have you ever been homeless?
1 Yes
0 No

A15. **Since [DATE1]** (that is, in the last [DAYS] days), where did you sleep **most of the time**? *(Choose Only One)*
00 In my own house or apartment
01 In someone else's house or apartment
02 Rented room (hotel or rooming house)
03 Car, bus, truck, or other vehicle
04 Abandoned building
05 Shelter
06 Correctional institution (jail, juvenile detention center)
07 Drug treatment center
08 On the streets
09 Shooting gallery
10 Medical care facility (hospital, hospice, nursing home)
11 Other (specify)
98 Refuse to Answer

If A15 is not equal to 11, then skip to instruction before B16B.
A16. Please specify, since [DATE1] (that is, in the last [DAYS] days) where did you sleep most of the time?
— —

DRUG USE AND DRUG RELATED HIV RISK BEHAVIOR

B34a. **In your lifetime**, which drugs or alcohol have you ever used not as prescribed? *(Check all that apply)*

__Heroin
__Prescription opioids
__Alcohol
__Benzodiazepines (Valium, Ativan, Klonopin, etc)
__Crack Cocaine
__Powder Cocaine
__Methamphetamines/amphetamine (Crystal, Speed, Tina)
__Marijuana
__Ecstasy (E, X, MDMA)
__GHB (G, GBL)
__Hallucinogens (LSD, mushrooms, Peyote, or Mescaline)
__PCP (Angel Dust, wet, wicky sticks)
__Poppers (Amyl Nitrate)
__Rohypnol (Roofies)
__Ketamine (Special K)
__Other Tranquilizers, Barbiturates
__Viagra or similar drugs (Levitra, Cialis)
__Bath salts
__Other

Please specify, **in your lifetime**, what other drug(s) have you ever used not as prescribed?
— —

B34b. **In the last 30 days**, which drugs or alcohol have you ever used not as prescribed? *(Check all that apply)*

__Heroin
__Prescription opioids
__Alcohol
__Benzodiazepines (Valium, Ativan, Klonopin, etc)
__Crack Cocaine
__Powder Cocaine
__Methamphetamines/amphetamine (Crystal, Speed, Tina)
__Marijuana
__Ecstasy (E, X, MDMA)
__GHB (G, GBL)
__Hallucinogens (LSD, mushrooms, Peyote, or Mescaline)
__PCP (Angel Dust, wet, wicky sticks)
__Poppers (Amyl Nitrate)
__Rohypnol (Roofies)
__Ketamine (Special K)
__Other Tranquilizers, Barbiturates
__Viagra or similar drugs (Levitra, Cialis)
__Bath salts
__Other

Please specify, **in the last 30 days**, what other drug(s) have you ever used not as prescribed?
— —

B35a. **In your lifetime**, which drugs or alcohol have you ever injected? *(Check all that apply)*
__Heroin
__Prescription opioids
__Alcohol
__Benzodiazepines (Valium, Ativan, Klonopin, etc)
__Crack Cocaine
__Powder Cocaine
__Methamphetamines/amphetamine (Crystal, Speed, Tina)
__Marijuana
__Ecstasy (E, X, MDMA)
__GHB (G, GBL)
__Hallucinogens (LSD, mushrooms, Peyote, or Mescaline)
__PCP (Angel Dust, wet, wicky sticks)
__Poppers (Amyl Nitrate)
__Rohypnol (Roofies)
__Ketamine (Special K)
__Other Tranquilizers, Barbiturates
__Viagra or similar drugs (Levitra, Cialis)
__Bath salts
__Other

Please specify, **in your lifetime**, what other drug(s) have you ever injected?
__ __

B35b. **In the last 30 days**, which drugs or alcohol have you ever injected? *(Check all that apply)*
__Heroin
__Prescription opioids
__Alcohol
__Benzodiazepines (Valium, Ativan, Klonopin, etc)
__Crack Cocaine
__Powder Cocaine
__Methamphetamines/amphetamine (Crystal, Speed, Tina)
__Marijuana
__Ecstasy (E, X, MDMA)
__GHB (G, GBL)
__Hallucinogens (LSD, mushrooms, Peyote, or Mescaline)
__PCP (Angel Dust, wet, wicky sticks)
__Poppers (Amyl Nitrate)
__Rohypnol (Roofies)
__Ketamine (Special K)
__Other Tranquilizers, Barbiturates
__Viagra or similar drugs (Levitra, Cialis)
__Bath salts
__Other

Please specify, **in the last 30 days**, what other drug(s) have you ever injected?
__ __

B36. **In the last 30 days,** on average, how often did you inject any drug?

00	Never
01	Less than once a month
02	1 day per month
03	2 days per month
04	3 days per month
05	1 day per week (4 days per month)
06	2 days per week
07	3 days per week
08	4 days per week
09	5 days per week
10	6 days per week
11	Every day

SHARING NEEDLES AND INJECTION EQUIPMENT

The following questions are about your injection practices and use **since [DATE1]** (that is, in the last 30 days).

C133. **Since [DATE1]** (that is, in the last [DAYS] days), on AVERAGE, how often did you inject using syringes/needles that you know had been used by somebody else? (Choose one)

00	Never
01	Less than once a month
02	1 day per month
03	2 days per month
04	3 days per month
05	1 day per week (4 days per month)
06	2 days per week
07	3 days per week
08	4 days per week
09	5 days per week
10	6 days per week
11	Every day
97	Don't Know
98	Refuse to Answer

If C133 is greater than SINJFREQ then This number cannot be greater than the average number of times you injected since &[DATE1]. and skip to C133.
If C133 is equal to 0, then skip to C135.

C134. **Since [DATE1]** (that is, in the last [DAYS] days), with how many **different** people have you shared needles/syringes?

__ __ __	People
997	Don't Know
998	Refuse to Answer

C135. **Since [DATE1]** (that is, in the last [DAYS] days), on AVERAGE, how often did you use a cooker/cotton/rinse water that has been used by someone else?
(Choose one) (Choose one)

00	Never
01	Less than once a month
02	1 day per month
03	2 days per month
04	3 days per month
05	1 day per week (4 days per month)
06	2 days per week
07	3 days per week
08	4 days per week
09	5 days per week
10	6 days per week
11	Every day
97	Don't Know
98	Refuse to Answer

If C135 is greater than SINJFREQ then This number cannot be greater than the average number of times you injected since &[DATE1]. and skip to C135.

C136. **Since [DATE1]** (that is, in the last [DAYS] days), on AVERAGE how often did you fix drugs with another person and then split the drug solution (through use of the same cooker/spoon or through back loading)?
(Choose one) (Choose one)

00	Never
01	Less than once a month
02	1 day per month
03	2 days per month
04	3 days per month
05	1 day per week (4 days per month)
06	2 days per week
07	3 days per week
08	4 days per week
09	5 days per week
10	6 days per week
11	Every day
97	Don't Know
98	Refuse to Answer

If C136 is greater than SINJFREQ then This number cannot be greater than the average number of times you injected since &[DATE1]. and skip to C136.

DRUG CESSATION

If B53 is equal to 0, then skip to instruction before E141.

D137. **Since [DATE1]** (that is, in the last [DAYS] days), what is the longest amount of time (days in a row) that you went without using **any drugs**?

__ __ __	Days
998	Refuse to Answer

If D137 is greater than DAYS then This number cannot be greater than &[DAYS]. and skip to D137.

If D137 is less than 2 or D137 is equal to 998, then skip to instruction before E141.

D138. **Since [DATE1]** (that is, in the last [DAYS] days), how many **times** did you not use **opiates** for 2 or more days at any time?

__ __ __	Times
998	Refuse to Answer

If D138 is equal to 0, then skip to instruction before E141.

D139. **Since [DATE1]** (that is, in the last [DAYS] days), when was the most recent time you went without using opiates for 2 days or more?
(If you don't know the exact day, make your best guess.)

__ __ / __ __ / __ __ __ __ mm / dd / yyyy

If D139 is less than DATE1N then This date must be after &[DATE1], (that is, in the last &[DAYS] days). and skip to D139.

D140. After that time on [Response to D139], how much did you use when you started using opiates again? (Choose one)

0	About the same
1	More
2	Less
7	Don't Know

ROUTE SWITCHING

If B58 is not equal to 1 and B61 is not equal to 1 and B64 is not equal to 1 and B67 is not equal to 1 and B70 is not equal to 1 and B73 is not equal to 1 and B76 is not equal to 1 and B79 is not equal to 1 and B82 is not equal to 1 and B85 is not equal to 1 and B88 is not equal to 1 and B91 is not equal to 1 and B94 is not equal to 1 and B100 is not equal to 1 and B102 is not equal to 1 and B104 is not equal to 1 and B106 is not equal to 1 and B109 is not equal to 1 and B111 is not equal to 1 and B114 is not equal to 1 and B117 is not equal to 1 and B119 is not equal to 1 and B121 is not equal to 1 and B123 is not equal to 1 and B125 is not equal to 1 and B127 is not equal to 1, then skip to instruction before F142.

E141. **Since [DATE1]** (that is, in the last [DAYS] days), did you switch from injecting to other ways of using drugs (like snorting, smoking or swallowing) for 7 days or longer for any reason?

1 Yes
0 No

OPIATE OVERDOSE RISK BEHAVIORS

If B53 is equal to 0, then skip to instruction before G153.

The following questions are about how you have used opiates **since [DATE1]** (that is, in the last [DAYS] days).

Since [DATE1] (that is, in the last [DAYS] days), you reported that the most frequent opiate you used was [FQOPIATE] and that most days you used about [QNOPIATE] [UNIT].

F142. **Since [DATE1]** (that is, in the last [DAYS] days), on days that you used [FQOPIATE], how often (what percentage) did you use MORE than [QNOPIATE] [UNIT]?
(Use the slider or enter a value below to indicate the percentage of days that you used MORE than your average amount.)

00	Never (0)
01	
02	
03	
04	
05	
06	
07	
08	
09	
10	
11	
12	
13	
14	
15	
16	
17	
18	
19	
20	
21	
22	
23	
24	
25	
26	
27	
28	
29	
30	
31	
32	
33	
34	
35	
36	
37	
38	
39	
40	
41	
42	
43	
44	
45	
46	
47	
48	
49	
50	Half of the Times (50)

F143. **Since [DATE1]** (that is, in the last [DAYS] days), on days that you used [FQOPIATE], how often (what percentage) did you use LESS than [QNOPIATE] [UNIT]?
(Use the slider or enter a value below to indicate the percentage of days that you used LESS than your average amount.)

00	Never (0)
01	
02	
03	
04	
05	
06	
07	
08	
09	
10	
11	
12	
13	
14	
15	
16	
17	
18	
19	
20	
21	
22	
23	
24	
25	
26	
27	
28	
29	
30	
31	
32	
33	
34	
35	
36	
37	
38	
39	
40	
41	
42	
43	
44	
45	
46	
47	
48	
49	
50	Half of the Times (50)

If FCOPIATE is equal to 11, then skip to instruction before F145.

F144. **Since [DATE1]** (that is, in the last [DAYS] days), you reported using heroin or other opiates on at least [DSOPIATE] day(s) in the last [DAYS] days. On how many days did you use **ANY opiate**?

__ __ __ Days

If F144 is less than DSOPIATE then You cannot have used opiates on fewer days than the number of days that you reported using your most frequent opiate. and skip to instruction before F144.

If B96B is not equal to 1, then skip to instruction before F146.

F145. You reported using heroin or another opiate on about [OPIATDYS] days in the last [DAYS] days. How many day(s) did you use benzodiazepines **at the same time or within 2 hours** of using heroin or another opiate? Benzodiazepines include drugs like Valium, Ativan, Xanax, klonopin, lorazepam, and others.

__ __ __ Days

If FCOPIATE is not equal to 11 and F145 is greater than F144 or FCOPIATE is equal to 11 and F145 is greater than DSOPIATE then You cannot have used benzodiazepines at the same time or within 2 hours of having used heroin or another opiate more times than the number of day(s) that you reported using any opiates. and skip to F145.

If B96C is not equal to 1, then skip to instruction before F147.

F146. You reported using heroin or another opiate on about [OPIATDYS] days in the last [DAYS] days. How many day(s) did you use crack cocaine **at the same time or within 2 hours** of using heroin or another opiate?

__ __ __ Days

If FCOPIATE is not equal to 11 and F146 is greater than F144 or FCOPIATE is equal to 11 and F146 is greater than DSOPIATE then You cannot have used crack cocaine at the same time or within 2 hours of having used heroin or another opiate more times than the number of day(s) that you reported using any opiates. and skip to F146.

If B96D is not equal to 1, then skip to instruction before F148.

F147. You reported using heroin or another opiate on about [OPIATDYS] days in the last [DAYS] days. How many day(s) did you use powder cocaine **at the same time or within 2 hours** of using heroin or another opiate?

__ __ __ Days

If FCOPIATE is not equal to 11 and F147 is greater than F144 or FCOPIATE is equal to 11 and F147 is greater than DSOPIATE then You cannot have used cocaine at the same time or within 2 hours of having used heroin or another opiate more times than the number of day(s) that you reported using any opiates. and skip to F147.

If B96A is not equal to 1, then skip to instruction before F149.

F148. You reported using heroin or another opiate on about [OPIATDYS] days in the last [DAYS] days. How many day(s) did you use alcohol **at the same time or within 2 hours** of using heroin or another opiate?

__ __ __ Days

If FCOPIATE is not equal to 11 and F148 is greater than F144 or FCOPIATE is equal to 11 and F148 is greater than DSOPIATE then You cannot have used alcohol at the same time or within 2 hours of having used heroin or another opiate more times than the number of day(s) that you reported using any opiates. and skip to F148.

If B96E is not equal to 1, then skip to instruction before F150.

F149. You reported using heroin or another opiate on about [OPIATDYS] days in the last [DAYS] days. How many day(s) did you use methamphetamines/ amphetamine (Crystal, Speed, Tina) **at the same time or within 2 hours** of using heroin or another opiate?

__ __ __ Days

If FCOPIATE is not equal to 11 and F149 is greater than F144 or FCOPIATE is equal to 11 and F149 is greater than DSOPIATE then You cannot have used methamphetamines at the same time or within 2 hours of having used heroin or another opiate more times than the number of day(s) that you reported using any opiates. and skip to F149.

F150. **Since [DATE1]** (that is, in the last [DAYS] days), how many of the [FQOPTXT] day(s) that you used heroin or opiates did you use alone, without anyone else around?

__ __ __ Days

If F150 is greater than FQOPTXT then This number cannot be greater than &[FQOPTXT] and skip to F150.

If F150 is equal to 0, then skip to F152.

F151. **Since [DATE1]** (that is, in the last [DAYS] days), of the [Response to F150] day(s) that you used heroin or opiates alone, how many of those days did somebody know that you were using and where you were at that moment?

__ __ __ Days

If F151 is greater than F150 then This number cannot be greater than the number of days that you used heroin or other opiates alone. and skip to F151.

F152. **Since [DATE1]** (that is, in the last [DAYS] days), of the [FQOPTXT] day(s) that you used heroin or opiates, how many of those days did you use opiates in a place that you had **NEVER** used in before, such as a different house or apartment, or a different public space?

__ __ __ Days

If FCOPIATE is not equal to 11 and F152 is greater than F144 or FCOPIATE is equal to 11 and F152 is greater than DSOPIATE then This number cannot be greater than the number of days that you used heroin or other opiates. and skip to F152.

EGOCENTRIC NETWORK/ PARTNER BY PARTNER QUESTIONS

If B53 is equal to 0 and B95 is equal to 0, then skip to instruction before H238.

The following questions are about people that you have used drugs with **since [DATE1]** (that is, in the last [DAYS] days).

If B53 is equal to 0, then skip to instruction before G154.

G153. **Since [DATE1]** (that is, in the last [DAYS] days), how many people have you **used opiates with not as prescribed**? Include people who you injected opiates with as well as people you used non-injection opiates with.

__ __ People

If G153 is not equal to 0, then skip to G155.

If B95 is equal to 0, then skip to instruction before G234.

G154. **Since [DATE1]** (that is, in the last [DAYS] days), how many people have you **used any non-opiate drug with**? (DO NOT INCLUDE PEOPLE YOU **ONLY** USE ALCOHOL WITH.) Include individuals who were present when you used any non-opiate drugs, like cocaine, methamphetamine, or other illicit drugs.

__ __ People

If G154 is equal to 0, then skip to instruction before G234.

Now I'm going to ask you a few questions about your experiences with the [DRPERSON] that you have used non-opiate drugs with most recently.

Since [DATE1] (that is, in the last [DAYS] days), think about the most recent person you used non-opiate drugs with. Throughout this next group of questions, this **MOST RECENT DRUG USE PARTNER** will be referred to by his/her initials.

If 1 is equal to 1, then skip to G159.

G155. Of these [Response to G153] person(s), how many have ever enrolled in **this study** as far as you know?

__ __ People
97 Don't Know

If G155 is greater than G153 then This number cannot be greater than the number of opiate partners you have had since &[DATE1]. and skip to G155.

If G155 is equal to 0 or G155 is equal to 97 or Q3a is equal to 1, then skip to instruction before G159.

If G155 is equal to 1, then skip to G156.

For the next few questions, think about the person you are the closest to that is also in this study.

G156. **Since [DATE1]** (that is, in the last [DAYS] days), what kind of relationship did you have with this person? (Choose one)

0 Primary partner or lover
1 Friend
2 Casual sex partner/ trick/ fuck buddy
3 Acquaintance
4 Relative
5 Other
7 Don't Know
8 Refuse to Answer

G157. **Since [DATE1]** (that is, in the last [DAYS] days), how often did you see this person? (Choose one)

0 Never
1 Once a month or less
2 2-3 days a month
3 About once a month
4 2-3 days a week
5 4-6 days a week
6 Everyday
7 Don't Know
8 Refuse to Answer

G158. **Since [DATE1]** (that is, in the last [DAYS] days), how emotionally close were you to this person? On a scale of 0 to 10 with 0 being "not close at all" and 10 being "extrememly close."

00 Not at all close
01
02
03
04
05
06
07
08
09
10 Extremely Close
97 Don't Know
98 Refuse to Answer

Now I'm going to ask you a few questions about your experiences with the [OPPERSON] that you have used opiates with most recently.

Since [DATE1] (that is, in the last [DAYS] days), think about the most recent person you used heroin or opiates with. Throughout this next group of questions, this **MOST RECENT DRUG USE PARTNER** will be referred to by his/her initials.

G159. Please assign or make-up initials for this person here. Use AA if you do not know his/her name or choose not to use their initials.

__ __

G160. What is [Response to G159]'s gender identity? (Choose one)

0 Male
1 Female
2 Transfemale
3 Transmale
4 Other
7 Don't Know

G161. What is [Response to G159]'s race/ethnicity? (Choose one)

0 African American
1 Asian or Pacific Islander
2 Caucasian
3 Hispanic/ Latino
4 Mixed race/ ethnicity
5 Other
7 Don't Know

G162. How old is [Response to G159]? (If you are not sure, give your best guess.)

__ __ Years

G163. What is [Response to G159]'s HIV-status? (Choose one)

0 HIV-negative
1 HIV-positive
7 Don't Know

G164. What is [Response to G159]'s Hepatitis C status? (Choose one)

0	Does not have HCV infection
1	Has HCV infection
7	Don't Know

G165. As far as you know, has [Response to G159] ever enrolled in this study?

1	Yes
0	No
7	Don't Know

G166. What neighborhood does [Response to G159] hang out in most of the time? (Choose one)

00	Bayview / Hunters Point
01	Bernal Heights
02	Castro
03	Civic Center
04	Excelsior
05	Haight Ashbury / Lower Haight
06	Mission
07	Nob Hill
08	North Beach
09	Potrero Hill / Mission Bay
10	Richmond (Inner and Outer)
11	Russian Hill / Pacific Heights
12	SOMA
13	Sunset (Inner and Outer)
14	Tenderloin
15	Twin Peaks
16	Union Square / Chinatown
17	Visitacion Valley
18	Western Addition
19	Other San Francisco neighborhood
20	East Bay
21	Other
97	Don't Know

G167. What kind of partner is [Response to G159]? (Choose one)

0	Friend
1	Family
2	Sex partner
3	Stranger
4	Other
5	Primary Partner or Spouse

If G153 is not greater than 0, then skip to G170.

G168. **Since [DATE1]** (that is, in the last [DAYS] days), what opiates does [Response to G159] use not as prescribed?
(Check all that apply) (Check all that apply)

__ Heroin
__ Oxymorphone (Opana)
__ Morphine (MSContin, Kadian, Embeda, Avinza)
__ Hydrocodone (Vicodin, Lorcet, Lortab, Norco, Zohydro)
__ Fentanyl (Duragesic)
__ Hydromorphone (Dilaudid, Palladone)
__ Oxycodone (Percocet, OxyContin, Roxicodone, Percodan)
__ Methadone (Dolophine)
__ Buprenorphine (Suboxone, Subutex)
__ Propoxyphene (Darvocet)
__ Meperidine (Demerol)
__ Codeine (Tylenol with codeine, TyCo, Tylenol #3)
__ Other
__ Don't Know

If G168M is not equal to 1, then skip to instruction before G170.

G169. Please specify, since [DATE1] (that is, in the last [DAYS] days), what other opiate(s) has [Response to G159] used not as prescribed?

____

If G168 is equal to 97, then skip to G177.
If 1 is equal to 1, then skip to G172.

G170. **Since [DATE1]** (that is, in the last [DAYS] days), what non-opiate drugs has [Response to G159] used not as prescribed? (This includes alcohol.)
(Check all that apply)
(Check all that apply)

__ Alcohol
__ Benzodiazepines (Valium, Ativan, Klonopin, etc)
__ Crack Cocaine
__ Powder Cocaine
__ Methamphetamines/amphetamine (Crystal, Speed, Tina)
__ Marijuana
__ Ecstasy (E, X, MDMA)
__ GHB (G, GBL)
__ Hallucinogens (LSD, mushrooms, Peyote, or Mescaline)
__ PCP (Angel Dust, wet, wicky sticks)
__ Poppers (Amyl Nitrate)
__ Rohypnol (Roofies)
__ Ketamine (Special K)
__ Other Tranquilizers, Barbiturates
__ Viagra or similar drugs (Levitra, Cialis)
__ Bath salts
__ Other
__ Don't Know

If G170Q is not equal to 1, then skip to instruction before G172.

G171. Please specify, since [DATE1] (that is, in the last [DAYS] days), what other drug(s) has [Response to G159] used not as prescribed.

____

If G170 is equal to 97, then skip to G177.

G172. **Since [DATE1]** (that is, in the last [DAYS] days), has [Response to G159] **<u>INJECTED</u>** drugs?

1 Yes
0 No
7 Don't Know

If G172 is not equal to 1, then skip to G177.
If G153 is not greater than 0, then skip to G175.

G173. **Since [DATE1]** (that is, in the last [DAYS] days), what opiate(s) has [Response to G159] **<u>INJECTED</u>**?
(Check all that apply) (Check all that apply)

__ Heroin
__ Oxymorphone (Opana)
__ Morphine (MSContin, Kadian, Embeda, Avinza)
__ Hydrocodone (Vicodin, Lorcet, Lortab, Norco, Zohydro)
__ Fentanyl (Duragesic)
__ Hydromorphone (Dilaudid, Palladone)
__ Oxycodone (Percocet, OxyContin, Roxicodone, Percodan)
__ Methadone (Dolophine)
__ Buprenorphine (Suboxone, Subutex)
__ Propoxyphene (Darvocet)
__ Meperidine (Demerol)
__ Codeine (Tylenol with codeine, TyCo, Tylenol #3)
__ Other Opiate(s)
__ Other Non-Opiate Drug(s)
__ Don't Know

If G173A is equal to 1 and G168A is equal to 0 or G173B is equal to 1 and G168B is equal to 0 or G173C is equal to 1 and G168C is equal to 0 or G173D is equal to 1 and G168D is equal to 0 or G173E is equal to 1 and G168E is equal to 0 or G173F is equal to 1 and G168F is equal to 0 or G173G is equal to 1 and G168G is equal to 0 or G173H is equal to 1 and G168H is equal to 0 or G173I is equal to 1 and G168I is equal to 0 or G173J is equal to 1 and G168J is equal to 0 or G173K is equal to 1 and G168K is equal to 0 or G173L is equal to 1 and G168L is equal to 0 or G173M is equal to 1 and G168M is equal to 0 then &[NWNAME1] cannot have injected a drug that you reported s/he hasn't used. and skip to instruction before G168.
If G173L is not equal to 1, then skip to instruction before G175.

G174. Please specify, since [DATE1] (that is, in the last [DAYS] days), what other opiate(s) has [Response to G159] injected not as prescribed?

____

If 1 is equal to 1, then skip to G177.

G175. **Since [DATE1]** (that is, in the last [DAYS] days), what non-opiate drugs has [Response to G159] **INJECTED**?
(Check all that apply) (Check all that apply)

__ Benzodiazepines (Valium, Ativan, Klonopin, etc)
__ Crack Cocaine
__ Powder Cocaine
__ Methamphetamines/amphetamine (Crystal, Speed, Tina)
__ **Ecstasy (E, X, MDMA)**
__ **GHB (G, GBL)**
__ **PCP (Angel Dust, wet, wicky sticks)**
__ **Rohypnol (Roofies)**
__ **Ketamine (Special K)**
__ Other Tranquilizers, Barbiturates
__ Viagra or similar drugs (Levitra, Cialis)
__ Bath salts
__ Other
__ Don't Know

If G175B is equal to 1 and G170B is equal to 0 or G175C is equal to 1 and G170C is equal to 0 or G175D is equal to 1 and G170D is equal to 0 or G175E is equal to 1 and G170E is equal to 0 or G175G is equal to 1 and G170G is equal to 0 or G175H is equal to 1 and G170H is equal to 0 or G175J is equal to 1 and G170J is equal to 0 or G175L is equal to 1 and G170L is equal to 0 or G175M is equal to 1 and G170M is equal to 0 or G175N is equal to 1 and G170N is equal to 0 or G175O is equal to 1 and G170O is equal to 0 or G175P is equal to 1 and G170P is equal to 0 or G175Q is equal to 1 and G170Q is equal to 0 then &[NWNAME1] cannot have injected a drug that you reported s/he hasn't used. and skip to instruction before G168.

If G175Q is not equal to 1, then skip to G177.

G176. Please specify, since [DATE1] (that is, in the last [DAYS] days), what other non-opiate drug(s) has [Response to G159] injected?

__ __

G177. **Since [DATE1]** (that is, in the last [DAYS] days), how many times have you used any drugs with [Response to G159]? (Remember, we are asking about the number of TIMES, not the number of DAYS for this question.)

__ __ __ Times
997 Don't Know

If B58 is not equal to 1 and B61 is not equal to 1 and B64 is not equal to 1 and B67 is not equal to 1 and B70 is not equal to 1 and B73 is not equal to 1 and B76 is not equal to 1 and B79 is not equal to 1 and B82 is not equal to 1 and B85 is not equal to 1 and B88 is not equal to 1 and B91 is not equal to 1 and B94 is not equal to 1 and B100 is not equal to 1 and B102 is not equal to 1 and B104 is not equal to 1 and B106 is not equal to 1 and B109 is not equal to 1 and B111 is not equal to 1 and B114 is not equal to 1 and B117 is not equal to 1 and B119 is not equal to 1 and B121 is not equal to 1 and B123 is not equal to 1 and B125 is not equal to 1 and B127 is not equal to 1 or G172 is not equal to 1, then skip to instruction before G180.

G178. **Since [DATE1]** (that is, in the last [DAYS] days), how many times have you **INJECTED** with [Response to G159]? (Remember, we are asking about the number of TIMES, not the number of DAYS for this question.)

__ __ __ Times
997 Don't Know

If G178 is greater than G177 then This number cannot be greater than the number of times you used drugs with &[NWNAME1] and skip to G178.

If G178 is equal to 0, then skip to instruction before G180.

G179. **Since [DATE1]** (that is, in the last [DAYS] days), have you shared needles/syringes with [Response to G159]?

1 Yes
0 No
7 Don't Know

If G153 is not greater than 0, then skip to instruction before G181.

G180. **Since [DATE1]** (that is, in the last [DAYS] days), has [Response to G159] had an opiate overdose?

1 Yes
0 No
7 Don't Know

If G180 is not equal to 1, then skip to G183.

G181. Did you observe [Response to G159] when he/she had an opiate overdose?

1 Yes
0 No
7 Don't Know

If G181 is not equal to 1, then skip to G183.

G182. Did you administer naloxone to [Response to G159] when they had an opiate overdose?

1 Yes
0 No
7 Don't Know

G183. Does [Response to G159] carry naloxone?

1 Yes
0 No
7 Don't Know

If NETSIZE is less than 2, then skip to instruction before H238.
If G153 is equal to 0, then skip to instruction before G184.

Since [DATE1] (that is, in the last [DAYS] days), think about the second most recent person you used heroin or opiates with. Throughout this next group of questions, this **SECOND MOST RECENT DRUG USE PARTNER** will be referred to by his/her initials.

If G153 is greater than 0, then skip to G184.

Since [DATE1] (that is, in the last [DAYS] days), think about the second most recent person you used non-opiate drugs with. Throughout this next group of questions, this **SECOND MOST RECENT DRUG USE PARTNER** will be referred to by their initials.

G184. Please assign or make-up initials for this person here. Use BB if you do not know his/her name or choose not to use their initials.

__ __

G185. What is [Response to G184]'s gender identity? (Choose one)

0 Male
1 Female
2 Transfemale
3 Transmale
4 Other
7 Don't Know

G186. What is [Response to G184]'s race/ethnicity? (Choose one)

0 African American
1 Asian or Pacific Islander
2 Caucasian
3 Hispanic/ Latino
4 Mixed race/ ethnicity
5 Other
7 Don't Know

G187. How old is [Response to G184]? (If you are not sure, give your best guess.)

__ __ Years

G188. What is [Response to G184]'s HIV-status? (Choose one)

0 HIV-negative
1 HIV-positive
7 Don't Know

G189. What is [Response to G184]'s Hepatitis C status? (Choose one)

0 Does not have HCV infection
1 Has HCV infection
7 Don't Know

G190. As far as you know, has [Response to G184] ever enrolled in this study?

1 Yes
0 No
7 Don't Know

G191. What neighborhood does [Response to G184] hang out in most of the time? (Choose one)

00 Bayview / Hunters Point
01 Bernal Heights
02 Castro
03 Civic Center
04 Excelsior
05 Haight Ashbury / Lower Haight
06 Mission
07 Nob Hill
08 North Beach
09 Potrero Hill / Mission Bay
10 Richmond (Inner and Outer)
11 Russian Hill / Pacific Heights
12 SOMA
13 Sunset (Inner and Outer)
14 Tenderloin
15 Twin Peaks
16 Union Square / Chinatown
17 Visitacion Valley
18 Western Addition
19 Other San Francisco neighborhood
20 East Bay
21 Other
97 Don't Know

G192. What kind of partner is [Response to G184]? (Choose one)

0 Friend
1 Family
2 Sex partner
3 Stranger
4 Other
5 Primary Partner or Spouse

If G153 is not greater than 0, then skip to G195.

G193. **Since [DATE1]** (that is, in the last [DAYS] days), what opiates does [Response to G184] use not as prescribed?
(Check all that apply) (Check all that apply)

__ Heroin
__ Oxymorphone (Opana)
__ Morphine (MSContin, Kadian, Embeda, Avinza)
__ Hydrocodone (Vicodin, Lorcet, Lortab, Norco, Zohydro)
__ Fentanyl (Duragesic)
__ Hydromorphone (Dilaudid, Palladone)
__ Oxycodone (Percocet, OxyContin, Roxicodone, Percodan)
__ Methadone (Dolophine)
__ Buprenorphine (Suboxone, Subutex)
__ Propoxyphene (Darvocet)
__ Meperidine (Demerol)
__ Codeine (Tylenol with codeine, TyCo, Tylenol #3)
__ Other
__ Don't Know

If G193M is not equal to 1, then skip to instruction before G195.

G194. Please specify, since [DATE1] (that is, in the last [DAYS] days), what other opiate(s) has [Response to G184] used not as prescribed?

____

If G193 is equal to 97, then skip to G202.
If 1 is equal to 1, then skip to G197.

G195. **Since [DATE1]** (that is, in the last [DAYS] days), what non-opiate drugs has [Response to G184] used not as prescribed? (This includes alcohol.)
(Check all that apply)
(Check all that apply)

__ Alcohol
__ Benzodiazepines (Valium, Ativan, Klonopin, etc)
__ Crack Cocaine
__ Powder Cocaine
__ Methamphetamines/amphetamine (Crystal, Speed, Tina)
__ Marijuana
__ Ecstasy (E, X, MDMA)
__ GHB (G, GBL)
__ Hallucinogens (LSD, mushrooms, Peyote, or Mescaline)
__ PCP (Angel Dust, wet, wicky sticks)
__ Poppers (Amyl Nitrate)
__ Rohypnol (Roofies)
__ Ketamine (Special K)
__ Other Tranquilizers, Barbiturates
__ Viagra or similar drugs (Levitra, Cialis)
__ Bath salts
__ Other
__ Don't Know

If G195Q is not equal to 1, then skip to instruction before G197.

G196. Please specify, since [DATE1] (that is, in the last [DAYS] days), what other drug(s) has [Response to G184] used not as prescribed.

____

If G195 is equal to 97, then skip to G202.

G197. **Since [DATE1]** (that is, in the last [DAYS] days), has [Response to G184] **INJECTED** drugs?

1 Yes
0 No
7 Don't Know

If G197 is not equal to 1, then skip to G202.
If G153 is not greater than 0, then skip to G200.

G198. **Since [DATE1]** (that is, in the last [DAYS] days), what opiate(s) has [Response to G184] **INJECTED**?
(Check all that apply) (Check all that apply)

__ Heroin
__ Oxymorphone (Opana)
__ Morphine (MSContin, Kadian, Embeda, Avinza)
__ Hydrocodone (Vicodin, Lorcet, Lortab, Norco, Zohydro)
__ Fentanyl (Duragesic)
__ Hydromorphone (Dilaudid, Palladone)
__ Oxycodone (Percocet, OxyContin, Roxicodone, Percodan)
__ Methadone (Dolophine)
__ Buprenorphine (Suboxone, Subutex)
__ Propoxyphene (Darvocet)
__ Meperidine (Demerol)
__ Codeine (Tylenol with codeine, TyCo, Tylenol #3)
__ Other Opiate(s)
__ Other Non-Opiate Drug(s)
__ Don't Know

If G198A is equal to 1 and G193A is equal to 0 or G198B is equal to 1 and G193B is equal to 0 or G198C is equal to 1 and G193C is equal to 0 or G198D is equal to 1 and G193D is equal to 0 or G198E is equal to 1 and G193E is equal to 0 or G198F is equal to 1 and G193F is equal to 0 or G198G is equal to 1 and G193G is equal to 0 or G198H is equal to 1 and G193H is equal to 0 or G198I is equal to 1 and G193I is equal to 0 or G198J is equal to 1 and G193J is equal to 0 or G198K is equal to 1 and G193K is equal to 0 or G198L is equal to 1 and G193L is equal to 0 or G198M is equal to 1 and G193M is equal to 0 then &[NWNAME2] cannot have injected a drug that you reported s/he hasn't used. and skip to instruction before G193.
If G198L is not equal to 1, then skip to instruction before G200.

G199. Please specify, since [DATE1] (that is, in the last [DAYS] days), what other opiate(s) has [Response to G184] injected not as prescribed?

__ __

If 1 is equal to 1, then skip to G202.

G200. **Since [DATE1]** (that is, in the last [DAYS] days), what non-opiate drugs has [Response to G184] **INJECTED**?
(Check all that apply) (Check all that apply)

__ Benzodiazepines (Valium, Ativan, Klonopin, etc)
__ Crack Cocaine
__ Powder Cocaine
__ Methamphetamines/amphetamine (Crystal, Speed, Tina)
__ **Ecstasy (E, X, MDMA)**
__ **GHB (G, GBL)**
__ **PCP (Angel Dust, wet, wicky sticks)**
__ **Rohypnol (Roofies)**
__ **Ketamine (Special K)**
__ Other Tranquilizers, Barbiturates
__ Viagra or similar drugs (Levitra, Cialis)
__ Bath salts
__ Other
__ Don't Know

If G200B is equal to 1 and G195B is equal to 0 or G200C is equal to 1 and G195C is equal to 0 or G200D is equal to 1 and G195D is equal to 0 or G200E is equal to 1 and G195E is equal to 0 or G200G is equal to 1 and G195G is equal to 0 or G200H is equal to 1 and G195H is equal to 0 or G200J is equal to 1 and G195J is equal to 0 or G200L is equal to 1 and G195L is equal to 0 or G200M is equal to 1 and G195M is equal to 0 or G200N is equal to 1 and G195N is equal to 0 or G200O is equal to 1 and G195O is equal to 0 or G200P is equal to 1 and G195P is equal to 0 or G200Q is equal to 1 and G195Q is equal to 0 then &[NWNAME2] cannot have injected a drug that you reported s/he hasn't used. and skip to instruction before G193.
If G200Q is not equal to 1, then skip to G202.

G201. Please specify, since [DATE1] (that is, in the last [DAYS] days), what other non-opiate drug(s) has [Response to G184] injected?

__ __

G202. **Since [DATE1]** (that is, in the last [DAYS] days), how many times have you used any drugs with [Response to G184]? (Remember, we are asking about the number of TIMES, not the number of DAYS for this question.)

__ __ __ Times
997 Don't Know

If B58 is not equal to 1 and B61 is not equal to 1 and B64 is not equal to 1 and B67 is not equal to 1 and B70 is not equal to 1 and B73 is not equal to 1 and B76 is not equal to 1 and B79 is not equal to 1 and B82 is not equal to 1 and B85 is not equal to 1 and B88 is not equal to 1 and B91 is not equal to 1 and B94 is not equal to 1 and B100 is not equal to 1 and B102 is not equal to 1 and B104 is not equal to 1 and B106 is not equal to 1 and B109 is not equal to 1 and B111 is not equal to 1 and B114 is not equal to 1 and B117 is not equal to 1 and B119 is not equal to 1 and B121 is not equal to 1 and B123 is not equal to 1 and B125 is not equal to 1 and B127 is not equal to 1 or G172 is not equal to 1, then skip to instruction before G205.

G203. **Since [DATE1]** (that is, in the last [DAYS] days), how many times have you **INJECTED** with [Response to G184]? (Remember, we are asking about the number of TIMES, not the number of DAYS for this question.)

__ __ __ Times
997 Don't Know

If G203 is greater than G202 then This number cannot be greater than the number of times you used drugs with &[NWNAME2] and skip to G203.
If G203 is equal to 0, then skip to instruction before G205.

G204. **Since [DATE1]** (that is, in the last [DAYS] days), have you shared needles/syringes with [Response to G184]?

1 Yes
0 No
7 Don't Know

If G153 is not greater than 0, then skip to instruction before G206.

G205. **Since [DATE1]** (that is, in the last [DAYS] days), has [Response to G184] had an opiate overdose?

1 Yes
0 No
7 Don't Know

If G205 is not equal to 1, then skip to G208.

G206. Did you observe [Response to G184] when he/she had an opiate overdose?

1 Yes
0 No
7 Don't Know

If G206 is not equal to 1, then skip to G208.

G207. Did you administer naloxone to [Response to G184] when they had an opiate overdose?

1 Yes
0 No
7 Don't Know

G208. Does [Response to G184] carry naloxone?

1 Yes
0 No
7 Don't Know

If NETSIZE is less than 3, then skip to instruction before H238.
If G153 is equal to 0, then skip to instruction before G209.

Since [DATE1] (that is, in the last [DAYS] days), think about the third most recent person you used heroin or opiates with. Throughout this next group of questions, this **THIRD MOST RECENT DRUG USE PARTNER** will be referred to by his/her initials.

If G153 is greater than 0, then skip to G209.

Since [DATE1] (that is, in the last [DAYS] days), think about the third most recent person you used non-opiate drugs with. Throughout this next group of questions, this **THIRD MOST RECENT DRUG USE PARTNER** will be referred to by their initials.

G209. Please assign or make-up initials for this person here. Use CC if you do not know his/her name or choose not to use their initials.

__ __

G210. What is [Response to G209]'s gender identity? (Choose one)

0 Male
1 Female
2 Transfemale
3 Transmale
4 Other
7 Don't Know

G211. What is [Response to G209]'s race/ethnicity? (Choose one)

0 African American
1 Asian or Pacific Islander
2 Caucasian
3 Hispanic/ Latino
4 Mixed race/ ethnicity
5 Other
7 Don't Know

G212. How old is [Response to G209]? (If you are not sure, give your best guess.)

__ __ Years

G213. What is [Response to G209]'s HIV-status? (Choose one)

0 HIV-negative
1 HIV-positive
7 Don't Know

G214. What is [Response to G209]'s Hepatitis C status? (Choose one)

0 Does not have HCV infection
1 Has HCV infection
7 Don't Know

G215. As far as you know, has [Response to G209] ever enrolled in this study?

1 Yes
0 No
7 Don't Know

G216. What neighborhood does [Response to G209] hang out in most of the time? (Choose one)

00 Bayview / Hunters Point
01 Bernal Heights
02 Castro
03 Civic Center
04 Excelsior
05 Haight Ashbury / Lower Haight
06 Mission
07 Nob Hill
08 North Beach
09 Potrero Hill / Mission Bay
10 Richmond (Inner and Outer)
11 Russian Hill / Pacific Heights
12 SOMA
13 Sunset (Inner and Outer)
14 Tenderloin
15 Twin Peaks
16 Union Square / Chinatown
17 Visitacion Valley
18 Western Addition
19 Other San Francisco neighborhood
20 East Bay
21 Other
97 Don't Know

G217. What kind of partner is [Response to G209]? (Choose one)

0 Friend
1 Family
2 Sex partner
3 Stranger
4 Other
5 Primary Partner or Spouse

If G153 is not greater than 0, then skip to G220.

G218. **Since [DATE1]** (that is, in the last [DAYS] days), what opiates does [Response to G209] use not as prescribed?
(Check all that apply) (Check all that apply)

__ Heroin
__ Oxymorphone (Opana)
__ Morphine (MSContin, Kadian, Embeda, Avinza)
__ Hydrocodone (Vicodin, Lorcet, Lortab, Norco, Zohydro)
__ Fentanyl (Duragesic)
__ Hydromorphone (Dilaudid, Palladone)
__ Oxycodone (Percocet, OxyContin, Roxicodone, Percodan)
__ Methadone (Dolophine)
__ Buprenorphine (Suboxone, Subutex)
__ Propoxyphene (Darvocet)
__ Meperidine (Demerol)
__ Codeine (Tylenol with codeine, TyCo, Tylenol #3)
__ Other
__ Don't Know

If G218M is not equal to 1, then skip to instruction before G220.

G219. Please specify, since [DATE1] (that is, in the last [DAYS] days), what other opiate(s) has [Response to G209] used not as prescribed?

__ __

If G218 is equal to 97, then skip to G227.
If 1 is equal to 1, then skip to G222.

G220. **Since [DATE1]** (that is, in the last [DAYS] days), what non-opiate drugs has [Response to G209] used not as prescribed? (This includes alcohol.)
(Check all that apply)
(Check all that apply)

__ Alcohol
__ Benzodiazepines (Valium, Ativan, Klonopin, etc)
__ Crack Cocaine
__ Powder Cocaine
__ Methamphetamines/amphetamine (Crystal, Speed, Tina)
__ Marijuana
__ Ecstasy (E, X, MDMA)
__ GHB (G, GBL)
__ Hallucinogens (LSD, mushrooms, Peyote, or Mescaline)
__ PCP (Angel Dust, wet, wicky sticks)
__ Poppers (Amyl Nitrate)
__ Rohypnol (Roofies)
__ Ketamine (Special K)
__ Other Tranquilizers, Barbiturates
__ Viagra or similar drugs (Levitra, Cialis)
__ Bath salts
__ Other
__ Don't Know

If G220Q is not equal to 1, then skip to instruction before G222.

G221. Please specify, since [DATE1] (that is, in the last [DAYS] days), what other drug(s) has [Response to G209] used not as prescribed.

__ __

If G220 is equal to 97, then skip to G227.

G222. **Since [DATE1]** (that is, in the last [DAYS] days), has [Response to G209] **INJECTED** drugs?

1 Yes
0 No
7 Don't Know

If G222 is not equal to 1, then skip to G227.
If G153 is not greater than 0, then skip to G225.

G223. **Since [DATE1]** (that is, in the last [DAYS] days), what opiate(s) has [Response to G209] **INJECTED**?

(Check all that apply) (Check all that apply)

__ Heroin
__ Oxymorphone (Opana)
__ Morphine (MSContin, Kadian, Embeda, Avinza)
__ Hydrocodone (Vicodin, Lorcet, Lortab, Norco, Zohydro)
__ Fentanyl (Duragesic)
__ Hydromorphone (Dilaudid, Palladone)
__ Oxycodone (Percocet, OxyContin, Roxicodone, Percodan)
__ Methadone (Dolophine)
__ Buprenorphine (Suboxone, Subutex)
__ Propoxyphene (Darvocet)
__ Meperidine (Demerol)
__ Codeine (Tylenol with codeine, TyCo, Tylenol #3)
__ Other Opiate(s)
__ Other Non-Opiate Drug(s)
__ Don't Know

If G223A is equal to 1 and G218A is equal to 0 or G223B is equal to 1 and G218B is equal to 0 or G223C is equal to 1 and G218C is equal to 0 or G223D is equal to 1 and G218D is equal to 0 or G223E is equal to 1 and G218E is equal to 0 or G223F is equal to 1 and G218F is equal to 0 or G223G is equal to 1 and G218G is equal to 0 or G223H is equal to 1 and G218H is equal to 0 or G223I is equal to 1 and G218I is equal to 0 or G223J is equal to 1 and G218J is equal to 0 or G223K is equal to 1 and G218K is equal to 0 or G223L is equal to 1 and G218L is equal to 0 or G223M is equal to 1 and G218M is equal to 0 then &[NWNAME3] cannot have injected a drug that you reported s/he hasn't used. and skip to instruction before G218.
If G223L is not equal to 1, then skip to instruction before G225.

G224. Please specify, since [DATE1] (that is, in the last [DAYS] days), what other opiate(s) has [Response to G209] injected not as prescribed?

__ __

If 1 is equal to 1, then skip to G227.

G225. **Since [DATE1]** (that is, in the last [DAYS] days), what non-opiate drugs has [Response to G209] **<u>INJECTED</u>**?
(Check all that apply) (Check all that apply)

__ Benzodiazepines (Valium, Ativan, Klonopin, etc)
__ Crack Cocaine
__ Powder Cocaine
__ Methamphetamines/amphetamine (Crystal, Speed, Tina)
__ **Ecstasy (E, X, MDMA)**
__ **GHB (G, GBL)**
__ **PCP (Angel Dust, wet, wicky sticks)**
__ **Rohypnol (Roofies)**
__ **Ketamine (Special K)**
__ Other Tranquilizers, Barbiturates
__ Viagra or similar drugs (Levitra, Cialis)
__ Bath salts
__ Other
__ Don't Know

If G225B is equal to 1 and G220B is equal to 0 or G225C is equal to 1 and G220C is equal to 0 or G225D is equal to 1 and G220D is equal to 0 or G225E is equal to 1 and G220E is equal to 0 or G225G is equal to 1 and G220G is equal to 0 or G225H is equal to 1 and G220H is equal to 0 or G225J is equal to 1 and G220J is equal to 0 or G225L is equal to 1 and G220L is equal to 0 or G225M is equal to 1 and G220M is equal to 0 or G225N is equal to 1 and G220N is equal to 0 or G225O is equal to 1 and G220O is equal to 0 or G225P is equal to 1 and G220P is equal to 0 or G225Q is equal to 1 and G220Q is equal to 0 then &[NWNAME3] cannot have injected a drug that you reported s/he hasn't used. and skip to instruction before G218.
If G225Q is not equal to 1, then skip to G227.

G226. Please specify, since [DATE1] (that is, in the last [DAYS] days), what other non-opiate drug(s) has [Response to G209] injected?

__ __

G227. **Since [DATE1]** (that is, in the last [DAYS] days), how many times have you used any drugs with [Response to G209]? (Remember, we are asking about the number of TIMES, not the number of DAYS for this question.)

__ __ __ Times
997 Don't Know

If B58 is not equal to 1 and B61 is not equal to 1 and B64 is not equal to 1 and B67 is not equal to 1 and B70 is not equal to 1 and B73 is not equal to 1 and B76 is not equal to 1 and B79 is not equal to 1 and B82 is not equal to 1 and B85 is not equal to 1 and B88 is not equal to 1 and B91 is not equal to 1 and B94 is not equal to 1 and B100 is not equal to 1 and B102 is not equal to 1 and B104 is not equal to 1 and B106 is not equal to 1 and B109 is not equal to 1 and B111 is not equal to 1 and B114 is not equal to 1 and B117 is not equal to 1 and B119 is not equal to 1 and B121 is not equal to 1 and B123 is not equal to 1 and B125 is not equal to 1 and B127 is not equal to 1 or G172 is not equal to 1, then skip to instruction before G230.

G228. **Since [DATE1]** (that is, in the last [DAYS] days), how many times have you **<u>INJECTED</u>** with [Response to G209]? (Remember, we are asking about the number of TIMES, not the number of DAYS for this question.)

__ __ __ Times
997 Don't Know

If G228 is greater than G227 then This number cannot be greater than the number of times you used drugs with &[NWNAME3] and skip to G228.
If G228 is equal to 0, then skip to instruction before G230.

G229. **Since [DATE1]** (that is, in the last [DAYS] days), have you shared needles/syringes with [Response to G209]?

1 Yes
0 No
7 Don't Know

If G153 is not greater than 0, then skip to instruction before G231.

G230. **Since [DATE1]** (that is, in the last [DAYS] days), has [Response to G209] had an opiate overdose?

1 Yes
0 No
7 Don't Know

If G230 is not equal to 1, then skip to G233.

G231. Did you observe [Response to G209] when he/she had an opiate overdose?

1 Yes
0 No
7 Don't Know

If G231 is not equal to 1, then skip to G233.

G232. Did you administer naloxone to [Response to G209] when they had an opiate overdose?

1 Yes
0 No
7 Don't Know

G233. Does [Response to G209] carry naloxone?

1 Yes
0 No
7 Don't Know

If 1 is equal to 1, then skip to instruction before H238.
If B95 is equal to 0 and B53 is equal to 0, then skip to instruction before H238.

G234. You reported that **since [DATE1]** (that is, in the last [DAYS] days), you have not used opiates or other drugs not as prescribed in the presence of anyone else. Is this correct? (Choose one)

0 No, this is not correct. I DID use opiates or other drugs with someone else.
1 Yes, this is correct. I did NOT use opiates or other drugs with someone else.

If G234 is equal to 0 then skip to instruction before G153.

G235. Do you wish you had someone to use drugs with?

1 Yes
0 No

G236. What are some reasons why you do not have someone to use drugs with? (Check all that apply)

__ No stable place to use drugs
__ Don't trust other people
__ Concerned about privacy
__ Don't want others to know I'm using
__ Other

If G236E is not equal to 1, then skip to instruction before H238.

G237. Please specify, what other reason(s) do you have for not having someone to use drugs with?

__ __
__ __
__ __
__ __
__ __
__ __
__ __

OPIATE OVERDOSE RISK PERCEPTION SCALE

Now we're going to ask you a few questions about how you feel about your opiate overdose risk.

H238. **Since [DATE1]** (that is, in the last [DAYS] days), how often have you worried about possibly overdosing from opiates? (Choose one)

0	Often
1	Sometimes
2	Rarely
3	Never

H239. **In the NEXT 4 months**, what do you think the chance is that you will witness somebody having an opiate overdose? (Choose one)

0	Very likely
1	Somewhat likely
2	Unlikely
3	Very unlikely

H240. **In the NEXT 4 months,** what do you think is your own chance of having an opiate overdose? (Choose one)

0	Very likely
1	Somewhat likely
2	Unlikely
3	Very unlikely

WITNESSED OPIATE OVERDOSE HISTORY

If Q3a is not equal to 1, then skip to instruction before I244.

Now we're going to ask you questions about opiate overdoses that you have witnessed.

I241. **In you lifetime**, how many times have you witnessed an opiate overdose? (If you don't recall exactly, make your best guess.)

__ __ __ Times

If I241 is equal to 0 and NETODS is greater than 0 then You have already reported that you witnessed an opiate overdose among your recent drug using partner(s). and skip to I241.

If I241 is equal to 0, then skip to I247.

I242. **In your lifetime**, how many times have you given naloxone to someone for an opiate overdose? This means you personally gave an injection or squirted it up their nose. (If you don't recall exactly, make your best guess.)

__ __ __ Times

I243. **In the past 12 months**, how many times have you witnessed an opiate overdose? (If you don't recall exactly, make your best guess.)

__ __ __ Times

If I243 is equal to 0 and NETODS is greater than 0 then You have already reported that you witnessed an opiate overdose among your recent drug using partner(s). and skip to I243.

If I243 is greater than I241 then The number of opiate overdoses you witnessed in the past 12 months cannot be greater than the number of opiate overdoses you witnessed in your lifetime. and skip to I243.

If I243 is equal to 0, then skip to instruction before I248.

The following questions are about opiate overdoses you have witnessed **since [DATE2]** (that is, in the last [DAYS2] days).

I244. **Since [DATE2]** (that is, in the last [DAYS2] days), how many times have you witnessed an opiate overdose? (If you don't recall exactly, make your best guess.)

__ __ __ Times

If I244 is equal to 0 and NETODS is greater than 0 then You have already reported that you witnessed an opiate overdose among your recent drug using partner(s). and skip to I244.

If I244 is greater than I243 and Q3a is equal to 1 then The number of opiate overdoses you witnessed since &[DATE2] cannot be greater than the number of opiate overdoses you witnessed in the past 12 months. and skip to I244.

If Q3a is equal to 1 and I241 is greater than 0 and I244 is equal to 0, then skip to instruction before I248.

If I244 is equal to 0, then skip to instruction before I322.

If I244 is equal to 1, then skip to instruction before I246.

I245. Of these [Response to I244] opiate overdoses, how many **different** people was this? For example, was it 1 person who overdosed [Response to I244] times? Or different people each time?

__ __ People

If I245 is greater than I244 then The number of people that you witnessed overdose should be less than the number of times you witnessed an overdose. and skip to instruction before I244.

I246. **Since [DATE2]** (that is, in the last [DAYS2] days), how many times have you administered naloxone to someone for an opiate overdose? This means you personally gave an injection or squirted it up their nose.

__ __ __ Times

If 1 is equal to 1, then skip to instruction before I248.

I247. You reported that you have not witnessed an opiate overdose. Is this correct? (Choose one)

0 No, that is not correct. I think I HAVE witnessed an opiate overdose since &[DATE2].

1 Yes, that is correct. I have NOT witnessed an opiate overdose since &[DATE2].

If Q3a is equal to 1 and I247 is equal to 0 then skip to instruction before I241.
If I247 is equal to 0 then skip to instruction before I244.
If 1 is equal to 1, then skip to instruction before I322.

Think about the **MOST RECENT** time you witnessed an opiate overdose.

I248. When did this opiate overdose happen?
(*If you can't remember the exact date, make your best guess of month and year.*)

__ __ / __ __ / __ __ __ __ mm / dd / yyyy

If I248 is less than DATE2N and Q3a is not equal to 1 and I244 is greater than 0 then This date must be after &[DATE2], (that is, in the last &[DAYS2] days). and skip to I248.
If I248 is greater than DATE2N and I244 is equal to 0 then You reported not having witnessed an overdose since &[DATE2]. and skip to instruction before I244.
If I248 is greater than VISDATE - 365 and I243 is equal to 0 then You reported not having witnessed an overdose in the last 12 months. and skip to I243.
If NETSIZE is less than 1, then skip to instruction before I250.

I249. Was this person who had an opiate overdose [Response to G159]?

1 Yes
0 No

If I249 is equal to 1, then skip to instruction before I251.
If NETSIZE is less than 2, then skip to instruction before I251.

I250. Was this person who had an opiate overdose [Response to G184]?

1 Yes
0 No

If I249 is equal to 1 or I250 is equal to 1, then skip to instruction before I252.
If NETSIZE is less than 3, then skip to instruction before I252.

I251. Was this person who had an opiate overdose [Response to G209]?

1 Yes
0 No

If I249 is equal to 1 or I250 is equal to 1 or I251 is equal to 1, then skip to I257.

I252. What was your relationship to the person who had an opiate overdose? (Choose one)

0 Friend
1 Family member
2 Sex partner
3 Stranger
4 Other (specify)

If I252 is not equal to 4, then skip to I254.

I253. Please specify, what was your relationship to the person who had an opiate overdose?

__ __

I254. What was the gender of this person who had an opiate overdose? (Choose one)

0 Male
1 Female
2 Transfemale
3 Transmale
4 Other
7 Don't Know

I255. What was the race/ ethnicity of this person who had an opiate overdose? (Choose one)

0 African American
1 Asian or Pacific Islander
2 Caucasian
3 Hispanic/ Latino
4 Mixed race/ ethnicity
5 Other
7 Don't Know

I256. What was their approximate age? __ __ Years

I257. How many people witnessed the opiate overdose? Include yourself as a witness.

__ __ __ People
997 Don't Know

I258. Where did this opiate overdose happen?
(Choose one) (Choose one)

00 Home/where I am staying right now
01 Someone else's place
02 Shooting gallery
04 Abandoned building
05 SRO or hotel room
06 Street, schoolyard, parking lot or other open area
07 Bar, restaurant, store or other public building
08 Car
09 Jail
10 Other (specify)

If I258 is not equal to 11, then skip to I260.

I259. Please specify, what location did this opiate overdose happen at?

__ __

I260. What **opiates** did this person use when they overdosed?
(Check all that apply) (Check all that apply)

__ Heroin
__ Oxymorphone (Opana)
__ Morphine (MSContin, Kadian, Embeda, Avinza)
__ Hydrocodone (Vicodin, Lorcet, Lortab, Norco, Zohydro)
__ Fentanyl (Duragesic)
__ Hydromorphone (Dilaudid, Palladone)
__ Oxycodone (Percocet, OxyContin, Roxicodone, Percodan)
__ Methadone (Dolophine)
__ Buprenorphine (Suboxone, Subutex)
__ Propoxyphene (Darvocet)
__ Meperidine (Demerol)
__ Codeine (Tylenol with codeine, TyCo, Tylenol #3)
__ Other
__ Don't Know

If I260M is not equal to 1, then skip to I262.

I261. Please specify, what other opiate did this person use when they overdosed?

__ __

I262. How did this person use those opiates when they overdosed?
(Check all that apply) (Check all that apply)

__ Injected
__ Oral (swallowed)
__ Smoked
__ Sniffed
__ Patch
__ Suppository ("booty bump")
__ Don't Know

I263. Did this person use any other non-opiate drugs or alcohol when they overdosed?

1 Yes
0 No
7 Don't Know

If I263 is not equal to 1, then skip to instruction before I265.

I264. What other **non-opiate drugs** did this person use when they overdosed?
(Check all that apply) (Check all that apply)

__ Alcohol
__ Benzodiazepines (Valium, Ativan, Klonopin, etc)
__ Crack Cocaine
__ Powder Cocaine
__ Methamphetamines/amphetamine (Crystal, Speed, Tina)
__ Marijuana
__ Ecstasy (E, X, MDMA)
__ GHB (G, GBL)
__ Hallucinogens (LSD, mushrooms, Peyote, or Mescaline)
__ PCP (Angel Dust, wet, wicky sticks)
__ Poppers (Amyl Nitrate)
__ Rohypnol (Roofies)
__ Ketamine (Special K)
__ Other Tranquilizers, Barbiturates
__ Viagra or similar drugs (Levitra, Cialis)
__ Bath salts
__ Other
__ Don't Know

If I264Q is not equal to 1, then skip to I266.

I265. Please specify, what other drug did this person use when they overdosed?

__ __

I266. During this person's opiate overdose, who if anyone, was the first person to check to **see if they could be woken up**? (Choose one)

0 Nobody
1 Me
2 Another Witness
3 An emergency responder (paramedic, police, or other)
7 Don't Know

I267. During this person's opiate overdose, who if anyone, was the first person to **call 911 / ambulance**? (Choose one)

0 Nobody
1 Me
2 Another witness
7 Don't Know

I268. During this person's opiate overdose, who if anyone, was the first person to **do rescue breathing**? (Choose one)

0 Nobody
1 Me
2 Another witness
3 An emergency responder (paramedic, police, or other)
7 Don't Know

I269. During this person's opiate overdose, who if anyone, was the first person to **do chest compressions** (pushing on the chest in case their heart stopped)? (Choose one)

0 Nobody
1 Me
2 Another witness
3 An emergency responder (paramedic, police, or other)
7 Don't Know

I270. During this person's opiate overdose, who if anyone, was the first person to **give naloxone**? (Choose one)

0 Nobody
1 Me
2 Another witness
3 An emergency responder (paramedic, police, or other)
7 Don't Know

If I270 is equal to 0 or I270 is equal to 7, then skip to instruction before I272.

I271. How was the naloxone given during this person's opiate overdose? (Check all that apply)

__ Injected
__ Sprayed up the nose
__ Don't Know

If I270 is not equal to 1, then skip to instruction before I277.

I272. Did you have any trouble using the naloxone?

1 Yes
0 No
7 Don't Know

If I272 is not equal to 1, then skip to instruction before I275.
If I271A is not equal to 1, then skip to instruction before I274.

I273. What trouble did you have using the naloxone?
(Check all that apply) (Check all that apply)

__ Hard time finding the naloxone
__ Hard time putting the kit together
__ Hard time injecting naloxone into the person
__ Other (specify)

If I271B is not equal to 1, then skip to instruction before I275.

I274. What trouble did you have using the naloxone?
(Check all that apply) (Check all that apply)

__ Hard time finding the naloxone
__ Hard time putting the kit together
__ Hard time spraying naloxone into their nose
__ Other (specify)

If I273F is not equal to 1 and I274F is not equal to 1, then skip to I276.

I275. Please specify, what other trouble did you have using the naloxone?

__ __
__ __
__ __
__ __
__ __
__ __ __ __ __ __ __ __ __ __ __ __ __ __ __ __ __ __ __ __

I276. Whose naloxone was it? (Choose one)

0 Mine
1 The person who was overdosing
2 Another Witness
3 An emergency responder (paramedic, police, or other)
7 Don't Know

If I270 is equal to 0 or I270 is equal to 7, then skip to I279.

I277. How many doses were given to the person during this person's opiate overdose?

__ __ Doses
97 Don't Know

I278. How long did it take for the naloxone to work (that is, for the person to wake up or start breathing)? (Choose one)

0 Less than 1 min
1 1-5 mins
2 >5 mins
3 It didn't work
7 Don't Know

I279. During this person's opiate overdose, did you or someone else inject the person with any of the following?
(check all that apply) (Check all that apply)

__ Milk
__ Crack/ powder cocaine
__ Methamphetamines/amphetamine (Crystal, Speed, Tina)
__ Water or salt water
__ Other (specify)
__ None of these
__ Don't Know

If (I279A is equal to 1 or I279B is equal to 1 or I279C is equal to 1 or I279D is equal to 1 or I279E is equal to 1) and I279F is equal to 1 then You cannot have injected something AND nothing. and skip to I279.
If I279E is not equal to 1, then skip to I281.

I280. Please specify, what else did you or someone else inject the person with?

__ __

I281. Did an ambulance come during this person's opiate overdose?

1 Yes
0 No
7 Don't Know

If I281 is not equal to 1, then skip to I283.

I282. Did the ambulance take this person to the hospital?

1 Yes
0 No
7 Don't Know

I283. Was this person admitted to the hospital - that means they got a bed in the hospital and stayed at least overnight?

1 Yes
0 No
7 Don't Know

I284. Did this person survive the opiate overdose?

1 Yes
0 No
7 Don't Know

If Q3a is equal to 1 and I244 is equal to 0 or I244 is less than 2, then skip to instruction before J327.

Think about the **SECOND MOST RECENT** time you witnessed an opiate overdose.

I285. When did this opiate overdose happen?
(*If you can't remember the exact date, make your best guess of month and year.*)
__ __ / __ __ / __ __ __ __ mm / dd / yyyy

If I285 is less than DATE2N and Q3a is not equal to 1 and I244 is greater than 0 then This date must be after &[DATE2], (that is, in the last &[DAYS2] days). and skip to I285.
If NETSIZE is less than 1, then skip to instruction before I287.

I286. Was this person who had an opiate overdose [Response to G159]?
1 Yes
0 No

If I286 is equal to 1, then skip to instruction before I288.
If NETSIZE is less than 2, then skip to instruction before I288.

I287. Was this person who had an opiate overdose [Response to G184]?
1 Yes
0 No

If I286 is equal to 1 or I287 is equal to 1, then skip to instruction before I289.
If NETSIZE is less than 3, then skip to instruction before I289.

I288. Was this person who had an opiate overdose [Response to G209]?
1 Yes
0 No

If I286 is equal to 1 or I287 is equal to 1 or I288 is equal to 1, then skip to I294.

I289. What was your relationship to the person who had an opiate overdose? (Choose one)
0 Friend
1 Family member
2 Sex partner
3 Stranger
4 Other (specify)

If I289 is not equal to 4, then skip to I291.

I290. Please specify, what was your relationship to the person who had an opiate overdose?
__ __

I291. What was the gender of this person who had an opiate overdose? (Choose one)
0 Male
1 Female
2 Transfemale
3 Transmale
4 Other
7 Don't Know

I292. What was the race/ ethnicity of this person who had an opiate overdose? (Choose one)
0 African American
1 Asian or Pacific Islander
2 Caucasian
3 Hispanic/ Latino
4 Mixed race/ ethnicity
5 Other
7 Don't Know

I293. What was their approximate age? __ __ Years

I294. How many people witnessed the opiate overdose? Include yourself as a witness.
__ __ __ People
997 Don't Know

I295. Where did this opiate overdose happen?
(Choose one) (Choose one)

00	Home/where I am staying right now
01	Someone else's place
02	Shooting gallery
04	Abandoned building
05	SRO or hotel room
06	Street, schoolyard, parking lot or other open area
07	Bar, restaurant, store or other public building
08	Car
09	Jail
10	Other (specify)

If I295 is not equal to 11, then skip to I297.

I296. Please specify, what location did this opiate overdose happen at?

__ __

I297. What **opiates** did this person use when they overdosed?
(Check all that apply) (Check all that apply)

__	Heroin
__	Oxymorphone (Opana)
__	Morphine (MSContin, Kadian, Embeda, Avinza)
__	Hydrocodone (Vicodin, Lorcet, Lortab, Norco, Zohydro)
__	Fentanyl (Duragesic)
__	Hydromorphone (Dilaudid, Palladone)
__	Oxycodone (Percocet, OxyContin, Roxicodone, Percodan)
__	Methadone (Dolophine)
__	Buprenorphine (Suboxone, Subutex)
__	Propoxyphene (Darvocet)
__	Meperidine (Demerol)
__	Codeine (Tylenol with codeine, TyCo, Tylenol #3)
__	Other
__	Don't Know

If I297M is not equal to 1, then skip to I299.

I298. Please specify, what other opiate did this person use when they overdosed?

__ __

I299. How did this person use those opiates when they overdosed?
(Check all that apply) (Check all that apply)

__	Injected
__	Oral (swallowed)
__	Smoked
__	Sniffed
__	Patch
__	Suppository ("booty bump")
__	Don't Know

I300. Did this person use any other non-opiate drugs or alcohol when they overdosed?

1	Yes
0	No
7	Don't Know

If I300 is not equal to 1, then skip to instruction before I302.

I301. What other **non-opiate drugs** did this person use when they overdosed?
(Check all that apply) (Check all that apply)

__ Alcohol
__ Benzodiazepines (Valium, Ativan, Klonopin, etc)
__ Crack Cocaine
__ Powder Cocaine
__ Methamphetamines/amphetamine (Crystal, Speed, Tina)
__ Marijuana
__ Ecstasy (E, X, MDMA)
__ GHB (G, GBL)
__ Hallucinogens (LSD, mushrooms, Peyote, or Mescaline)
__ PCP (Angel Dust, wet, wicky sticks)
__ Poppers (Amyl Nitrate)
__ Rohypnol (Roofies)
__ Ketamine (Special K)
__ Other Tranquilizers, Barbiturates
__ Viagra or similar drugs (Levitra, Cialis)
__ Bath salts
__ Other
__ Don't Know

If I301Q is not equal to 1, then skip to I303.

I302. Please specify, what other drug did this person use when they overdosed?

__ __

I303. During this person's opiate overdose, who if anyone, was the first person to check to **see if they could be woken up**? (Choose one)

0 Nobody
1 Me
2 Another Witness
3 An emergency responder (paramedic, police, or other)
7 Don't Know

I304. During this person's opiate overdose, who if anyone, was the first person to **call 911 / ambulance**? (Choose one)

0 Nobody
1 Me
2 Another witness
7 Don't Know

I305. During this person's opiate overdose, who if anyone, was the first person to **do rescue breathing**? (Choose one)

0 Nobody
1 Me
2 Another witness
3 An emergency responder (paramedic, police, or other)
7 Don't Know

I306. During this person's opiate overdose, who if anyone, was the first person to **do chest compressions** (pushing on the chest in case their heart stopped)? (Choose one)

0 Nobody
1 Me
2 Another witness
3 An emergency responder (paramedic, police, or other)
7 Don't Know

I307. During this person's opiate overdose, who if anyone, was the first person to **give naloxone**? (Choose one)

0 Nobody
1 Me
2 Another witness
3 An emergency responder (paramedic, police, or other)
7 Don't Know

If I307 is equal to 0 or I307 is equal to 7, then skip to instruction before I309.

I308. How was the naloxone given during this person's opiate overdose? (Check all that apply)

__ Injected
__ Sprayed up the nose
__ Don't Know

If I307 is not equal to 1, then skip to instruction before I314.

I309. Did you have any trouble using the naloxone?

1 Yes
0 No
7 Don't Know

If I309 is not equal to 1, then skip to instruction before I312.
If I308A is not equal to 1, then skip to instruction before I311.

I310. What trouble did you have using the naloxone?
(Check all that apply) (Check all that apply)

__ Hard time finding the naloxone
__ Hard time putting the kit together
__ Hard time injecting naloxone into the person
__ Other (specify)

If I308B is not equal to 1, then skip to instruction before I312.

I311. What trouble did you have using the naloxone?
(Check all that apply) (Check all that apply)

__ Hard time finding the naloxone
__ Hard time putting the kit together
__ Hard time spraying naloxone into their nose
__ Other (specify)

If I310F is not equal to 1 and I311F is not equal to 1, then skip to I313.

I312. Please specify, what other trouble did you have using the naloxone?

__ __
__ __
__ __
__ __
__ __
__ __ __ __ __ __ __ __ __ __ __ __ __ __ __ __ __ __ __ __

I313. Whose naloxone was it? (Choose one)

0 Mine
1 The person who was overdosing
2 Another Witness
3 An emergency responder (paramedic, police, or other)
7 Don't Know

If I307 is equal to 0 or I307 is equal to 7, then skip to I316.

I314. How many doses were given to the person during this person's opiate overdose?

__ __ Doses
97 Don't Know

I315. How long did it take for the naloxone to work (that is, for the person to wake up or start breathing)? (Choose one)

0 Less than 1 min
1 1-5 mins
2 >5 mins
3 It didn't work
7 Don't Know

I316. During this person's opiate overdose, did you or someone else inject the person with any of the following?
(check all that apply) (Check all that apply)

__ Milk
__ Crack/ powder cocaine
__ Methamphetamines/amphetamine (Crystal, Speed, Tina)
__ Water or salt water
__ Other (specify)
__ None of these
__ Don't Know

If (I316A is equal to 1 or I316B is equal to 1 or I316C is equal to 1 or I316D is equal to 1 or I316E is equal to 1) and I316F is equal to 1 then You cannot have injected something AND nothing. and skip to I316.
If I316E is not equal to 1, then skip to I318.

I317. Please specify, what else did you or someone else inject the person with?

__ __

I318. Did an ambulance come during this person's opiate overdose?

1 Yes
0 No
7 Don't Know

If I318 is not equal to 1, then skip to I320.

I319. Did the ambulance take this person to the hospital?

1 Yes
0 No
7 Don't Know

I320. Was this person admitted to the hospital - that means they got a bed in the hospital and stayed at least overnight?

1 Yes
0 No
7 Don't Know

I321. Did this person survive the opiate overdose?

1 Yes
0 No
7 Don't Know

If 1 is equal to 1, then skip to instruction before J327.
If Q3a is equal to 1 and I241 is equal to 0, then skip to I323.

I322. **Since [DATE2]** (that is, in the last [DAYS2] days), you reported not witnessing any opiate overdoses. Is this correct? (Choose one)

0 No, this is not correct. I think I have witnessed an opiate overdose since &[DATE2].
1 Yes, this is correct. I have NOT witnessed an opiate overdose since &[DATE2].

If I322 is equal to 0 and Q3a is equal to 1 then skip to instruction before I241.
If I322 is equal to 0 then skip to instruction before I244.

I323. **Since [DATE2]** (that is, in the last [DAYS2] days), how many opiate overdoses have you heard about happening to people you know?

__ __ __ Overdoses

I324. **Since [DATE2]** (that is, in the last [DAYS2] days), why do you think you haven't witnessed an opiate overdose?
(Check all that apply) (Check all that apply)

__ Rarely use with other people
__ My drug using partners don't overdose
__ I'm not using
__ I'm in rehab
__ Other (specify)

If I324E is not equal to 1, then skip to I326.

I325. Please specify, why else do you think you haven't witnessed an opiate overdose?

__ __
__ __
__ __
__ __
__ __
__ __ __ __ __ __ __ __ __ __ __ __ __ __ __ __ __ __ __ __

I326. What do you think you would have done if you had witnessed an opiate overdose?
(Check all that apply) (Check all that apply)

__ Flee the scene
__ Give naloxone
__ Cause pain
__ Call 911
__ Do rescue breathing
__ Rub their chest
__ Do chest compressions
__ Give a milk shot
__ Put ice down their pants
__ Wait with person until help arrives
__ Don't Know

PERSONAL OPIATE OVERDOSE HISTORY

The following questions will ask about your personal experience with heroin or prescription opiate overdoses.

If Q3a is not equal to 1, then skip to instruction before J336.

J327. **In your lifetime**, how many opiate overdoses have you had? (If you don't recall exactly, make your best guess.)

__ __ __ Overdoses

If J327 is equal to 0 then You reported that you have never had an opiate overdose. Please confirm that you have never had an opiate overdose in your lifetime. If you have never had an opiate overdose in your lifetime, please contact study staff. and skip to J327.

J328. How old were you the first time you overdosed? If you don't recall exactly, make your best guess.

__ __ Age

If NETSIZE is less than 1, then skip to instruction before J330.

J329. Has [Response to G159] ever given you naloxone when you had an opiate overdose?

1 Yes
0 No
7 Don't Know

If NETSIZE is less than 2, then skip to instruction before J331.

J330. Has [Response to G184] ever given you naloxone when you had an opiate overdose?

1 Yes
0 No
7 Don't Know

If NETSIZE is less than 3, then skip to J332.

J331. Has [Response to G209] ever given you naloxone when you had an opiate overdose?

1 Yes
0 No
7 Don't Know

J332. Who[WITTXT], if anyone, has ever given you naloxone for an opiate overdose? *(Check all that apply)* (Check all that apply)

__ A non-medical person I knew
__ A non-medical person I didn't know
__ Police
__ Paramedics/ambulance staff
__ Emergency room staff
__ Nobody
__ Don't Know

If J332F is equal to 1 and (J332A is equal to 1 or J332B is equal to 1 or J332C is equal to 1 or J332D is equal to 1 or J332E is equal to 1) then You cannot report someone AND nobody. and skip to J332.

J333. How many times have you ever been taken to the emergency department (ER) because of an opiate overdose?

__ __ __ Times
997 Don't Know

If J333 is equal to 0 or J333 is equal to 997, then skip to instruction before J335.

J334. How many times have you ever been admitted to the hospital because of an opiate overdose - that means you got a bed in the hospital and stayed at least overnight?

__ __ __ Times
997 Don't Know

If J334 is greater than J333 then You cannot have been admitted to the hospital more times than you have been taken to the ER. and skip to J334.

Now the questions will ask about more recent opiate overdoses.

J335. **In the past 12 months**, how many opiate overdoses have you had? (If you don't recall exactly, make your best guess.)

__ __ __ Overdoses

If J335 is greater than J327 then This number cannot be greater than the number of overdoses you've had in your lifetime. and skip to J335.
If J335 is equal to 0, then skip to instruction before J350.
If B53 is equal to 0 and DATE1N is equal to DATE2N, then skip to instruction before K458.
If DATE1 is equal to DATE2, then skip to J336.

For these next questions, we are going to ask you about personal opiate overdose experinces **since [DATE2] (that is, in the last [DAYS2])**.

J336. **Since [DATE2]** (that is, in the last [DAYS2] days), how many opiate overdoses have you had? (If you don't recall exactly, make your best guess.)

__ __ __ Overdoses

If J336 is greater than J335 and Q3a is equal to 1 then This number cannot be greater than the number of overdoses you've had in the past 12 months. and skip to J336.
If J336 is equal to 0 and Q3a is equal to 1, then skip to instruction before J350.
If J336 is equal to 0, then skip to J346.
If NETSIZE is less than 1, then skip to instruction before J338.

J337. Has [Response to G159] witnessed **any** of the opiate overdoses you have had **since [DATE2]** (that is, in the last [DAYS2] days)?

1 Yes
0 No
7 Don't Know

If NETSIZE is less than 2, then skip to instruction before J339.

J338. Has [Response to G184] witnessed **any** of the opiate overdoses you have had **since [DATE2]** (that is, in the last [DAYS2] days)?

1 Yes
0 No
7 Don't Know

If NETSIZE is less than 3, then skip to J340.

J339. Has [Response to G209] witnessed **any** of the opiate overdoses you have had **since [DATE2]** (that is, in the last [DAYS2] days)?

1 Yes
0 No
7 Don't Know

J340. **Since [DATE2]** (that is, in the last [DAYS2] days) how many times has naloxone been administered to you for an opiate overdose?

__ __ Times
97 Don't Know

If J340 is equal to 0 or J340 is equal to 97, then skip to instruction before J344.
If NETSIZE is less than 1 or Q3a is equal to 1 and J329 is equal to 0, then skip to instruction before J342.
If J337 is not equal to 1, then skip to instruction before J342.

J341. Has [Response to G159] administered naloxone to you for **any** of the opiate overdoses you have had **since [DATE2]** (that is, in the last [DAYS2] days)?

1 Yes
0 No
7 Don't Know

If J340 is equal to 1 and J341 is equal to 1, then skip to instruction before J344.
If NETSIZE is less than 2 or Q3a is equal to 1 and J330 is equal to 0, then skip to instruction before J343.

If J338 is not equal to 1, then skip to instruction before J343.

J342. Has [Response to G184] administered naloxone to you for **any** of the opiate overdoses you have had **since [DATE2]** (that is, in the last [DAYS2] days)?

1 Yes
0 No
7 Don't Know

If J340 is equal to 1 and J342 is equal to 1 or J340 is equal to 2 and J341 is equal to 1 and J342 is equal to 1, then skip to instruction before J344.
If NETSIZE is less than 3 or Q3a is equal to 1 and J331 is equal to 0, then skip to instruction before J344.
If J339 is not equal to 1, then skip to instruction before J344.

J343. Has [Response to G209] administered naloxone to you for **any** of the opiate overdoses you have had **since [DATE2]** (that is, in the last [DAYS2] days)?

1 Yes
0 No
7 Don't Know

If J333 is equal to 0 and Q3a is equal to 1, then skip to instruction before J350.

J344. **Since [DATE2]** (that is, in the last [DAYS2] days), how many times have you been taken to the emergency department (ER) because of an opiate overdose?

__ __ __ Times
997 Don't Know

If J344 is greater than J333 and Q3a is equal to 1 and J344 is not equal to 997 then You cannot have been taken to the ER more times since &[DATE2] than you have ever been taken to the ER. and skip to J344.
If J344 is greater than J336 then You cannot have gone to the ER more times for an opioid overdose than the number of opioid overdoses you have had during this period of time. and skip to J344.
If J344 is equal to 0 or J334 is equal to 0, then skip to instruction before J346.

J345. **Since [DATE2]** (that is, in the last [DAYS2] days), how many times were you admitted to the hospital because of an opiate overdose - that means you got a bed in the hospital and stayed at least overnight?

__ __ __ Times
997 Don't Know

If J345 is greater than J344 and J345 is not equal to 997 then You cannot have been admitted to the hospital more times than you have been taken to the ER. and skip to J345.
If J345 is greater than J334 and Q3a is equal to 1 and J345 is not equal to 997 then You cannot have been admitted to the hospital more times since &[DATE2] than you have ever been admitted to the hospital. and skip to J345.
If 1 is equal to 1, then skip to instruction before J350.

J346. **Since [DATE2]** (that is, in the last [DAYS2] days), you reported not having any opiate overdoses. Is this correct? (Choose one)

0 No, this is not correct. I think I HAVE had an opiate overdose since &[DATE2]
1 Yes, this is correct. I have NOT had an opiate overdose since &[DATE2].

If J346 is equal to 0 then skip to instruction before J336.

J347. Since [DATE2] (that is, in the last [DAYS2]), why do you think you haven't had an opiate overdose?
(Check all that apply)
(Check all that apply)

__ Using less
__ Using same amount
__ No change in dealer
__ In treatment
__ In jail/ prison
__ Other (specify)

If J347F is not equal to 1, then skip to J349.

J348. Please specify, since [DATE2] (that is, in the last [DAYS2] days), why else haven't you had an opiate overdose?

__ __

J349. Since [DATE2] (that is, in the last [DAYS2], what do you think would have happened if you had had an opiate overdose during this time? (Check all that apply)

__ People I know would have called 911
__ People I know would have given me naloxone
__ People I know would have done rescue breathing
__ People I know would have done chest compressions
__ I probably would have died
__ Don't Know

If 1 is equal to 1, then skip to instruction before K458.

Please now think about your **MOST RECENT** opiate overdose.

J350. When did this opiate overdose happen?
(If you can't remember the exact date, make your best guess of month and year.)

__ __ / __ __ / __ __ __ __ mm / dd / yyyy

If Date(J350Y, J350M, J350D) is less than DATE2N and Q3a is not equal to 1 and J336 is greater than 0 then Please tell us only about the opiate overdoses that have occured since &[DATE2]. and skip to J350.
If J350 is greater than DATE2N and J336 is equal to 0 then You reported not having experienced an overdose since &[DATE2]. and skip to instruction before J336.
If J350 is greater than VISDATE - 365 and J335 is equal to 0 then You reported not having experienced an overdose in the last 12 months. and skip to J335.

J351. What **opiates** were you using **at the time of this opiate overdose**?
(Check all that apply) (Check all that apply)

__ Heroin
__ Oxymorphone (Opana)
__ Morphine (MSContin, Kadian, Embeda, Avinza)
__ Hydrocodone (Vicodin, Lorcet, Lortab, Norco, Zohydro)
__ Fentanyl (Duragesic)
__ Hydromorphone (Dilaudid, Palladone)
__ Oxycodone (Percocet, OxyContin, Roxicodone, Percodan)
__ Methadone (Dolophine)
__ Buprenorphine (Suboxone, Subutex)
__ Propoxyphene (Darvocet)
__ Meperidine (Demerol)
__ Codeine (Tylenol with codeine, TyCo, Tylenol #3)
__ Other
__ Don't Know

If J351M is not equal to 1, then skip to J353.

J352. Please specify, what other opiate(s) were you using **at the time of this opiate overdose**?

__ __

J353. How did you use those opiate(s) at the time of this opiate overdose? (*Check all that apply*) (Check all that apply)

__ Injected
__ Oral (swallowed)
__ Smoked
__ Sniffed
__ Patch
__ Suppository ("booty bump")
__ Don't Know

J354. Did you use **non-opiate drugs or alcohol at the time of this opiate overdose**?

1 Yes
0 No
7 Don't Know

If J354 is not equal to 1, then skip to J356.

J355. What other non-opiate drugs were you using **at the time of this opiate overdose**? *(Check all that apply)* (Check all that apply)

__ Alcohol
__ Benzodiazepines (Valium, Ativan, Klonopin, etc)
__ Crack Cocaine
__ Powder Cocaine
__ Methamphetamines/amphetamine (Crystal, Speed, Tina)
__ Marijuana
__ Ecstasy (E, X, MDMA)
__ GHB (G, GBL)
__ Hallucinogens (LSD, mushrooms, Peyote, or Mescaline)
__ PCP (Angel Dust, wet, wicky sticks)
__ Poppers (Amyl Nitrate)
__ Rohypnol (Roofies)
__ Ketamine (Special K)
__ Other Tranquilizers, Barbiturates
__ Viagra or similar drugs (Levitra, Cialis)
__ Bath salts
__ Other
__ Don't Know

J356. Where were you at the time of this opiate overdose? (Choose one)

00	Bayview / Hunters Point
01	Bernal Heights
02	Castro
03	Excelsior
04	Haight Ashbury / Lower Haight
05	Mission
06	Nob Hill
07	North Beach
08	Potrero Hill / Mission Bay
09	Richmond (Inner and Outer)
10	Russian Hill / Pacific Heights
11	SOMA
12	Sunset (Inner and Outer)
13	Tenderloin
14	Twin Peaks
15	Union Square / Chinatown
16	Visitacion Valley
17	Western Addition
18	Other San Francisco neighborhood (specify)
19	East Bay (specify)
20	Other (specify)

If J356 is less than 17 or J356 is equal to 97, then skip to J358.

J357. Please specify, where were you at the time of this opiate overdose?

— —

J358. How many people witnessed your last opiate overdose?

__ __	People
97	Don't Know

If J358 is equal to 0, then skip to instruction before J364.
If NETSIZE is less than 1 or J337 is not equal to 1 and Date(J350Y, J350M, J350D) is greater than DATE2N, then skip to instruction before J360.

J359. Did [Response to G159] witness this opiate overdose?

1	Yes
0	No
7	Don't Know

If J358 is equal to 1 and J359 is equal to 1, then skip to instruction before J364.
If NETSIZE is less than 2 or J338 is not equal to 1 and Date(J350Y, J350M, J350D) is greater than DATE2N, then skip to instruction before J361.

J360. Did [Response to G184] witness this opiate overdose?

1	Yes
0	No
7	Don't Know

If J358 is equal to 1 and J360 is equal to 1 or J358 is equal to 2 and J359 is equal to 1 and J360 is equal to 1, then skip to instruction before J364.
If NETSIZE is less than 3 or J339 is not equal to 1 and Date(J350Y, J350M, J350D) is greater than DATE2N, then skip to instruction before J362.

J361. Did [Response to G209] witness this opiate overdose?

1	Yes
0	No
7	Don't Know

If J358 is equal to 1 and J361 is equal to 1 or J358 is equal to 2 and J359 is equal to 1 and J361 is equal to 1 or J358 is equal to 2 and J360 is equal to 1 and J361 is equal to 1 or J358 is equal to 3 and J359 is equal to 1 and J360 is equal to 1 and J361 is equal to 1, then skip to instruction before J364.
If J358 is equal to ODPELSA1, then skip to instruction before J364.

J362. Who[ELSTXTA1] witnessed this opiate overdose?
(Check all that apply) (Check all that apply)

__	Friend
__	Family member
__	Sex partner
__	Stranger
__	Other (specify)
__	Nobody
__	Don't Know

If J362F is equal to 1 and (J362A is equal to 1 or J362B is equal to 1 or J362C is equal to 1 or J362D is equal to 1 or J362E is equal to 1) then You cannot report somebody AND nobody. and skip to J362.
If J362F is equal to 1 and J358 is greater than ODPELSA1 then You reported that at least one other person saw this opiate overdose. and skip to J362.
If J362E is not equal to 1, then skip to instruction before J364.

J363. Please specify, what was your relationship to the person who witnessed your opiate overdose?

__ __

If J358 is greater than 0, then skip to J369.
If NETSIZE is less than 1, then skip to instruction before J365.
If J359 is equal to 1, then skip to instruction before J365.

J364. Did [Response to G159] know you were using opiates and could have checked on you at the time of this opiate overdose?

1	Yes
0	No
7	Don't Know

If NETSIZE is less than 2, then skip to instruction before J366.
If J360 is equal to 1, then skip to instruction before J366.

J365. Did [Response to G184] know you were using opiates and could have checked on you at the time of this opiate overdose?

1	Yes
0	No
7	Don't Know

If NETSIZE is less than 3, then skip to J367.
If J361 is equal to 1, then skip to J367.

J366. Did [Response to G209] know you were using opiates and could have checked on you at the time of this opiate overdose?

1	Yes
0	No
7	Don't Know

J367. Who[ELSTXTB1], if anyone, that didn't witness this opiate overdose knew you were using opiates and could have checked on you at the time of this opiate overdose?
(Check all that apply) (Check all that apply)

__	Friend
__	Family member
__	Sex partner
__	Stranger
__	Other (specify)
__	Nobody
__	Don't Know

If J367F is equal to 1 and (J367A is equal to 1 or J367B is equal to 1 or J367C is equal to 1 or J367D is equal to 1 or J367E is equal to 1) then You cannot report somebody AND nobody. and skip to J367.
If J367E is not equal to 1, then skip to J369.

J368. Please specify, what was your relationship to the person who witnessed you having an opiate overdose?

__ __

J369. Did someone **call 911** or call for medical help during that opiate overdose?

1 Yes
0 No
7 Don't Know

If J340 is equal to 0 and Date(J350Y, J350M, J350D) is greater than DATE2N, then skip to J375.

J370. Did someone **give you naloxone** to treat that opiate overdose?

1 Yes
0 No
7 Don't Know

If J370 is equal to 0 or J370 is equal to 7, then skip to J375.
If NETSIZE is less than 1 or J341 is equal to 0 or J329 is equal to 0 and Q3a is equal to 1, then skip to instruction before J372.
If J359 is not equal to 1, then skip to instruction before J372.

J371. Did [Response to G159] give you naloxone to treat that opiate overdose?

1 Yes
0 No
7 Don't Know

If NETSIZE is less than 2 or J342 is equal to 0 or J330 is equal to 0 and Q3a is equal to 1, then skip to instruction before J373.
If J360 is not equal to 1, then skip to instruction before J373.

J372. Did [Response to G184] give you naloxone to treat that opiate overdose?

1 Yes
0 No
7 Don't Know

If NETSIZE is less than 3 or J343 is equal to 0 or J331 is equal to 0 and Q3a is equal to 1, then skip to J374.
If J361 is not equal to 1, then skip to J374.

J373. Did [Response to G209] give you naloxone to treat that opiate overdose?

1 Yes
0 No
7 Don't Know

J374. Who[ELSTXTC1] gave you naloxone to treat that opiate overdose?
(Check all that apply) (Check all that apply)

__ Another non-medical person who I knew
__ A non-medical person I didn't know
__ Paramedics/ ambulance staff
__ Emergency room staff
__ Police
__ Nobody
__ Don't Know

If J374F is equal to 1 and (J374A is equal to 1 or J374B is equal to 1 or J374C is equal to 1 or J374D is equal to 1 or J374E is equal to 1) then You cannot report somebody AND nobody. and skip to J374.
If J374F is equal to 1 and ODPELSC1 is equal to 0 then You reported that at least one person administered naloxone to you during this opiate overdose. and skip to J374.

J375. Did someone **perform rescue breathing** during that opiate overdose?

1 Yes
0 No
7 Don't Know

J376. Did someone **do chest compressions** (pushing on your chest to act like your heart is beating) during that opiate overdose?

1 Yes
0 No
7 Don't Know

If J344 is equal to 0 or Q3a is equal to 1 and J333 is equal to 0, then skip to J380.

J377. Were you **taken to the emergency room** during that opiate overdose? (Choose one)

0 No
1 Yes, by ambulance
2 Yes, by people who were with me
7 Don't Know

If J377 is equal to 0 or J377 is greater than 2, then skip to J380.

J378. Which emergency room were you taken to during that opiate overdose? (Choose one)

00 San Francisco General Hospital
01 UCSF Hospital
02 CPMC Pacific
03 CPMC Davies
04 St Luke's
05 St Francis
09 ER outside of San Francisco
10 Other (specify)
97 Don't Know

If J334 is not greater than 0 and Q3a is equal to 1 or J345 is not greater than 0, then skip to J380.

J379. Were you admitted to the hospital - that means you got a bed in the hospital and stayed at least overnight?

1 Yes
0 No
7 Don't Know

J380. Were there any problems from that opiate overdose?
(Check all that apply) (Check all that apply)

__ I was arrested
__ I had to stay in the hospital for more than 1 night
__ I had an injury from the overdose (like a fall, nerve damage, or any other physical injury)
__ I had problems with my lungs (like pneumonia or more difficulty breathing)
__ I had some other medical problem from the overdose
__ I felt emotionally traumatized by the overdose
__ No problem
__ Other (specify)
__ Don't Know

If J380G is equal to 1 and (J380H is equal to 1 or J380A is equal to 1 or J380B is equal to 1 or J380C is equal to 1 or J380D is equal to 1 or J380E is equal to 1 or J380F is equal to 1) then You cannot select a problem AND "no problem". and skip to J380.
If J380H is not equal to 1, then skip to instruction before J382.

J381. Please specify, what other problems did you have from that opiate overdose?

__ __

You said that this overdose happened around [ODDTXT1].

J382. Had you been released from jail or prison between [ODD3TXT1] and [ODDTXT1]?

1 Yes
0 No
7 Don't Know

If Date(J350Y, J350M, J350D) is greater than DATE1N and Q3a is equal to 1, then skip to instruction before J402.

J383. Were you receiving any drug treatment between [ODD3TXT1] and [ODDTXT1]?

1 Yes
0 No
7 Don't Know

If J383 is not equal to 1, then skip to instruction before J385.

J384. What treatment program were you in?
(Check all that apply) (Check all that apply)

__ 12-step program
__ Residential drug or alcohol treatment
__ Outpatient substance use counseling
__ Methadone maintenance treatment
__ Buprenorphine (suboxone) treatment
__ Detoxification (a few days of intense care)
__ Vivitrol (naltrexone) treatment
__ Other (specify)

If J384H is not equal to 1, then skip to instruction before J386.

J385. Please specify, what treatment program were you in?

__ __

If J384A is not equal to 1, then skip to instruction before J388.

J386. Did you stop this 12-step program before your opiate overdose on [ODDTXT1]?

1 Yes
0 No

If J386 is not equal to 1, then skip to instruction before J388.

J387. How many days prior to the opiate overdose on [ODDTXT1] did you stop this 12-step program? (Choose one)

0 0 - 6 days
1 7 - 13 days
2 14 - 20 days
3 21 - 27 days
4 28 or more days

If J384B is not equal to 1, then skip to instruction before J390.

J388. Did you stop residential drug or alcohol treatment before your opiate overdose on [ODDTXT1]?

1 Yes
0 No

If J388 is not equal to 1, then skip to instruction before J390.

J389. How many days prior to the opiate overdose on [ODDTXT1] did you stop this residential drug or alcohol treatment? (Choose one)

0 0 - 6 days
1 7 - 13 days
2 14 - 20 days
3 21 - 27 days
4 28 or more days

If J384C is not equal to 1, then skip to instruction before J392.

J390. Did you stop outpatient substance use counseling before your opiate overdose on [ODDTXT1]?

1 Yes
0 No

If J390 is not equal to 1, then skip to instruction before J392.

J391. How many days prior to the opiate overdose on [ODDTXT1] did you stop outpatient substance use counseling? (Choose one)

0 0 - 6 days
1 7 - 13 days
2 14 - 20 days
3 21 - 27 days
4 28 or more days

If J384D is not equal to 1, then skip to instruction before J394.

J392. Did you stop methadone maintenance treatment before your opiate overdose on [ODDTXT1]?

1 Yes
0 No

If J392 is not equal to 1, then skip to instruction before J394.

J393. How many days prior to the opiate overdose on [ODDTXT1] did you stop methadone maintenance treatment? (Choose one)

0 0 - 6 days
1 7 - 13 days
2 14 - 20 days
3 21 - 27 days
4 28 or more days

If J384E is not equal to 1, then skip to instruction before J396.

J394. Did you stop buprenorphine (suboxone) treatment before your opiate overdose on [ODDTXT1]?

1 Yes
0 No

If J394 is not equal to 1, then skip to instruction before J396.

J395. How many days prior to the opiate overdose on [ODDTXT1] did you stop buprenorphine (suboxone) treatment? (Choose one)

0 0 - 6 days
1 7 - 13 days
2 14 - 20 days
3 21 - 27 days
4 28 or more days

If J384F is not equal to 1, then skip to instruction before J398.

J396. Did you stop detoxification (at least a few days of intense care) before your opiate overdose on [ODDTXT1]?

1 Yes
0 No

If J396 is not equal to 1, then skip to instruction before J398.

J397. How many days prior to the opiate overdose on [ODDTXT1] did you stop detoxification (a few days of intense care)? (Choose one)

0 0 - 6 days
1 7 - 13 days
2 14 - 20 days
3 21 - 27 days
4 28 or more days

If J384G is not equal to 1, then skip to instruction before J400.

J398. Did you stop vivitrol (naltrexone) treatment before your opiate overdose on [ODDTXT1]?

1 Yes
0 No

If J398 is not equal to 1, then skip to instruction before J400.

J399. How many days prior to the opiate overdose on [ODDTXT1] did you stop vivitrol (naltrexone) treatment? (Choose one)

0 0 - 6 days
1 7 - 13 days
2 14 - 20 days
3 21 - 27 days
4 28 or more days

If J384H is not equal to 1, then skip to instruction before J401.

J400. Did you stop [Response to J385] before your opiate overdose on [ODDTXT1]?

1 Yes
0 No

If J400 is not equal to 1, then skip to instruction before J402.

J401. How many days prior to the opiate overdose on [ODDTXT1] did you stop [Response to J385]? (Choose one)

0	0 - 6 days
1	7 - 13 days
2	14 - 20 days
3	21 - 27 days
4	28 or more days

If J336 is less than 2, then skip to instruction before K458.

Please now think about your **SECOND MOST RECENT** opiate overdose.

J402. When did this opiate overdose happen?
(If you can't remember the exact date, make your best guess of month and year.)

__ __ / __ __ / __ __ __ __ mm / dd / yyyy

If Date(J402Y, J402M, J402D) is less than DATE2N and Q3a is not equal to 1 and J336 is greater than 0 then Please tell us only about the opiate overdoses that have occured since &[DATE2]. and skip to instruction before J402.

J403. What **opiates** were you using **at the time of this opiate overdose**?
(Check all that apply) (Check all that apply)

__ Heroin
__ Oxymorphone (Opana)
__ Morphine (MSContin, Kadian, Embeda, Avinza)
__ Hydrocodone (Vicodin, Lorcet, Lortab, Norco, Zohydro)
__ Fentanyl (Duragesic)
__ Hydromorphone (Dilaudid, Palladone)
__ Oxycodone (Percocet, OxyContin, Roxicodone, Percodan)
__ Methadone (Dolophine)
__ Buprenorphine (Suboxone, Subutex)
__ Propoxyphene (Darvocet)
__ Meperidine (Demerol)
__ Codeine (Tylenol with codeine, TyCo, Tylenol #3)
__ Other
__ Don't Know

If J403M is not equal to 1, then skip to J405.

J404. Please specify, what other opiate(s) were you using **at the time of this opiate overdose**?

__ __

J405. How did you use those opiate(s) at the time of this opiate overdose?
(*Check all that apply*) (Check all that apply)

__ Injected
__ Oral (swallowed)
__ Smoked
__ Sniffed
__ Patch
__ Suppository ("booty bump")
__ Don't Know

J406. Did you use **non-opiate drugs or alcohol at the time of this opiate overdose**?

1	Yes
0	No
7	Don't Know

If J406 is not equal to 1, then skip to J408.

J407. What other non-opiate drugs were you using **at the time of this opiate overdose**? *(Check all that apply)* (Check all that apply)

__	Alcohol
__	Benzodiazepines (Valium, Ativan, Klonopin, etc)
__	Crack Cocaine
__	Powder Cocaine
__	Methamphetamines/amphetamine (Crystal, Speed, Tina)
__	Marijuana
__	Ecstasy (E, X, MDMA)
__	GHB (G, GBL)
__	Hallucinogens (LSD, mushrooms, Peyote, or Mescaline)
__	PCP (Angel Dust, wet, wicky sticks)
__	Poppers (Amyl Nitrate)
__	Rohypnol (Roofies)
__	Ketamine (Special K)
__	Other Tranquilizers, Barbiturates
__	Viagra or similar drugs (Levitra, Cialis)
__	Bath salts
__	Other
__	Don't Know

J408. Where were you at the time of this opiate overdose? (Choose one)

00	Bayview / Hunters Point
01	Bernal Heights
02	Castro
03	Excelsior
04	Haight Ashbury / Lower Haight
05	Mission
06	Nob Hill
07	North Beach
08	Potrero Hill / Mission Bay
09	Richmond (Inner and Outer)
10	Russian Hill / Pacific Heights
11	SOMA
12	Sunset (Inner and Outer)
13	Tenderloin
14	Twin Peaks
15	Union Square / Chinatown
16	Visitacion Valley
17	Western Addition
18	Other San Francisco neighborhood (specify)
19	East Bay (specify)
20	Other (specify)

If J408 is less than 17 or J408 is greater than 96, then skip to J410.

J409. Please specify, where were you at the time of this opiate overdose?

____

J410. How many people witnessed your last opiate overdose?

__ __	People
97	Don't Know

If J410 is equal to 0, then skip to instruction before J416.
If NETSIZE is less than 1 or J337 is not equal to 1 and Date(J402Y, J402M, J402D) is greater than DATE2N, then skip to instruction before J412.

J411. Did [Response to G159] witness this opiate overdose?

1	Yes
0	No
7	Don't Know

If J410 is equal to 1 and J411 is equal to 1, then skip to instruction before J416.

If NETSIZE is less than 2 or J338 is not equal to 1 and Date(J402Y, J402M, J402D) is greater than DATE2N, then skip to instruction before J413.

J412. Did [Response to G184] witness this opiate overdose?

1 Yes
0 No
7 Don't Know

If J410 is equal to 1 and J412 is equal to 1 or J410 is equal to 2 and J411 is equal to 1 and J412 is equal to 1, then skip to instruction before J416.
If NETSIZE is less than 3 or J339 is not equal to 1 and Date(J402Y, J402M, J402D) is greater than DATE2N, then skip to instruction before J414.

J413. Did [Response to G209] witness this opiate overdose?

1 Yes
0 No
7 Don't Know

If J410 is equal to 1 and J413 is equal to 1 or J410 is equal to 2 and J411 is equal to 1 and J413 is equal to 1 or J410 is equal to 2 and J412 is equal to 1 and J413 is equal to 1 or J410 is equal to 3 and J411 is equal to 1 and J412 is equal to 1 and J413 is equal to 1, then skip to instruction before J416.
If J410 is equal to ODPELSA2, then skip to instruction before J416.

J414. Who[ELSTXTA2] witnessed this opiate overdose?
(Check all that apply) (Check all that apply)

__ Friend
__ Family member
__ Sex partner
__ Stranger
__ Other (specify)
__ Nobody
__ Don't Know

If J414F is equal to 1 and (J414A is equal to 1 or J414B is equal to 1 or J414C is equal to 1 or J414D is equal to 1 or J414E is equal to 1) then You cannot report somebody AND nobody. and skip to J414.
If J414F is equal to 1 and J410 is greater than ODPELSA2 then You reported that at least one other person saw this opiate overdose. and skip to J414.
If J414E is not equal to 1, then skip to instruction before J416.

J415. Please specify, what was your relationship to the person who witnessed your opiate overdose?

__ __

If J410 is greater than 0, then skip to J421.
If NETSIZE is less than 1, then skip to instruction before J417.
If J411 is equal to 1, then skip to instruction before J417.

J416. Did [Response to G159] know you were using opiates and could have checked on you at the time of this opiate overdose?

1 Yes
0 No
7 Don't Know

If NETSIZE is less than 2, then skip to instruction before J418.
If J412 is equal to 1, then skip to instruction before J418.

J417. Did [Response to G184] know you were using opiates and could have checked on you at the time of this opiate overdose?

1 Yes
0 No
7 Don't Know

If NETSIZE is less than 3, then skip to J419.
If J413 is equal to 1, then skip to J419.

J418. Did [Response to G209] know you were using opiates and could have checked on you at the time of this opiate overdose?

1	Yes
0	No
7	Don't Know

J419. Who[ELSTXTB2], if anyone, that didn't witness this opiate overdose knew you were using opiates and could have checked on you at the time of this opiate overdose?
(Check all that apply) (Check all that apply)

__	Friend
__	Family member
__	Sex partner
__	Stranger
__	Other (specify)
__	Nobody
__	Don't Know

If J419F is equal to 1 and (J419A is equal to 1 or J419B is equal to 1 or J419C is equal to 1 or J419D is equal to 1 or J419E is equal to 1) then You cannot report somebody AND nobody. and skip to J419.
If J419E is not equal to 1, then skip to J421.

J420. Please specify, what was your relationship to the person who witnessed you having an opiate overdose?

____

J421. Did someone **call 911** or call for medical help during that opiate overdose?

1	Yes
0	No
7	Don't Know

If J340 is equal to 0 and Date(J402Y, J402M, J402D) is greater than DATE2N, then skip to J427.

J422. Did someone **give you naloxone** to treat that opiate overdose?

1	Yes
0	No
7	Don't Know

If J422 is equal to 0 or J422 is equal to 7, then skip to J427.
If NETSIZE is less than 1 or J341 is equal to 0 or J329 is equal to 0 and Q3a is equal to 1, then skip to instruction before J424.
If J411 is not equal to 1, then skip to instruction before J424.

J423. Did [Response to G159] give you naloxone to treat that opiate overdose?

1	Yes
0	No
7	Don't Know

If NETSIZE is less than 2 or J342 is equal to 0 or J330 is equal to 0 and Q3a is equal to 1, then skip to instruction before J425.
If J412 is not equal to 1, then skip to instruction before J425.

J424. Did [Response to G184] give you naloxone to treat that opiate overdose?

1	Yes
0	No
7	Don't Know

If NETSIZE is less than 3 or J343 is equal to 0 or J331 is equal to 0 and Q3a is equal to 1, then skip to J426.
If J413 is not equal to 1, then skip to J426.

J425. Did [Response to G209] give you naloxone to treat that opiate overdose?

1 Yes
0 No
7 Don't Know

J426. Who[ELSTXTC2] gave you naloxone to treat that opiate overdose?
(Check all that apply) (Check all that apply)

__ Another non-medical person who I knew
__ A non-medical person I didn't know
__ Paramedics/ ambulance staff
__ Emergency room staff
__ Police
__ Nobody
__ Don't Know

If J426F is equal to 1 and (J426A is equal to 1 or J426B is equal to 1 or J426C is equal to 1 or J426D is equal to 1 or J426E is equal to 1) then You cannot report somebody AND nobody. and skip to J426.
If J426F is equal to 1 and ODPELSC2 is equal to 0 then You reported that at least one person administered naloxone to you during this opiate overdose. and skip to J426.

J427. Did someone **perform rescue breathing** during that opiate overdose?

1 Yes
0 No
7 Don't Know

J428. Did someone **do chest compressions** (pushing on your chest to act like your heart is beating) during that opiate overdose?

1 Yes
0 No
7 Don't Know

If J344 is equal to 0 or Q3a is equal to 1 and J333 is equal to 0, then skip to instruction before J430.

J429. Were you **taken to the emergency room** during that opiate overdose? (Choose one)

0 No
1 Yes, by ambulance
2 Yes, by people who were with me
7 Don't Know

If J336 is less than 3, then skip to instruction before K458.

Please now think about your **THIRD MOST RECENT** opiate overdose.

J430. When did this opiate overdose happen?
(If you can't remember the exact date, make your best guess of month and year.)

__ __ / __ __ / __ __ __ __ mm / dd / yyyy

If Date(J430Y, J430M, J430D) is less than DATE2N and Q3a is not equal to 1 and J336 is greater than 0 then Please tell us only about the opiate overdoses that have occured since &[DATE2]. and skip to instruction before J430.

J431. What **opiates** were you using **at the time of this opiate overdose**?
(Check all that apply) (Check all that apply)

__ Heroin
__ Oxymorphone (Opana)
__ Morphine (MSContin, Kadian, Embeda, Avinza)
__ Hydrocodone (Vicodin, Lorcet, Lortab, Norco, Zohydro)
__ Fentanyl (Duragesic)
__ Hydromorphone (Dilaudid, Palladone)
__ Oxycodone (Percocet, OxyContin, Roxicodone, Percodan)
__ Methadone (Dolophine)
__ Buprenorphine (Suboxone, Subutex)
__ Propoxyphene (Darvocet)
__ Meperidine (Demerol)
__ Codeine (Tylenol with codeine, TyCo, Tylenol #3)
__ Other
__ Don't Know

If J431M is not equal to 1, then skip to J433.

J432. Please specify, what other opiate(s) were you using **at the time of this opiate overdose**?

__ __

J433. How did you use those opiate(s) at the time of this opiate overdose?
(*Check all that apply*) (Check all that apply)

__ Injected
__ Oral (swallowed)
__ Smoked
__ Sniffed
__ Patch
__ Suppository ("booty bump")
__ Don't Know

J434. Did you use **non-opiate drugs or alcohol at the time of this opiate overdose**?

1 Yes
0 No
7 Don't Know

If J434 is not equal to 1, then skip to J436.

J435. What other non-opiate drugs were you using **at the time of this opiate overdose**? *(Check all that apply)* (Check all that apply)

__ Alcohol
__ Benzodiazepines (Valium, Ativan, Klonopin, etc)
__ Crack Cocaine
__ Powder Cocaine
__ Methamphetamines/amphetamine (Crystal, Speed, Tina)
__ Marijuana
__ Ecstasy (E, X, MDMA)
__ GHB (G, GBL)
__ Hallucinogens (LSD, mushrooms, Peyote, or Mescaline)
__ PCP (Angel Dust, wet, wicky sticks)
__ Poppers (Amyl Nitrate)
__ Rohypnol (Roofies)
__ Ketamine (Special K)
__ Other Tranquilizers, Barbiturates
__ Viagra or similar drugs (Levitra, Cialis)
__ Bath salts
__ Other
__ Don't Know

J436. Where were you at the time of this opiate overdose? (Choose one)

00	Bayview / Hunters Point
01	Bernal Heights
02	Castro
03	Excelsior
04	Haight Ashbury / Lower Haight
05	Mission
06	Nob Hill
07	North Beach
08	Potrero Hill / Mission Bay
09	Richmond (Inner and Outer)
10	Russian Hill / Pacific Heights
11	SOMA
12	Sunset (Inner and Outer)
13	Tenderloin
14	Twin Peaks
15	Union Square / Chinatown
16	Visitacion Valley
17	Western Addition
18	Other San Francisco neighborhood (specify)
19	East Bay (specify)
20	Other (specify)

If J436 is less than 17 or J436 is greater than 96, then skip to J438.

J437. Please specify, where were you at the time of this opiate overdose?

__ __

J438. How many people witnessed your last opiate overdose? __ __ People
97 Don't Know

If J438 is equal to 0, then skip to instruction before J444.
If NETSIZE is less than 1 or J337 is not equal to 1 and Date(J430Y, J430M, J430D) is greater than DATE2N, then skip to instruction before J440.

J439. Did [Response to G159] witness this opiate overdose?
1 Yes
0 No
7 Don't Know

If J438 is equal to 1 and J439 is equal to 1, then skip to instruction before J444.
If NETSIZE is less than 2 or J338 is not equal to 1 and Date(J430Y, J430M, J430D) is greater than DATE2N, then skip to instruction before J441.

J440. Did [Response to G184] witness this opiate overdose?
1 Yes
0 No
7 Don't Know

If J438 is equal to 1 and J440 is equal to 1 or J438 is equal to 2 and J439 is equal to 1 and J440 is equal to 1, then skip to instruction before J444.
If NETSIZE is less than 3 or J339 is not equal to 1 and Date(J430Y, J430M, J430D) is greater than DATE2N, then skip to instruction before J442.

J441. Did [Response to G209] witness this opiate overdose?
1 Yes
0 No
7 Don't Know

If J438 is equal to 1 and J441 is equal to 1 or J438 is equal to 2 and J439 is equal to 1 and J441 is equal to 1 or J438 is equal to 2 and J440 is equal to 1 and J441 is equal to 1 or J438 is equal to 3 and J439 is equal to 1 and J440 is equal to 1 and J441 is equal to 1, then skip to instruction before J444.
If J438 is equal to ODPELSA3, then skip to instruction before J444.

J442. Who[ELSTXTA3] witnessed this opiate overdose?
(Check all that apply) (Check all that apply)

__ Friend
__ Family member
__ Sex partner
__ Stranger
__ Other (specify)
__ Nobody
__ Don't Know

If J442F is equal to 1 and (J442A is equal to 1 or J442B is equal to 1 or J442C is equal to 1 or J442D is equal to 1 or J442E is equal to 1) then You cannot report somebody AND nobody. and skip to J442.
If J442F is equal to 1 and J438 is greater than ODPELSA3 then You reported that at least one other person saw this opiate overdose. and skip to J442.
If J442E is not equal to 1, then skip to instruction before J444.

J443. Please specify, what was your relationship to the person who witnessed your opiate overdose?

____

If J438 is greater than 0, then skip to J449.
If NETSIZE is less than 1, then skip to instruction before J445.
If J439 is equal to 1, then skip to instruction before J445.

J444. Did [Response to G159] know you were using opiates and could have checked on you at the time of this opiate overdose?

1 Yes
0 No
7 Don't Know

If NETSIZE is less than 2, then skip to instruction before J446.
If J440 is equal to 1, then skip to instruction before J446.

J445. Did [Response to G184] know you were using opiates and could have checked on you at the time of this opiate overdose?

1 Yes
0 No
7 Don't Know

If NETSIZE is less than 3, then skip to J447.
If J441 is equal to 1, then skip to J447.

J446. Did [Response to G209] know you were using opiates and could have checked on you at the time of this opiate overdose?

1 Yes
0 No
7 Don't Know

J447. Who[ELSTXTB3], if anyone, that didn't witness this opiate overdose knew you were using opiates and could have checked on you at the time of this opiate overdose?
(Check all that apply) (Check all that apply)

__ Friend
__ Family member
__ Sex partner
__ Stranger
__ Other (specify)
__ Nobody
__ Don't Know

If J447F is equal to 1 and (J447A is equal to 1 or J447B is equal to 1 or J447C is equal to 1 or J447D is equal to 1 or J447E is equal to 1) then You cannot report somebody AND nobody. and skip to J447.
If J447E is not equal to 1, then skip to J449.

J448. Please specify, what was your relationship to the person who witnessed you having an opiate overdose?

____

J449. Did someone **call 911** or call for medical help during that opiate overdose?

1 Yes
0 No
7 Don't Know

If J340 is equal to 0 and Date(J430Y, J430M, J430D) is greater than DATE2N, then skip to J455.

J450. Did someone **give you naloxone** to treat that opiate overdose?

1 Yes
0 No
7 Don't Know

If J450 is equal to 0 or J450 is equal to 7, then skip to J455.
If NETSIZE is less than 1 or J341 is equal to 0 or J329 is equal to 0 and Q3a is equal to 1, then skip to instruction before J452.
If J439 is not equal to 1, then skip to instruction before J452.

J451. Did [Response to G159] give you naloxone to treat that opiate overdose?

1 Yes
0 No
7 Don't Know

If NETSIZE is less than 2 or J342 is equal to 0 or J330 is equal to 0 and Q3a is equal to 1, then skip to instruction before J453.
If J440 is not equal to 1, then skip to instruction before J453.

J452. Did [Response to G184] give you naloxone to treat that opiate overdose?

1 Yes
0 No
7 Don't Know

If NETSIZE is less than 3 or J343 is equal to 0 or J331 is equal to 0 and Q3a is equal to 1, then skip to J454.
If J441 is not equal to 1, then skip to J454.

J453. Did [Response to G209] give you naloxone to treat that opiate overdose?

1 Yes
0 No
7 Don't Know

J454. Who[ELSTXTC3] gave you naloxone to treat that opiate overdose?
(Check all that apply) (Check all that apply)

__ Another non-medical person who I knew
__ A non-medical person I didn't know
__ Paramedics/ ambulance staff
__ Emergency room staff
__ Police
__ Nobody
__ Don't Know

If J454F is equal to 1 and (J454A is equal to 1 or J454B is equal to 1 or J454C is equal to 1 or J454D is equal to 1 or J454E is equal to 1) then You cannot report somebody AND nobody. and skip to J454.
If J454F is equal to 1 and ODPELSC3 is equal to 0 then You reported that at least one person administered naloxone to you during this opiate overdose. and skip to J454.

J455. Did someone **perform rescue breathing** during that opiate overdose?

1 Yes
0 No
7 Don't Know

J456. Did someone **do chest compressions** (pushing on your chest to act like your heart is beating) during that opiate overdose?

1 Yes
0 No
7 Don't Know

If J344 is equal to 0 or Q3a is equal to 1 and J333 is equal to 0, then skip to instruction before K458.

J457. Were you **taken to the emergency room** during that opiate overdose? (Choose one)

0 No
1 Yes, by ambulance
2 Yes, by people who were with me
7 Don't Know

NALOXONE

The following questions will ask you about naloxone.

If DATE1 is equal to DATE2, then skip to instruction before K458.

For these next questions, we are going to return to asking you about things that have happened **since [DATE1] (that is, in the last [DAYS])**.

If Q3a is not equal to 1, then skip to K466.

If I276 is equal to 0 or I313 is equal to 0, then skip to K460.

K458. **In your lifetime**, have you ever received a take-home naloxone kit?

1 Yes
0 No

If K458 is equal to 1, then skip to instruction before K460.

K459. You reported that **in your lifetime**, you have **never** received a take-home naloxone kit. This includes either intranasal (nose spray) or injectable. (Choose one)

0 No, this is not correct. I think I HAVE received a naloxone kit in my lifetime.
1 Yes, this is correct. I have NEVER received a naloxone kit in my lifetime.

If K458 is not equal to 1, then skip to instruction before L477.

K460. What year did you first receive a take-home naloxone kit? (If you don't recall exactly, make your best guess.)

__ __ __ __ yyyy

K461. For how many opiate overdoses has naloxone been administered to you to reverse an opiate overdose?
(If you don't recall exactly, make your best guess.)

__ __ Times

K462. For how many opiate overdoses have you personally used naloxone on someone to try to reverse an opiate overdose? (If you don't recall exactly, make your best guess.)

__ __ Times

If K462 is less than 1, then skip to instruction before K464.

If K462 is equal to 1, then skip to instruction before K464.

K463. Of those [Response to K462] times, how many times did the persons live?

__ __ People
97 Don't Know

If K463 is greater than K462 then You cannot report more persons living than times that you administered naloxone. and skip to K463.

If K462 is equal to 0, then skip to instruction before K465.

If K462 is greater than 1, then skip to instruction before K465.

K464. Did the person live?

1 Yes
0 No
7 Don't Know

If K462 is less than 2, then skip to instruction before K466.

K465. You said you gave naloxone [Response to K462] times. How many different people was this? For example, did you give naloxone to 1 person [Response to K462] times? Or different people each time?

__ __ People

If K465 is greater than K462 then You cannot report giving naloxone to more persons than the number of times that you administered naloxone. and skip to K465.

K466. How comfortable are you that you could tell if someone was having an opiate overdose? (Choose one)

0 Very comfortable
1 Somewhat comfortable
2 A little comfortable
3 Not at all comfortable

K467. How comfortable are you that you would know what to do if someone was having an opiate overdose? (Choose one)

0 Very comfortable
1 Somewhat comfortable
2 A little comfortable
3 Not at all comfortable

K468. How comfortable are you that you could give naloxone to someone who was having an opiate overdose? (Choose one)

0 Very comfortable
1 Somewhat comfortable
2 A little comfortable
3 Not at all comfortable

K469. **Since [DATE1]** (that is, in the last [DAYS] days), have you had a naloxone kit at any point?

1 Yes
0 No

If K469 is equal to 0, then skip to instruction before L477.

K470. **Since [DATE1]** (that is, in the last [DAYS] days), how many times have you refilled your naloxone kit?

__ __ Times

If K470 is equal to 0, then skip to K472.

K471. Why did you need the most recent refill? (Choose one)

0 Lost
1 Gave away
2 Stolen
3 Used it on someone
4 Someone used it on me
5 Wanted a different kind
6 Wanted a second kit
7 Expired

K472. What type of naloxone was in your most recent kit? (Choose one)

0 Injectable (vial and syringe)
1 Intranasal (nasal spray)
2 Auto-injector (Evzio)
3 Injectable & Intranasal

K473. Where did you get your most recent kit? (Choose one)

00 A family member
01 A friend
02 Someone I use drugs with
03 Drug dealer
04 Syringe exchange
05 Other DOPE Project site
06 Methadone or buprenorphine (suboxone) program
07 Other drug treatment program
08 Pharmacy
09 Primary care provider
10 Found it
11 Other (specify)

If K473 is not equal to 11, then skip to K475.

K474. Please specify, where did you get your most recent kit?

__ __

K475. **Since [DATE1]** (that is, in the last [DAYS] days), where have you **usually** kept your naloxone? (Choose one)

0 With me
1 At my house/ apartment
2 With a partner, family member, or friend
3 Somewhere else

If B53 is equal to 0, then skip to instruction before L477.

K476. **Since [DATE1]** (that is, in the last [DAYS] days) you reported using opiates at least [FQOPTXT] days. On how many of those days did you have naloxone **with you** when you used opiates?

__ __ __ Days

If FCOPIATE is not equal to 11 and K476 is greater than F144 or FCOPIATE is equal to 11 and K476 is greater than DSOPIATE then You cannot have had naloxone with you when you used opiates more times than the number of day(s) that you reported using any opiates. and skip to K476.

DRUG TREATMENT

The following questions are going to ask you about your experience with drug treatment programs.

L477. To what extent would you say that you currently need help for your drug use? *(Choose one)* (Choose one)

0 No need
1 Some need
2 Great need
3 Urgent need

If Q3a is not equal to 1, then skip to L481.

L478. **In your lifetime**, have you ever received any type of professional help for your use of alcohol or drugs? By help we mean a detox or rehabilitation center, methadone, buprenorphine or other medication, or any other type of program or meeting that helps you reduce or stop your alcohol or drug use. (Choose one)

0 No
1 Yes, for drugs only
2 Yes, for alcohol only
3 Yes, for both drugs and alcohol

If L478 is equal to 0, then skip to instruction before M533.

L479. **In your lifetime**, what type of treatment have you received for your drug or alcohol use? *(Check all that apply)* (Check all that apply)

__ 12-step program
__ Residential drug or alcohol treatment
__ Outpatient substance use counseling
__ Methadone maintenance treatment
__ Buprenorphine (suboxone) treatment
__ Detoxification (a few days of intense care)
__ Vivitrol (naltrexone) treatment
__ Other (specify)

If L479H is not equal to 1, then skip to L481.

L480. Please specify, what treatment did you receive for your drug or alcohol use?

__ __

L481. **Since [DATE1]** (that is, in the last [DAYS] days), have you ever received any type of professional help for your use of alcohol or drugs? By help we mean a detox or rehabilitation center, methadone, buprenorphine or other medication, or any other type of program or meeting that helps you reduce or stop your alcohol or drug use. (Choose one)

0 No
1 Yes, for drugs only
2 Yes, for alcohol only
3 Yes, for both drugs and alcohol

If L481 is equal to 0, then skip to instruction before M533.

L482. **Since [DATE1]** (that is, in the last [DAYS] days), how many **days** were you engaged in **any** drug or alcohol treatment program?

__ __ __ Days

If L482 is greater than DAYS then Please limit your responce to only days since &[DATE1] (that is, in the last &[DAYS] days). and skip to L482.

L483. **Since [DATE1]** (that is, in the last [DAYS] days), what type of treatment have you received for your drug or alcohol use?
(Check all that apply) (Check all that apply)

__ 12-step program
__ Residential drug or alcohol treatment
__ Outpatient substance use counseling
__ Methadone maintenance treatment
__ Buprenorphine (suboxone) treatment
__ Detoxification (a few days of intense care)
__ Vivitrol (naltrexone) treatment
__ Other (specify)

If L483H is not equal to 1, then skip to instruction before L485.

L484. Please specify, what treatment did you receive for your drug or alcohol use?

__ __

If L483A is not equal to 1, then skip to instruction before L491.

Now we're going to ask you some questions about your most recent 12-step program.

L485. About when did you enter this 12-step program the MOST RECENT time?
(If you don't know the exact date, make your best guess.)

__ __ / __ __ / __ __ __ __ mm / dd / yyyy

If Q2 is greater than L485 then You cannot have entered treatment before you were born. and skip to L485.

L486. What are all the reasons that you decided to enter this most recent 12-step program?
(Check all that apply) (Check all that apply)

__ I was tired of using/ wanting to do more with my life
__ I wanted to avoid getting arrested
__ I was given a choice between treatment and incarceration, so I chose treatment
__ A partner, family member, or friend encouraged me to go
__ Had a child/ was pregnant
__ My kids were taken away
__ I was sick and needed medical attention
__ I overdosed
__ I witnessed someone overdose
__ I couldn't afford my drug use anymore
__ Other (specify)
__ Refuse to Answer

If L486N is not equal to 1, then skip to L488.

L487. Please specify, what other reason did you have for entering this most recent 12-step program?

__ __

L488. Are you still in this most recent 12-step program?

1 Yes
0 No

If L488 is equal to 1, then skip to instruction before L490.

L489. How long did you stay in this most recent 12-step program? (Choose one)

0 Less than 1 week
1 1 week to less than 3 weeks
2 3 weeks to less than 1 month
3 1 month to less than 3 months
4 3 months to less than 6 months
5 6 months or more

If L488 is equal to 1, then skip to instruction before L491.

L490. How many days did you stay off opiates once you left this most recent 12-step program?

__ __ Days

If DATE1N is less than L490 then Please limit your respose to days since &[DATE1] (that is, in the last &[DAYS] days). and skip to L490.

If L483B is not equal to 1, then skip to instruction before L497.

Now we're going to ask you some questions about your most recent residential drug or alcohol treatment.

L491. About when did you enter residential drug or alcohol treatment the MOST RECENT time?
(If you don't know the exact date, make your best guess.)

__ __ / __ __ / __ __ __ __ mm / dd / yyyy

If Q2 is greater than L491 then You cannot have entered treatment before you were born. and skip to L491.

L492. What are all the reasons that you decided to enter this most recent residential drug or alcohol treatment?
(Check all that apply) (Check all that apply)

- __ I was tired of using/ wanting to do more with my life
- __ I wanted to avoid getting arrested
- __ I was given a choice between treatment and incarceration, so I chose treatment
- __ A partner, family member, or friend encouraged me to go
- __ Had a child/ was pregnant
- __ My kids were taken away
- __ I was sick and needed medical attention
- __ I overdosed
- __ I witnessed someone overdose
- __ I couldn't afford my drug use anymore
- __ Other (specify)
- __ Refuse to Answer

If L492N is not equal to 1, then skip to L494.

L493. Please specify, what other reason did you have for entering this most recent drug or alcohol treatment?

__ __

L494. Are you still in this most recent residential drug or alcohol treatment?

1 Yes
0 No

If L494 is equal to 1, then skip to instruction before L496.

L495. How long did you stay in this most recent residential drug or alcohol treatment? (Choose one)

0 Less than 1 week
1 1 week to less than 3 weeks
2 3 weeks to less than 1 month
3 1 month to less than 3 months
4 3 months to less than 6 months
5 6 months or more

If L494 is equal to 1, then skip to instruction before L497.

L496. How many days did you stay off opiates once you left this most recent residential drug or alcohol treatment?

__ __ Days

If DATE1N is less than L496 then Please limit your respose to days since &[DATE1] (that is, in the last &[DAYS] days). and skip to L496.

If L483C is not equal to 1, then skip to instruction before L503.

Now we're going to ask you some questions about your most recent outpatient substance use counseling.

L497. About when did you enter outpatient substance use counseling the MOST RECENT time?
(If you don't know the exact date, make your best guess.)

__ __ / __ __ / __ __ __ __ mm / dd / yyyy

If Q2 is greater than L497 then You cannot have entered treatment before you were born. and skip to L497.

L498. What are all the reasons that you decided to enter this most recent outpatient substance use counseling?
(Check all that apply) (Check all that apply)

__ I was tired of using/ wanting to do more with my life
__ I wanted to avoid getting arrested
__ I was given a choice between treatment and incarceration, so I chose treatment
__ A partner, family member, or friend encouraged me to go
__ Had a child/ was pregnant
__ My kids were taken away
__ I was sick and needed medical attention
__ I overdosed
__ I witnessed someone overdose
__ I couldn't afford my drug use anymore
__ Other (specify)
__ Refuse to Answer

If L498N is not equal to 1, then skip to L500.

L499. Please specify, what other reason did you have for entering this most recent outpatient substance use counseling?

__ __

L500. Are you still in this most recent outpatient substance use counseling?
1 Yes
0 No

If L500 is equal to 1, then skip to instruction before L502.

L501. How long did you stay in this most recent outpatient substance use counseling? (Choose one)
0 Less than 1 week
1 1 week to less than 3 weeks
2 3 weeks to less than 1 month
3 1 month to less than 3 months
4 3 months to less than 6 months
5 6 months or more

If L500 is equal to 1, then skip to instruction before L503.

L502. How many days did you stay off opiates once you left this most recent outpatient substance use counseling?

__ __ __ __ __ DAYS

If DATE1N is less than L502 then Please limit your respose to days since &[DATE1] (that is, in the last &[DAYS] days). and skip to L502.
If L483D is not equal to 1, then skip to instruction before L509.

Now we're going to ask you some questions about your most recent methadone maintenance treatment.

L503. About when did you enter methadone maintenance treatment the MOST RECENT time?
(If you don't know the exact date, make your best guess.)

__ __ / __ __ / __ __ __ __ mm / dd / yyyy

If Q2 is greater than L503 then You cannot have entered treatment before you were born. and skip to L503.

L504. What are all the reasons that you decided to enter this most recent methadone maintenance treatment?
(Check all that apply) (Check all that apply)

__ I was tired of using/ wanting to do more with my life
__ I wanted to avoid getting arrested
__ I was given a choice between treatment and incarceration, so I chose treatment
__ A partner, family member, or friend encouraged me to go
__ Had a child/ was pregnant
__ My kids were taken away
__ I was sick and needed medical attention
__ I overdosed
__ I witnessed someone overdose
__ I couldn't afford my drug use anymore
__ Other (specify)
__ Refuse to Answer

If L504N is not equal to 1, then skip to L506.

L505. Please specify, what other reason did you have for entering this most recent methadone maintenance treatment?

__ __

L506. Are you still in this most recent methadone maintenance treatment?

1 Yes
0 No

If L506 is equal to 1, then skip to instruction before L508.

L507. How long were you in this most recent methadone maintenance treatment? (Choose one)

0 Less than 1 week
1 1 week to less than 3 weeks
2 3 weeks to less than 1 month
3 1 month to less than 3 months
4 3 months to less than 6 months
5 6 months or more

If L506 is equal to 1, then skip to instruction before L509.

L508. How days did you not use opiates once you left this most recent methadone maintenance treatment?

__ __ Days

If DATE1N is less than L508 then Please limit your respose to days since &[DATE1] (that is, in the last &[DAYS] days). and skip to L508.

If L483E is not equal to 1, then skip to instruction before L515.

Now we're going to ask you some questions about your most recent buprenorphine (suboxone) treatment.

L509. About when did you enter buprenorphine (suboxone) treatment the MOST RECENT time?
(If you don't know the exact date, make your best guess.)

__ __ / __ __ / __ __ __ __ mm / dd / yyyy

If Q2 is greater than L509 then You cannot have entered treatment before you were born. and skip to L509.

L510. What are all the reasons that you decided to enter this most recent buprenorphine (suboxone) treatment?
(Check all that apply) (Check all that apply)

- __ I was tired of using/ wanting to do more with my life
- __ I wanted to avoid getting arrested
- __ I was given a choice between treatment and incarceration, so I chose treatment
- __ A partner, family member, or friend encouraged me to go
- __ Had a child/ was pregnant
- __ My kids were taken away
- __ I was sick and needed medical attention
- __ I overdosed
- __ I witnessed someone overdose
- __ I couldn't afford my drug use anymore
- __ Other (specify)
- __ Refuse to Answer

If L510N is not equal to 1, then skip to L512.

L511. Please specify, what other reason did you have for entering this most recent buprenorphine (suboxone) treatment?

__ __

L512. Are you still in this most recent buprenorphine (suboxone) treatment?

1	Yes
0	No

If L512 is equal to 1, then skip to instruction before L514.

L513. How long were you in this most recent buprenorphine (suboxone) treatment? (Choose one)

0	Less than 1 week
1	1 week to less than 3 weeks
2	3 weeks to less than 1 month
3	1 month to less than 3 months
4	3 months to less than 6 months
5	6 months or more

If L512 is equal to 1, then skip to instruction before L515.

L514. How many days did you not use opiates once you left this most recent buprenorphine (suboxone) treatment?

__ __ Days

If DATE1N is less than L514 then Please limit your respose to days since &[DATE1] (that is, in the last &[DAYS] days). and skip to L514.

If L483F is not equal to 1, then skip to instruction before L521.

Now we're going to ask you some questions about your most recent detoxification (a few days of intense care).

L515. About when did you enter detoxification the MOST RECENT time? By detoxification, we mean at least a few days of intense care.
(If you don't know the exact date, make your best guess.)

__ __ / __ __ / __ __ __ __ mm / dd / yyyy

If Q2 is greater than L515 then You cannot have entered treatment before you were born. and skip to L515.

L516. What are all the reasons that you decided to enter this most recent detoxification?
(Check all that apply) (Check all that apply)

- __ I was tired of using/ wanting to do more with my life
- __ I wanted to avoid getting arrested
- __ I was given a choice between treatment and incarceration, so I chose treatment
- __ A partner, family member, or friend encouraged me to go
- __ Had a child/ was pregnant
- __ My kids were taken away
- __ I was sick and needed medical attention
- __ I overdosed
- __ I witnessed someone overdose
- __ I couldn't afford my drug use anymore
- __ Other (specify)
- __ Refuse to Answer

If L516N is not equal to 1, then skip to L518.

L517. Please specify, what other reason did you have for entering this most recent detoxification?

__ __

L518. Are you still in this most recent detoxification?

1	Yes
0	No

If L518 is equal to 1, then skip to instruction before L520.

L519. How long were you in this most recent detoxification? (Choose one)

0	Less than 1 week
1	1 week to less than 3 weeks
2	3 weeks to less than 1 month
3	1 month to less than 3 months
4	3 months to less than 6 months
5	6 months or more

If L518 is equal to 1, then skip to instruction before L521.

L520. How many days did you not use opiates once you left this most recent detoxification?

__ __ Days

If DATE1N is less than L520 then Please limit your respose to days since &[DATE1] (that is, in the last &[DAYS] days). and skip to L520.

If L483G is not equal to 1, then skip to instruction before L527.

Now we're going to ask you some questions about your most recent vivitrol (naltrexone) treatment.

L521. About when did you enter Vivitrol treatment the MOST RECENT time?
(If you don't know the exact date, make your best guess.)

__ __ / __ __ / __ __ __ __ mm / dd / yyyy

If Q2 is greater than L521 then You cannot have entered treatment before you were born. and skip to L521.

L522. What are all the reasons that you decided to enter Vivitrol treatment this most recent time? *(Check all that apply)* (Check all that apply)

- __ I was tired of using/ wanting to do more with my life
- __ I wanted to avoid getting arrested
- __ I was given a choice between treatment and incarceration, so I chose treatment
- __ A partner, family member, or friend encouraged me to go
- __ Had a child/ was pregnant
- __ My kids were taken away
- __ I was sick and needed medical attention
- __ I overdosed
- __ I witnessed someone overdose
- __ I couldn't afford my drug use anymore
- __ Other (specify)
- __ Refuse to Answer

If L522N is not equal to 1, then skip to L524.

L523. Please specify, what other reason did you have for entering Vivitrol treatment this most recent time?

__ __

L524. Are you still in this most recent Vivitrol treatment?

1 Yes
0 No

If L524 is equal to 1, then skip to instruction before L526.

L525. How long were you in Vivitrol treatment this most recent time? (Choose one)

0 Less than 1 week
1 1 week to less than 3 weeks
2 3 weeks to less than 1 month
3 1 month to less than 3 months
4 3 months to less than 6 months
5 6 months or more

If L524 is equal to 1, then skip to instruction before L527.

L526. How many days did you not use opiates once you left Vivitrol treatment this most recent time?

__ __ Days

If DATE1N is less than L526 then Please limit your respose to days since &[DATE1] (that is, in the last &[DAYS] days). and skip to L526.

If L483H is not equal to 1, then skip to instruction before M533.

Now we're going to ask you some questions about your most recent other treatment.

L527. About when did you enter this other treatment the MOST RECENT time? *(If you don't know the exact date, make your best guess.)*

__ __ / __ __ / __ __ __ __ mm / dd / yyyy

If Q2 is greater than L527 then You cannot have entered treatment before you were born. and skip to L527.

L528. What are all the reasons that you decided to enter this most recent other treatment?
(Check all that apply) (Check all that apply)

__ I was tired of using/ wanting to do more with my life
__ I wanted to avoid getting arrested
__ I was given a choice between treatment and incarceration, so I chose treatment
__ A partner, family member, or friend encouraged me to go
__ Had a child/ was pregnant
__ My kids were taken away
__ I was sick and needed medical attention
__ I overdosed
__ I witnessed someone overdose
__ I couldn't afford my drug use anymore
__ Other (specify)
__ Refuse to Answer

If L528N is not equal to 1, then skip to L530.

L529. Please specify, what other reason did you have for entering this most recent other treatment?

__ __

L530. Are you still in this most recent treatment?

1 Yes
0 No

If L530 is equal to 1, then skip to instruction before L532.

L531. How long did you stay in this other treatment? (Choose one)

0 Less than 1 week
1 1 week to less than 3 weeks
2 3 weeks to less than 1 month
3 1 month to less than 3 months
4 3 months to less than 6 months
5 6 months or more

If L530 is equal to 1, then skip to instruction before M533.

L532. How many days did you stay off opiates once you left this most recent other treatment?

__ __ Days

If DATE1N is less than L532 then Please limit your respose to days since &[DATE1] (that is, in the last &[DAYS] days). and skip to L532.

JAIL/ PRISON QUESTIONS

Now we will ask you about arrest experiences you have had.

If Q3a is not equal to 1, then skip to instruction before M534.

M533. Have you ever been arrested by law enforcement? By arrest we mean a time when police have taken you to the police station and there was a formal record made of this event.

1	Yes
0	No
8	Refuse to Answer

If M533 is equal to 0 or M533 is equal to 8, then skip to instruction before N537.

M534. **Since [DATE1]** (that is, in the last [DAYS] days), how many times were you arrested?

__ __	Times
98	Refuse to Answer

If M534 is equal to 0 or M534 is equal to 98, then skip to instruction before N537.

M535. Of the [Response to M534] time(s) you were arrested since [DATE1] (that is, in the last [DAYS] days), how many time(s) were you sentenced to **jail or prison**?

__ __	Times
97	Don't Know
98	Refuse to Answer

If M535 is greater than M534 then Your response should be less than or equal to the number of times you were arrested since &[DATE1]. and skip to M535.

M536. Of the [Response to M534] time(s) you were arrested since [DATE1] (that is, in the last [DAYS] days), how many time(s) were you sentenced to **compulsory drug treatment**?

__ __	Times
97	Don't Know
98	Refuse to Answer

If M536 is greater than M534 then Your response should be less than or equal to the number of times you were arrested since &[DATE1]. and skip to M536.

HOSPITALIZATION QUESTIONS

Now we will ask you about hospitalizations you have had.

N537. **Since [DATE1]** (that is, in the last [DAYS] days), how many times have you been to the **emergency department (ER)**?

__ __	Times
98	Refuse to Answer

If N537 is equal to 0, then skip to instruction before P543.

N538. Of the [Response to N537] time(s), how many time(s) were you admitted to the hospital - that means you got a bed in the hospital and stayed at least overnight?

__ __	Times
98	Refuse to Answer

If N538 is greater than N537 then Your response shoudl be less than the total number of times that you have been to the emergency department since &[DATE1]. and skip to instruction before N538.

If N538 is equal to 0, then skip to instruction before P543.

N539. When was the last time you were admitted to the hospital? (If you don't recall exactly, make your best guess.)

__ __ / __ __ / __ __ __ __	mm / dd / yyyy
2098	Refuse to Answer (Year)

If Date(N539Y, N539M, N539D) is less than DATE1N and N539Y is not equal to 2098 then We're looking for your most recent admission to the hospital since &[DATE1]. Your response should be after this date. and skip to N539.

N540. How long did you stay in the hospital that time? (If you don't recall exactly, make your best guess.)

__ __ __	Days
998	Refuse to Answer

N541. Why were you admitted to the hospital that time?
(*Check all that apply*) (Check all that apply)

__ Overdose
__ Pneumonia - lung infection
__ Skin infection, including cellulitis, necrotizing fasciitis, and others
__ Endocarditis - a heart valve infection
__ Other infection
__ Heart problem
__ Liver problem
__ Kidney problem
__ Neurologic problem, such as a stroke
__ Detox
__ Other (specify)
__ Refuse to Answer

If N541K is not equal to 1, then skip to instruction before P543.

N542. Please specify, why else were you admitted to the hospital that time?

__ __

SEVERITY OF DEPENDENCE

If B54A is not equal to 1, then skip to instruction before P548.

The following questions are about your use of heroin **since [DATE1]** (that is, in the last [DAYS] days). For each of the five questions, please indicate the most appropriate response, as it applied to your use of heroin since [DATE1].

P543. **Since [DATE1]** (that is, in the last [DAYS] days), did you ever think your use of heroin was out of control? (Choose one)

0 Never/ almost never
1 Sometimes
2 Often
3 Always/ nearly always

P544. **Since [DATE1]** (that is, in the last [DAYS] days), did the prospect of missing a fix (or dose) of heroin make you anxious or worried? (Choose one)

0 Never/ almost never
1 Sometimes
2 Often
3 Always/ nearly always

P545. **Since [DATE1]** (that is, in the last [DAYS] days), did you worry about your use of heroin? (Choose one)

0 Never/ almost never
1 Sometimes
2 Often
3 Always/ nearly always

P546. **Since [DATE1]** (that is, in the last [DAYS] days), did you wish you could stop using heroin? (Choose one)

0 Never/ almost never
1 Sometimes
2 Often
3 Always/ nearly always

P547. How difficult would you find it to stop or go without heroin? (Choose one)

0 Not difficult
1 Quite difficult
2 Very difficult
3 Impossible

If MPRSC is not equal to 1, then skip to instruction before P553.

The following questions are about your use of prescription opiates not as prescribed **since [DATE1]** (that is, in the last [DAYS] days). For each of the five questions, please indicate the most appropriate response, as it applied to your use of prescription opiates since [DATE1].

P548. **Since [DATE1]** (that is, in the last [DAYS] days), did you ever think your use of prescription opiates was out of control? (Choose one)

0 Never/ almost never
1 Sometimes
2 Often
3 Always/ nearly always

P549. **Since [DATE1]** (that is, in the last [DAYS] days), did the prospect of missing a fix (or dose) of prescription opiates make you anxious or worried? (Choose one)

0 Never/ almost never
1 Sometimes
2 Often
3 Always/ nearly always

P550. **Since [DATE1]** (that is, in the last [DAYS] days), did you worry about your use of prescription opiates? (Choose one)

0 Never/ almost never
1 Sometimes
2 Often
3 Always/ nearly always

P551. **Since [DATE1]** (that is, in the last [DAYS] days), did you wish you could stop using prescription opiates? (Choose one)

0 Never/ almost never
1 Sometimes
2 Often
3 Always/ nearly always

P552. How difficult would you find it to stop or go without prescription opiates? (Choose one)

0 Not difficult
1 Quite difficult
2 Very difficult
3 Impossible

If B96A is not equal to 1, then skip to instruction before Q1a.

The following questions are about your use of alcohol **since [DATE1]** (that is, in the last [DAYS] days). For each of the three questions, please indicate the most appropriate response, as it applied to your use of alcohol since [DATE1].

P553. **Since [DATE1]** (that is, in the last [DAYS] days), how often did you have a drink containing alcohol? (Choose one)

0 Never
1 Monthly or less
2 2-4 times a month
3 2-3 times a week
4 4 or more times a week
8 Refuse to Answer

P554. **Since [DATE1]** (that is, in the last [DAYS]), how many standard drinks containing alcohol did you have on a typical day?

A standard drink is approximately one 12oz of beer, one 5oz glass of wine, or one 1.5oz shot of hard liquor. (Choose one)

0 1 or 2
1 3 or 4
2 5 or 6
3 7 or 9
4 10 or more
8 Refuse to Answer

P555. **Since [DATE1]** (that is, in the last [DAYS]), how often do you have six or more drinks on one occasion? (Choose one)

0 Never
1 Less than monthly
2 Monthly
3 Weekly
4 Daily or almost daily
8 Refuse to Answer

Depression scale

In this next section, we are going to ask about your mood in the **past week**. After each statement, please indicate how often you felt that way.

Q1a. **In the past week**, I was bothered by things that usually don't bother me. (Choose one)

1 Rarely or none of the time, (less than 1 day)
2 Some or a little of the time, (1 to 2 days)
3 Occasionally or a moderate amount of time, (3 to 4 days)
4 Most or all of the time, (5 to 7 days)

Q1b. **In the past week**, I did not feel like eating. My appetite was poor. (Choose one)

1 Rarely or none of the time, (less than 1 day)
2 Some or a little of the time, (1 to 2 days)
3 Occasionally or a moderate amount of time, (3 to 4 days)
4 Most or all of the time, (5 to 7 days)

Q1c. **In the past week**, I felt that I could not shake off the blues even with help from my family or friends. (Choose one)

1 Rarely or none of the time, (less than 1 day)
2 Some or a little of the time, (1 to 2 days)
3 Occasionally or a moderate amount of time, (3 to 4 days)
4 Most or all of the time, (5 to 7 days)

Q1d. **In the past week**, I felt that I was just as good as other people. (Choose one)

1 Rarely or none of the time, (less than 1 day)
2 Some or a little of the time, (1 to 2 days)
3 Occasionally or a moderate amount of time, (3 to 4 days)
4 Most or all of the time, (5 to 7 days)

Q1e. **In the past week**, I had trouble keeping my mind on what I was doing. (Choose one)

1 Rarely or none of the time, (less than 1 day)
2 Some or a little of the time, (1 to 2 days)
3 Occasionally or a moderate amount of time, (3 to 4 days)
4 Most or all of the time, (5 to 7 days)

Q1f. **In the past week**, I felt depressed. (Choose one)

1 Rarely or none of the time, (less than 1 day)
2 Some or a little of the time, (1 to 2 days)
3 Occasionally or a moderate amount of time, (3 to 4 days)
4 Most or all of the time, (5 to 7 days)

Q1g. **In the past week**, I felt like everything I did was an effort. (Choose one)

1 Rarely or none of the time, (less than 1 day)
2 Some or a little of the time, (1 to 2 days)
3 Occasionally or a moderate amount of time, (3 to 4 days)
4 Most or all of the time, (5 to 7 days)

Q1h. **In the past week**, I felt hopeful about the future. (Choose one)

1 Rarely or none of the time, (less than 1 day)
2 Some or a little of the time, (1 to 2 days)
3 Occasionally or a moderate amount of time, (3 to 4 days)
4 Most or all of the time, (5 to 7 days)

Q1i. **In the past week**, I thought my life had been a failure. (Choose one)

1 Rarely or none of the time, (less than 1 day)
2 Some or a little of the time, (1 to 2 days)
3 Occasionally or a moderate amount of time, (3 to 4 days)
4 Most or all of the time, (5 to 7 days)

Q1j. **In the past week**, I felt fearful. (Choose one)
1 Rarely or none of the time, (less than 1 day)
2 Some or a little of the time, (1 to 2 days)
3 Occasionally or a moderate amount of time, (3 to 4 days)
4 Most or all of the time, (5 to 7 days)

Q1k. **In the past week**, my sleep was restless. (Choose one)
1 Rarely or none of the time, (less than 1 day)
2 Some or a little of the time, (1 to 2 days)
3 Occasionally or a moderate amount of time, (3 to 4 days)
4 Most or all of the time, (5 to 7 days)

Q1l. **In the past week**, I was happy. (Choose one)
1 Rarely or none of the time, (less than 1 day)
2 Some or a little of the time, (1 to 2 days)
3 Occasionally or a moderate amount of time, (3 to 4 days)
4 Most or all of the time, (5 to 7 days)

Q1m. **In the past week**, I talked less than usual. (Choose one)
1 Rarely or none of the time, (less than 1 day)
2 Some or a little of the time, (1 to 2 days)
3 Occasionally or a moderate amount of time, (3 to 4 days)
4 Most or all of the time, (5 to 7 days)

Q1n. **In the past week**, I felt lonely. (Choose one)
1 Rarely or none of the time, (less than 1 day)
2 Some or a little of the time, (1 to 2 days)
3 Occasionally or a moderate amount of time, (3 to 4 days)
4 Most or all of the time, (5 to 7 days)

Q1o. **In the past week**, people were unfriendly. (Choose one)
1 Rarely or none of the time, (less than 1 day)
2 Some or a little of the time, (1 to 2 days)
3 Occasionally or a moderate amount of time, (3 to 4 days)
4 Most or all of the time, (5 to 7 days)

Q1p. **In the past week**, I enjoyed life. (Choose one)
1 Rarely or none of the time, (less than 1 day)
2 Some or a little of the time, (1 to 2 days)
3 Occasionally or a moderate amount of time, (3 to 4 days)
4 Most or all of the time, (5 to 7 days)

Q1q. **In the past week**, I had crying spells. (Choose one)
1 Rarely or none of the time, (less than 1 day)
2 Some or a little of the time, (1 to 2 days)
3 Occasionally or a moderate amount of time, (3 to 4 days)
4 Most or all of the time, (5 to 7 days)

Q1r. **In the past week**, I felt sad. (Choose one)
1 Rarely or none of the time, (less than 1 day)
2 Some or a little of the time, (1 to 2 days)
3 Occasionally or a moderate amount of time, (3 to 4 days)
4 Most or all of the time, (5 to 7 days)

Q1s. **In the past week**, I felt that people disliked me. (Choose one)
1 Rarely or none of the time, (less than 1 day)
2 Some or a little of the time, (1 to 2 days)
3 Occasionally or a moderate amount of time, (3 to 4 days)
4 Most or all of the time, (5 to 7 days)

Q1t. **In the past week**, I could not get going. (Choose one)

1 Rarely or none of the time, (less than 1 day)
2 Some or a little of the time, (1 to 2 days)
3 Occasionally or a moderate amount of time, (3 to 4 days)
4 Most or all of the time, (5 to 7 days)

In the previous questions, we asked about any emotional or physical concerns you may be having. **If you want help with any of these concerns, please be sure to tell your study clinician or counselor because they do NOT have access to your answers.**

SEXUAL BEHAVIOR

Now we are going to ask you some questions about your sexual behaviors.
For the purposes of this study, please keep the following in mind:

When we ask about SEXUAL INTERCOURSE, we mean either ANAL or VAGINAL SEX.

-by ANAL SEX, we mean having a penis in an anus, with or without ejaculation.

-by VAGINAL SEX, we mean having a penis in a vagina, with or without ejaculation.
When we ask about CONDOM USE, please include the times that a condom was used from when you started having sex and was kept on the whole time. Please do not include the times when the condom may have been taken off, slipped off, or broken.

S2. Since [DATE1] (that is, in the last [DAYS] days), did you have any sexual intercourse? (By sexual intercourse, we mean either anal or vaginal sex.)

1	Yes
0	No
8	Refuse to Answer

If S2 is equal to 1, then skip to instruction before S4.

S3. Since [DATE1] (that is, in the last [DAYS] days), did you have any primary partners?

1	Yes
0	No
8	Refuse to Answer

If S3 is equal to 0, then skip to instruction before T53.

This section will ask you about your sexual behavior with **FEMALE** partners, **since [DATE1]** (that is, in the last [DAYS] days).

S4. **Since [DATE1]** (that is, in the last [DAYS] days), were you in a **primary** relationship with a FEMALE?

1	Yes
0	No
8	Refuse to Answer

If S4 is equal to 0, then skip to instruction before S8.

S5. What was your most recent female **primary** partner's HIV status? *(Choose one)* (Choose one)

0	HIV positive
1	HIV negative
2	I am unsure
8	Refuse to Answer

If S2 is equal to 0, then skip to S7.

S6. Since [DATE1] (that is, in the last [DAYS] days), did you have sexual intercourse with this most recent female primary partner, WITHOUT using a condom?

1	Yes
0	No
8	Refuse to Answer

S7. Are you still in a **primary** relationship with this female now?

1	Yes
0	No
8	Refuse to Answer

If S2 is equal to 0, then skip to instruction before S16.

S8. **Since [DATE1]** (that is, in the last [DAYS] days), how many **non-primary** FEMALE sexual partners have you had? Include women you had sexual intercourse with, with or without a condom.

__ __ __	Partners
998	Refuse to Answer

If S8 is equal to 0, then skip to instruction before S16.

S9. **Since [DATE1]** (that is, in the last [DAYS] days), of these [Response to S8], **non-primary** FEMALE sexual partners, how many did you not know the HIV status of or were unsure of the HIV status of before you had sexual intercourse with, with or without a condom?

__ __ __	Partners
998	Refuse to Answer

If S9 is greater than S8 then This number cannot be greater than &[FENONPRM]. and skip to S9.

If S9 is equal to 0, then skip to S11.

S10. **Since [DATE1]** (that is, in the last [DAYS] days), with how many of these [Response to S9] women whose HIV status you didn't know or were unsure about did you have sexual intercourse, WITHOUT using a condom?

__ __ __	Partners
998	Refuse to Answer

If S10 is greater than S9 then This number cannot be greater than &[FEUNK]. and skip to S10.

S11. **Since [DATE1]** (that is, in the last [DAYS] days), you have had sex with [FEKNW] **non-primary** FEMALE sexual partners where you KNEW their HIV status before you had sex with them. (Choose one)

0	[illegible]
1	[illegible]
8	[illegible]

If S11 is equal to 0 then skip to S9.

If FEKNW is equal to 0, then skip to instruction before S16.

S12. **Since [DATE1]** (that is, in the last [DAYS] days), how many of these [FEKNW] **non-primary** FEMALE sexual partners were HIV positive at the time you had sex with them?

__ __ __	Partners
998	Refuse to Answer

If S12 is greater than FEKNW then This number cannot be greater than &[FEKNW]. and skip to S12.

If S12 is equal to 0, then skip to S14.

S13. **Since [DATE1]** (that is, in the last [DAYS] days), with how many of the [Response to S12] HIV positive women did you have sexual intercourse, WITHOUT using a condom?

__ __ __	Partners
998	Refuse to Answer

If S13 is greater than S12 then This number cannot be greater than &[FEPOS]. and skip to S13.

S14. **Since [DATE1]** (that is, in the last [DAYS] days), you have had sex with [FENEG] women who you believed were HIV negative before you had sex with them. (Choose one)

0	No, I wa
1	Yes, I ha
8	Refuse t

If S14 is equal to 0 then skip to S12.

If FENEG is equal to 0, then skip to instruction before S16.

S15. **Since [DATE1]** (that is, in the last [DAYS] days), with how many of the [FENEG] HIV negative women did you have sexual intercourse, WITHOUT using a condom?

__ __ __	Partners
998	Refuse to Answer

If S15 is greater than FENEG then This number cannot be greater than &[FENEG] and skip to S15.

This section will ask you about your sexual behavior with **MALE** partners, **since [DATE1]** (that is, in the last [DAYS] days).

S16. **Since [DATE1]** (that is, in the last [DAYS] days), were you in a **primary** relationship with a MALE?

1 Yes
0 No
8 Refuse to Answer

If S16 is equal to 0, then skip to instruction before S20.

S17. What was your most recent male **primary** partner's HIV status? *(Choose one)* (Choose one)

0 HIV positive
1 HIV negative
2 I am unsure
8 Refuse to Answer

If S2 is equal to 0, then skip to S19.

S18. Since [DATE1] (that is, in the last [DAYS] days), did you have sexual intercourse with this most recent male primary partner, WITHOUT using a condom?

1 Yes
0 No
8 Refuse to Answer

S19. Are you still in a **primary** relationship with this male now?

1 Yes
0 No
8 Refuse to Answer

If S2 is equal to 0, then skip to instruction before S28.

S20. **Since [DATE1]** (that is, in the last [DAYS] days), how many **non-primary** MALE sexual partners have you had? Include men you had sexual intercourse with, with or without a condom.

__ __ __ Partners
998 Refuse to Answer

If S20 is equal to 0, then skip to instruction before S28.

S21. **Since [DATE1]** (that is, in the last [DAYS] days), of these [Response to S20], **non-primary** MALE sexual partners, how many did you not know the HIV status of or were unsure of the HIV status of before you had sexual intercourse with, with or without a condom?

__ __ __ Partners
998 Refuse to Answer

If S21 is greater than S20 then This number cannot be greater than &[MANONPRM]. and skip to S21.
If S21 is equal to 0, then skip to S23.

S22. **Since [DATE1]** (that is, in the last [DAYS] days), with how many of these [Response to S21] men whose HIV status you didn't know or were unsure about did you have sexual intercourse, WITHOUT using a condom?

__ __ __ Partners
998 Refuse to Answer

If S22 is greater than S21 then This number cannot be greater than &[MAUNK]. and skip to S22.

S23. **Since [DATE1]** (that is, in the last [DAYS] days), you have had sex with [MAKNW] **non-primary** MALE sexual partners where you KNEW their HIV status before you had sex with them. (Choose one)

0 [illegible]
1 [illegible]
8 [illegible]

If S23 is equal to 0 then skip to S21.
If MAKNW is equal to 0, then skip to instruction before S28.

S24. **Since [DATE1]** (that is, in the last [DAYS] days), how many of these [MAKNW] **non-primary** MALE sexual partners were HIV positive at the time you had sex with them?

__ __ __	Partners
998	Refuse to Answer

If S24 is greater than MAKNW then This number cannot be greater than &[MAKNW]. and skip to S24.
If S24 is equal to 0, then skip to S26.

S25. **Since [DATE1]** (that is, in the last [DAYS] days), with how many of the [Response to S24] HIV positive men did you have sexual intercourse, WITHOUT using a condom?

__ __ __	Partners
998	Refuse to Answer

If S25 is greater than S24 then This number cannot be greater than &[MAPOS]. and skip to S25.

S26. **Since [DATE1]** (that is, in the last [DAYS] days), you have had sex with [MANEG] men who you believed were HIV negative before you had sex with them. (Choose one)

0	No, I wa
1	Yes, I ha
8	Refuse t

If S26 is equal to 0 then skip to S24.
If MANEG is equal to 0, then skip to instruction before S28.

S27. **Since [DATE1]** (that is, in the last [DAYS] days), with how many of the [MANEG] HIV negative men did you have sexual intercourse, WITHOUT using a condom?

__ __ __	Partners
998	Refuse to Answer

If S27 is greater than MANEG then This number cannot be greater than &[MANEG] and skip to S27.

This section will ask you about your sexual behavior with **TRANSFEMALE** partners, **since [DATE1]** (that is, in the last [DAYS] days).

S28. **Since [DATE1]** (that is, in the last [DAYS] days), were you in a **primary** relationship with a TRANSFEMALE?

1	Yes
0	No
8	Refuse to Answer

If S28 is equal to 0, then skip to instruction before S32.

S29. What was your most recent transfemale **primary** partner's HIV status? *(Choose one)* (Choose one)

0	HIV positive
1	HIV negative
2	I am unsure
8	Refuse to Answer

If S2 is equal to 0, then skip to S31.

S30. Since [DATE1] (that is, in the last [DAYS] days), did you have sexual intercourse with this most recent transfemale primary partner, WITHOUT using a condom?

1	Yes
0	No
8	Refuse to Answer

S31. Are you still in a **primary** relationship with this transfemale now?

1	Yes
0	No
8	Refuse to Answer

If S2 is equal to 0, then skip to instruction before S40.

S32. **Since [DATE1]** (that is, in the last [DAYS] days), how many **non-primary** TRANSFEMALE sexual partners have you had? Include transwomen you had sexual intercourse with, with or without a condom.

__ __ __ Partners
998 Refuse to Answer

If S32 is equal to 0, then skip to instruction before S40.

S33. **Since [DATE1]** (that is, in the last [DAYS] days), of these [Response to S32], **non-primary** TRANSFEMALE sexual partners, how many did you not know the HIV status of or were unsure of the HIV status of before you had sexual intercourse with, with or without a condom?

__ __ __ Partners
998 Refuse to Answer

If S33 is greater than S32 then This number cannot be greater than &[TFNONPRM]. and skip to S33.
If S33 is equal to 0, then skip to S35.

S34. **Since [DATE1]** (that is, in the last [DAYS] days), with how many of these [Response to S33] transwomen whose HIV status you didn't know or were unsure about did you have sexual intercourse, WITHOUT using a condom?

__ __ __ Partners
998 Refuse to Answer

If S34 is greater than S33 then This number cannot be greater than &[TFUNK]. and skip to S34.

S35. **Since [DATE1]** (that is, in the last [DAYS] days), you have had sex with [TFKNW] **non-primary** TRANSFEMALE sexual partners where you KNEW their HIV status before you had sex with them. (Choose one)

0 |
1 `
8 |

If S35 is equal to 0 then skip to S33.
If TFKNW is equal to 0, then skip to instruction before S40.

S36. **Since [DATE1]** (that is, in the last [DAYS] days), how many of these [TFKNW] **non-primary** TRANSFEMALE sexual partners were HIV positive at the time you had sex with them?

__ __ __ Partners
998 Refuse to Answer

If S36 is greater than TFKNW then This number cannot be greater than &[TFKNW]. and skip to S36.
If S36 is equal to 0, then skip to S38.

S37. **Since [DATE1]** (that is, in the last [DAYS] days), with how many of the [Response to S36] HIV positive transwomen did you have sexual intercourse, WITHOUT using a condom?

__ __ __ Partners
998 Refuse to Answer

If S37 is greater than S36 then This number cannot be greater than &[TFPOS]. and skip to S37.

S38. **Since [DATE1]** (that is, in the last [DAYS] days), you have had sex with [TFNEG] transwomen who you believed were HIV negative before you had sex with them. (Choose one)

0 |
1 `
8 |

If S38 is equal to 0 then skip to S36.
If TFNEG is equal to 0, then skip to instruction before S40.

S39. **Since [DATE1]** (that is, in the last [DAYS] days), with how many of the [TFNEG] HIV negative transwomen did you have sexual intercourse, WITHOUT using a condom?

__ __ __ Partners
998 Refuse to Answer

If S39 is greater than TFNEG then This number cannot be greater than &[TFNEG] and skip to S39.

This section will ask you about your sexual behavior with **TRANSMALE** partners, **since [DATE1]** (that is, in the last [DAYS] days).

S40. **Since [DATE1]** (that is, in the last [DAYS] days), were you in a **primary** relationship with a TRANSMALE?

1 Yes
0 No
8 Refuse to Answer

If S40 is equal to 0, then skip to instruction before S44.

S41. What was your most recent transmale **primary** partner's HIV status? *(Choose one)* (Choose one)

0 HIV positive
1 HIV negative
2 I am unsure
8 Refuse to Answer

If S2 is equal to 0, then skip to S43.

S42. Since [DATE1] (that is, in the last [DAYS] days), did you have sexual intercourse with this most recent transmale primary partner, WITHOUT using a condom?

1 Yes
0 No
8 Refuse to Answer

S43. Are you still in a **primary** relationship with this transmale now?

1 Yes
0 No
8 Refuse to Answer

If S2 is equal to 0, then skip to instruction before T53.

S44. **Since [DATE1]** (that is, in the last [DAYS] days), how many **non-primary** TRANSMALE sexual partners have you had? Include transmen you had sexual intercourse with, with or without a condom.

__ __ __ Partners
998 Refuse to Answer

If S44 is equal to 0, then skip to instruction before S52.

S45. **Since [DATE1]** (that is, in the last [DAYS] days), of these [Response to S44], **non-primary** TRANSMALE sexual partners, how many did you not know the HIV status of or were unsure of the HIV status of before you had sexual intercourse with, with or without a condom?

__ __ __ Partners
998 Refuse to Answer

If S45 is greater than S44 then This number cannot be greater than &[TMNONPRM]. and skip to S45.

If S45 is equal to 0, then skip to S47.

S46. **Since [DATE1]** (that is, in the last [DAYS] days), with how many of these [Response to S45] transmen whose HIV status you didn't know or were unsure about did you have sexual intercourse, WITHOUT using a condom?

__ __ __ Partners
998 Refuse to Answer

If S46 is greater than S45 then This number cannot be greater than &[TMUNK]. and skip to S46.

S47. **Since [DATE1]** (that is, in the last [DAYS] days), you have had sex with [TMKNW] **non-primary** TRANSMALE sexual partners where you KNEW their HIV status before you had sex with them. (Choose one)

0 [illegible]
1 [illegible]
8 [illegible]

If S47 is equal to 0 then skip to S45.
If TMKNW is equal to 0, then skip to instruction before S52.

S48. **Since [DATE1]** (that is, in the last [DAYS] days), how many of these [TMKNW] **non-primary** TRANSMALE sexual partners were HIV positive at the time you had sex with them?

__ __ __ Partners
998 Refuse to Answer

If S48 is greater than TMKNW then This number cannot be greater than &[TMKNW]. and skip to S48.
If S48 is equal to 0, then skip to S50.

S49. **Since [DATE1]** (that is, in the last [DAYS] days), with how many of the [Response to S48] HIV positive transmen did you have sexual intercourse, WITHOUT using a condom?

__ __ __ Partners
998 Refuse to Answer

If S49 is greater than S48 then This number cannot be greater than &[TMPOS]. and skip to S49.

S50. **Since [DATE1]** (that is, in the last [DAYS] days), you have had sex with [TMNEG] transmen who you believed were HIV negative before you had sex with them. (Choose one)

0 No, I wa[illegible]
1 Yes, I ha[illegible]
8 Refuse t[illegible]

If S50 is equal to 0 then skip to S48.
If TMNEG is equal to 0, then skip to instruction before S52.

S51. **Since [DATE1]** (that is, in the last [DAYS] days), with how many of the [TMNEG] HIV negative transmen did you have sexual intercourse, WITHOUT using a condom?

__ __ __ Partners
998 Refuse to Answer

If S51 is greater than TMNEG then This number cannot be greater than &[TMNEG] and skip to S51.
If S2 is equal to 0, then skip to instruction before T53.

S52. **Since [DATE1]** (that is, in the last [DAYS] days), have you had sex with any partner in exchange for any of the following?
(Check all that apply) (Check all that apply)

__ Food
__ Shelter
__ Transportation
__ Drugs or Alcohol
__ Money
__ None of these
__ Refuse to Answer

If S52F is equal to 1 and (S52A is equal to 1 or S52B is equal to 1 or S52C is equal to 1 or S52D is equal to 1 or S52E is equal to 1) then You cannot have had sex for "none of these" and some of these. and skip to S52.

CLIENT SATISFACTION QUESTIONNAIRE

If Q3a is not equal to 5, then skip to end of questionnaire.

Please help us improve REBOOT and future studies by answering a few questions about your time in REBOOT. We are interested in your honest opinions, whether they are positive or negative. Please answer all of the questions. We also welcome your comments and suggestions. Thank you very much; we really appreciate your help.

T53. How would you rate the quality of service you have received in REBOOT? (Choose one)

0 Excellent
1 Good
2 Fair
3 Poor

T54. Did you get the kind of service you wanted? (Choose one)

0 Yes, definitely
1 Yes, generally
2 No, not really
3 No, definitely not

T55. To what extent has our program met your needs? (Choose one)

0 Almost all of my needs have been met
1 Most of my needs have been met
2 Only a few of my needs have been met
3 None of my needs have been met

T56. If a friend were in need of similar help, would you recommend our program to him or her? (Choose one)

0 Yes, definitely
1 Yes, I think so
2 No, I don't think so
3 No, definitely not

T57. How satisfied are you with the amount of help you have received? (Choose one)

0 Very satisfied
1 Mostly satisfied
2 Indifferent or mildly dissatisfied
3 Quite dissatisfied

T58. Have the services you received helped you to deal more effectively with your problems? (Choose one)

0 Yes, they helped a great deal
1 Yes, they helped
2 No, they really didn't help
3 No, they seemed to make things worse

T59. In an overall, general sense, how satisfied are you with the service you have received? (Choose one)

0 Very satisfied
1 Mostly satisfied
2 Indifferent or mildly dissatisfied
3 Quite dissatisfied

T60. If you were to seek help again, would you come back to our program? (Choose one)

0 Yes, definitely
1 Yes, I think so
2 No, I don't think so
3 No, definitely not

T61. What additional comments or suggestions do you have for us?

__ __
__ __
__ __
__ __
__ __
__ __ __ __ __ __ __ __ __ __ __ __ __ __ __ __ __ __ __ __

Wrap Up

Thank you for taking this survey. Your responses are very important to us.
