# Supplementary material for: Behavioral intervention to reduce opioid overdose among high-risk persons with opioid use disorder: A pilot randomized controlled trial
Source: PLoS One. 2017 Oct 19;12(10):e0183354. doi: 10.1371/journal.pone.0183354 (PMC5648110; doi:10.1371/journal.pone.0183354)
Supplement: S2 File — (DOCX) [file pone.0183354.s002.docx]

san francisco department of public health

study protocol

| Study Title: | Repeated-dose Brief Intervention to Reduce Overdose and Risk Behaviors Among Naloxone Recipients |
| --- | --- |
|  |  |
| Sponsor: | San Francisco Department of Public Health  25 Van Ness Avenue, Suite 500  San Francisco, California 94102 |
|  |  |
| IND No.: | N/A |
|  |  |
| Indication: | Opioid overdose prevention |
|  |  |
|  |  |
| Principal Investigator: | Phillip O. Coffin, MD MIA  Director of Substance Use Research  San Francisco Department of Public Health  25 Van Ness Ave, Suite 500  San Francisco, CA 94102  Telephone: 415-437-6282 E‑mail: [Phillip.coffin@sfdph.org](mailto:Phillip.coffin@sfdph.org) |
|  |  |
| Regulatory Affairs: | This study has been submitted to the following IRB  UCSF Committee on Human Research  3333 California Street, Suite 315  University of California, San Francisco, CA 94118  Phone: 415-476-1814  Fax: 415-502-1347  Email: [chr@ucsf.edu](mailto:chr@ucsf.edu) |

| **TABLE OF CONTENTS** |  |
| --- | --- |
| PROTOCOL SYNOPSIS | 4 |
| 1. INTRODUCTION | 11 |
| 1.1. Background | 11 |
| 1.2. Overall Risk/Benefit Assessment | 11 |
| 1.3. Compliance | 12 |
| 2. OBJECTIVES | 13 |
| 3. STUDY DESIGN | 15 |
| 3.1. Study Treatment and Duration of Treatment | 15 |
| 3.2. Treatment Discontinuation Criteria | 15 |
| 4 SUBJECT POPULATION | 16 |
| 4.1. Number of Subjects and Subject Selection | 16 |
| 4.2. Inclusion Criteria | 16 |
| 4.3. Exclusion Criteria | 16 |
| 5. STUDY PROCEDURES | 17 |
| 5.1. Subject Enrollment and Treatment Assignment | 17 |
| 5.2. Pretreatment Assessments | 17 |
| 5.2.1. Screening Visit | 17 |
| 5.2.2. Enrollment Visit | 17 |
| 5.3. Monthly Treatment Visits (Month 4, 8, 12 and 16) | 18 |
| 5.4. Assessments | 18 |
| 5.4.1. Labs | 18 |
| 5.4.2. Computer Assisted Personal Interview (CAPI) | 18 |
| 5.4.3. Administrative Data | 19 |
| 5.4.4. Data Management | 19 |
| 6. ADVERSE EVENTS MANAGEMENT | 20 |
| 6.1. Definition of Adverse Events and Serious Adverse Events | 20 |
| 6.2. Management of SAEs and Other Study Risks | 20 |
| 6.3. Reporting of SAEs | 21 |
| 7. CHANGES TO STUDY PROTOCOL | 22 |
| 7.1. Reporting of IRB Actions to NIDA | 22 |
| 7.2. Report of Changes or Amendments to the Protocol | 22 |
| 7.3. Trial Stopping Rules | 22 |
| 7.4. Disclosure of Conflicts of Interest | 22 |
| 8 STATISTICAL CONSIDERATION | 23 |
| 8.1. Analysis Objectives | 23 |
| 8.1.1. Analysis Objectives | 23 |
| 8.2. Analysis Conventions | 23 |
| 8.3. Demographic Data and Baseline Characteristics | 23 |
| 8.4. Data Analysis | 23 |
| 8.4.1. Primary Analysis | 24 |
| 8.4.2. Exploratory Analysis | 24 |
| 8.5. Sample Size | 24 |
| 9 RESPONSIBILITIES | 25 |
| 9.1. Investigator Responsibilities | 25 |
| 9.1.1. Good Clinical Practice | 25 |
| 9.1.2. Institutional Review Board (IRB) Approval | 25 |
| 9.1.3. Informed Consent | 25 |
| 9.1.4 Confidentiality | 26 |
| 9.1.5. Study Files and Retention of Records | 26 |
| 9.1.6. Case Report Forms (CRFs) | 26 |
| 9.1.7. Protocol Compliance | 27 |
| 9.1.8. Study Discontinuation | 27 |
| 11 APPENDICES | 28 |
| Appendix 1. Study Procedures Table | 29 |
| 12 REFERENCES | 30 |

PROTOCOL SYNOPSIS

San Francisco Department of Public Health

25 Van Ness Ave, Suite 500

San Francisco CA 94102

| Study Title: | Repeated-dose Brief Intervention to Reduce Overdose and Risk Behaviors among Naloxone Recipients |
| --- | --- |
| IND Number: | N/A |
| Study Center(s) Planned: | 1 site in the United States |
| Number of Subjects Planned: | At least 60 subjects (60 who inject drugs, with up to 10 additional non-injecting subjects) |
| Target Population: | Opioid-dependent naloxone recipient adults who have overdosed |
| Duration of Treatment: | Subjects will be enrolled in trial for 16 months |
| Objectives: | The primary objectives of this study are as follows:   1. To determine the feasibility of a randomized trial with REBOOT. 2. To determine acceptability of REBOOT. 3. To evaluate the influence of egocentric social network characteristics on overdose events and naloxone use.   The exploratory objectives of this study are as follows:   1. Preliminary efficacy estimates of REBOOT in decreasing overdose events (non-fatal or death), decreasing frequency of modifiable overdose / drug-related HIV risk behaviors, and increasing days of abstinence from illicit opioids and in substance use treatment. 2. Treatment effects on modifiable overdose risk behaviors. To provide a preliminary estimate of the effect of REBOOT, compared to TAU, on overdose risk behaviors, as measured by frequency of using opioids with alcohol, cocaine, or benzodiazepines (the most common modifiable overdose risk behavior), we will use GEE Poisson models with robust standard errors to assess reductions in overdose risks and linear mixed models to assess effects on the RAB scale. 3. Treatment effects on HIV risk behaviors. To provide a preliminary estimate of the effect of REBOOT, compared to TAU, on HIV risk behaviors among high-risk opioid users who have received take-home naloxone, as measured by frequency of using opioids with alcohol, cocaine, or benzodiazepines (the most common modifiable overdose risk behavior); and the HIV Risk Assessment Battery (RAB) drug-related risk scale score over 16 months. We will use GEE Poisson models with robust standard errors to assess reductions in overdose risks and linear mixed models to assess effects on the RAB scale. 4. Treatment effects on discontinuation of opioid use. We will use GEE Poisson models with robust standard errors to assess evidence that the intervention increases days of abstinence from illicit opioids and days of drug treatment within each of five 4-month reporting periods. 5. Treatment effects on management of witnessed overdose*.* We will use GEE Poisson models with robust standard errors to assess evidence that the intervention increases the use of naloxone during overdose events witnessed by participants. 6. Prospective evaluation of naloxone recipients. Given the apparent effectiveness of naloxone alone in reducing overdose mortality, we will analyze all measures to generate descriptive data from the control arm. These include trajectories in self-reported drug use, baseline prevalence and incidence of HIV and HCV, longitudinal records on receipt of naloxone refills, and trends in utilization of EMS after naloxone administration (a potential cost savings of overdose prevention to be explored in future mathematical modeling efforts). Although sample size is small, results will contribute to the limited extant research on this topic and population of opioid users. 7. Validation of additional modifiable risk factors for overdose. We will also conduct exploratory analyses examining association of incident overdose with well-established, modifiable risk factors, considered as time-dependent covariates. Factors to be considered include: use of selected drugs with opioids; periods of abstinence (self-imposed or structural);^4, 5^ increases in opioid dose,[^6^](#_ENREF_165) in particular after a period of abstinence ≥2 days; and use of opioids from a new source[^3^](#_ENREF_54) or in a new location.[^7^](#_ENREF_162) While these relationships have been assessed in cross-sectional studies, our prospective data will allow us to assess the effects of both recent exposures as well as longer-term exposure patterns. 8. Treatment effects and depression. Treatment effects and depression will be addressed by examining both modification and mediation of the treatment effect on overdose events by depression, as measured by the Center for Epidemiologic Studies-Depression Scale (CES-D). Effect modification will be examined by comparing treatment effects according to baseline depression score. Mediation will be assessed by examining both steps in the indirect pathway (treatment🡪depression, and depression🡪OD/death, controlling for treatment and potential confounders of the mediator/outcome relationship). |
| Study Design: | We will enroll at least 60 opioid-dependent naloxone recipients with a prior opioid overdose who will be randomized to receive repeated doses of a 45-minute IMB (Informational-Motivational-Behavior) –based counseling session in overdose prevention versus treatment as usual (TAU, defined as information and referrals), and followed over 16 months with 4 counseling sessions at months 0 (enrollment), 4, 8, and 12 for the intervention arm. All participants will complete an opioid urine screen at all visits, a Computer Assisted Personal Interview (CAPI) at enrollment and months 4, 8, 12, and 16, and rapid HIV test (or CD4 & viral load test for positive participants) and rapid HCV test at screening and month 16. |
| Inclusion Criteria: | (1) Age 18-65 years;  (2) Current opioid dependence by SCID;  (3) Urine positive for opioids during screening, excluding prescribed agonist maintenance therapy;  (4) History of prior opioid overdose;  (5) Previously received take-home naloxone;  (6) No serious illnesses likely to progress clinically during trial;  (7) Able and willing to provide informed consent, provide locator information, communicate in English, adhere to visit schedule. |
| Exclusion Criteria: | (1) Suicidal ideation by SCID;  (2) Currently participating in another interventional research study that could possible impact the study’s outcomes of interest;  (3) Any condition that, in the Principal Investigator’s judgment, interferes with safe study participation or adherence to study procedures. |
| Study Procedures/ Frequency: | All subjects will complete the following study visits: screening, enrollment, study visits every 4 months for a total of 16 months.  **Screen visit:** informed consent will be obtained at the beginning of the screening visit. After consent is obtained and eligibility is determined, a locator form is completed and HIPAA form is signed for substance abuse record retrieval and review of medical/criminal records. Screening visit will also include a clinical evaluation (Structured Clinical Interview for DSM-IV [SCID] for opioid dependence and suicidal ideation), blood-borne virus testing (rapid HIV/hepatitis C serology [if no confirmed serostatus] +/- CD4+ t cells & HIV viral load), and a urine toxicology test with expanded opioids.  **Enrollment (month 0):** At the enrollment visit, participants will be randomized to receive the behavioral intervention (n=40) or TAU (n=20). **Randomization** will be 2:1 using permuted blocks of randomly selected sizes 3 and 6. Participants will provide a urine toxicology text, and will undergo the baseline CAPI followed by initial counseling visit. The next visit will be scheduled and participants will be compensated for their time.  **Every 4-month follow up visits:** participant will be seen every 4 months for CAPI, followed by counseling for the intervention arm; the control group will be offered referrals to local services. At months 4, 8 and 12 participants will provide a urine toxicology text, and will undergo the baseline CAPI followed by counseling for participants in the treatment arm.  At month 16, participants will complete activities listed for the monthly visits, in addition to completing the SCID and a blood-borne virus testing (rapid HIV/hepatitis C serology [if no confirmed serostatus] +/- CD4+ t cells & HIV viral load). Participants in the treatment arm will not receive counseling at month 16.  The CAPI, which is administered at enrollment and months 4, 8, 12, and 16 includes demographic information (enrollment only), drug use history (enrollment only), extensive opiate overdose questions, drug use and drug-related risk behaviors, partner-by-partner data on injection/substance use/HCV status, quality of life scale (EQ5-D), Center for Epidemiologic Studies Depression Rating Scale, and Severity of Dependence Scale and participant satisfaction with treatment. |
| Treatment Arm: | REBOOT participants will receive repeated doses of a 45-minute IMB (Informational-Motivational-Behavior) –based counseling session in overdose prevention. |
| Reference Arm: | Participants in the reference arm will receive treatment as usual (TAU), which consists of referrals to harm reduction and other substance use services. |
| Statistical Methods: | Specific Aim 1: To determine feasibility of a randomized trial with REBOOT, we will calculate screening and visit completion rates from the study database, with exact 95% confidence intervals (Cis), overall and by arm. Between-group differences will be assessed using Fisher’s exact and Wilcoxon ranksum tests. We will calculate Kaplan-Meier curves for time to dropout, by group, and test for differences using the log-rank test.  Specific Aim 2: To determine acceptability of REBOOT, we will calculate counseling completion rates from the study database by visit, and tabulate the proportions of active arm participants attending 0-4 counseling sessions. Via CAPI, we will inquire about participant satisfaction with the intervention and belief that it affected their drug use behaviors; responses will be presented as means, medians, or proportions, as appropriate, with 95% Cis.  Specific Aim 3: To evaluate the influence of egocentric social network characteristics on overdose events and naloxone use, we will use GEE Poisson models with robust standard errors to evaluate the association between network size, evaluated at baseline and each return visit, and numbers of experienced and witnessed overdose events in the same period; zero-inflated models will be used if needed. Results from this analysis will inform study design of a full efficacy trial and determine if randomization stratified by network size or targeted sampling strategies to ensure diversity in network size are needed. In addition, we will explore the influence of homophily and assortativity on experienced and witnessed overdose events using similar methods. Newman’s method will be used to calculate assortativity coefficients, a measure of the degree of demographic and risk behavior similarity within participants’ egocentric networks.  **Secondary analyses**: Exploratory analyses include preliminary efficacy estimates of REBOOT in decreasing overdose events (non-fatal or death), decreasing frequency of modifiable overdose / drug-related HIV risk behaviors, and increasing days of abstinence from illicit opioids and in substance abuse treatment.  Treatment effects on frequency of overdose events***.*** To determine if REBOOT, compared to TAU, reduces the risk of overdose among high-risk opioid users who have received take-home naloxone, as measured by the number of overdose events over 16 months. Overdose events will be determined by CAPI every 4 months via time-line follow-back of questions to determine the approximate date of non-fatal overdose that have proven reliable in multiple settings,^1-3^ with fatal episodes identified as above. We expect that repeated overdose events may be reasonably common in a subset of higher risk participants. To take advantage of this information, we will estimate treatment effects using Poisson models for the number of OD events in each reporting period, with robust standard errors to account for within-subject correlation and over-dispersion. |

This study will be conducted in accordance with the guidelines of Good Clinical Practices (GCPs) including archiving of essential documents.

# INTRODUCTION

## Background

The United States is amidst an opioid crisis, with increasing rates of opioid overdose mortality. While naloxone distribution has demonstrated benefits in reducing opioid overdose mortality, more is needed, in particularly interventions to reduce the risk of overdose occurring in the first place. We developed a brief motivational interviewing intervention, based on the IMB model of behavior change, to address opioid overdose, as well as related HIV/HCV risk behaviors, among persons who already have access to naloxone but remain at high risk for opioid overdose.

## Overall Risk/Benefit Assessment

**Primary Patient Risks:**

We do not anticipate any moderate, severe, or life-threatening adverse events as a result of study participation. If a participant should report or display physical problems (such as hyperventilation, ongoing suicidal ideation, serious emotional distress, or similar problems) study staff will suggest that a call be placed to an appropriate referral agency or crisis hotline. The staff member may also call 911 if deemed necessary. Referral lists for mental health and substance abuse services and crisis hotlines will be available for reference at all times and provided to participants upon request. Study staff will be trained in how to identify, manage, and respond to these events. If needed, study staff will arrange for counselors or medical personnel to provide assistance to those participants that experience any serious effects during the course of the study.

Potential risks from study participation will be explained through the informed consent process. The study will not dispense naloxone to participants. Prior to enrollment, participants will have received naloxone (all community distributed naloxone in San Francisco currently comes from the Drug Overdose Prevention and Education [DOPE] Project); prior receipt of take-home naloxone is one of the study inclusion criteria. Therefore, this behavioral study does not pose any additional risks of pharmacologic agents nor does it raise ethical concerns about withholding this potentially life-saving agent. However, participants are informed of potential risks involved in study participation. Risks include those associated with blood draws, such as bruising around the needle site, the risk of infection at the needle site, and occasional equipment failure in the vacuum tubes. Other potential risks to participants include: unauthorized disclosure of confidential information; discomfort or embarrassment related to specimen collection or questionnaires dealing with personal habits and lifestyle, including drug or alcohol use; possible unwanted encounters with friends or associates in the research setting; and continued drug use, with its attendant risks, including risk of overdose.

**Alternative treatments and procedures in place to mitigate patient risks:** Participants have the alternative of not participating in the study; this decision will in no way influence their treatment by study staff. We will make it clear to all potential participants that they have the alternative of not participating in the study, emphasizing that their decision will in no way influence their treatment by the University, Health Department, or the DOPE Project. We will also offer referrals to inpatient and outpatient substance use treatment facilities and services in San Francisco.

**Benefits:** Potential benefits to participants include: possible cessation or reduction in opioid use and reductions in HIV risk behaviors. Potential benefits to society include determining whether an IMB (Informational-Motivational-Behavior) –based intervention is feasible and acceptable among opioid-dependent, naloxone recipients.

## Compliance

This study will be conducted in compliance with this protocol, Good Clinical Practice (GCP), and all applicable regulatory requirements. There is no DSMB for this study.

# OBJECTIVES

The primary objectives of this study are:

- To determine the feasibility of a randomized trial with REBOOT.
- To determine acceptability of REBOOT.
- To evaluate the influence of egocentric social network characteristics on overdose events and naloxone use.

Exploratory objectives of this study are:

- To determine the preliminary efficacy estimates of REBOOT in decreasing overdose events (non-fatal or death), decreasing frequency of modifiable overdose / drug-related HIV risk behaviors, and increasing days of abstinence from illicit opioids and in substance use treatment.
- To provide a preliminary estimate of the effect of REBOOT, compared to TAU, on overdose risk behaviors, as measured by frequency of using opioids with alcohol, cocaine, or benzodiazepines (the most common modifiable overdose risk behavior), we will use GEE Poisson models with robust standard errors to assess reductions in overdose risks and linear mixed models to assess effects on the RAB scale.
- To provide a preliminary estimate of the effect of REBOOT, compared to TAU, on HIV risk behaviors among high-risk opioid users who have received take-home naloxone, as measured by frequency of using opioids with alcohol, cocaine, or benzodiazepines (the most common modifiable overdose risk behavior); and the HIV Risk Assessment Battery (RAB) drug-related risk scale score over 16 months. We will use GEE Poisson models with robust standard errors to assess reductions in overdose risks and linear mixed models to assess effects on the RAB scale.
- We will use GEE Poisson models with robust standard errors to assess evidence that the intervention increases days of abstinence from illicit opioids and days of drug treatment within each of five 4-month reporting periods.
- We will use GEE Poisson models with robust standard errors to assess evidence that the intervention increases the use of naloxone during overdose events witnessed by participants.
- Given the apparent effectiveness of naloxone alone in reducing overdose mortality, we will analyze all measures to generate descriptive data from the control arm. These include trajectories in self-reported drug use, baseline prevalence and incidence of HIV and HCV, longitudinal records on receipt of naloxone refills, and trends in utilization of EMS after naloxone administration (a potential cost savings of overdose prevention to be explored in future mathematical modeling efforts). Although sample size is small, results will contribute to the limited extant research on this topic and population of opioid users.
- We will also conduct exploratory analyses examining association of incident overdose with well-established, modifiable risk factors, considered as time-dependent covariates. Factors to be considered include: use of selected drugs with opioids; periods of abstinence (self-imposed or structural);^4, 5^ increases in opioid dose,[^6^](#_ENREF_165) in particular after a period of abstinence ≥2 days; and use of opioids from a new source[^3^](#_ENREF_54) or in a new location.[^7^](#_ENREF_162) While these relationships have been assessed in cross-sectional studies, our prospective data will allow us to assess the effects of both recent exposures as well as longer-term exposure patterns.
- Treatment effects and depression will be addressed by examining both modification and mediation of the treatment effect on overdose events by depression, as measured by the Center for Epidemiologic Studies-Depression Scale (CES-D). Effect modification will be examined by comparing treatment effects according to baseline depression score. Mediation will be assessed by examining both steps in the indirect pathway (treatment🡪depression, and depression🡪OD/death, controlling for treatment and potential confounders of the mediator/outcome relationship).

# STUDY DESIGN

## Study Treatment and Duration of Treatment

This is two-arm randomized behavioral study to evaluate the feasibility, acceptability, and effectiveness of IMB counseling versus TAU in opioid-dependent naloxone recipient persons.

Sixty subjects will be enrolled in one of the following two study arms.

**Group 1**: IMB Counseling (REBOOT)

- - - Treatment arm participants will receive a single brief session of IMB counseling on overdose prevention at enrollment and months 4, 8, and 12.

**Group 2**: TAU

- - - Reference arm participants will receive TAU including referrals available to all participants.

## Treatment Discontinuation Criteria

There are no formal trial-stopping rules for this study. No formal interim efficacy analysis will be conducted.

If it becomes clear that the trial puts undue safety risk on study participants, outcomes are poor, or the trial will not achieve its enrollment goals, consideration will be given to stopping the trial, after consultation with the IRB and NIDA program officer (PO).

The overall safety risk to study participants will be determined through regular monitoring procedures. Safety issues will be evaluated as they arise; participants are given the pager number of the clinician on call which they can page in the event of an emergency or safety risk. Study clinicians will consult with Dr. Coffin, the medical director and Principal Investigator, on these safety issues on a case-by-case basis as they are reported by the participant. Non-urgent clinical issues that arise during the course of the study are discussed by the team’s research clinicians at the next weekly meeting with the PI Dr. Coffin. During weekly meetings, study clinicians will review all the safety issues and incident adverse events (including lab abnormalities) for the study overall, by system category, and by possible relationship to the behavioral intervention. The PI will alert the NIDA PO immediately if at any point the team observes an unexpected frequency of serious Aes possibly related to the intervention.

# SUBJECT POPULATION

## Number of Subjects and Subject Selection

At least 60 subjects will be enrolled in this study, potentially up to 70, to ensure at least 60 people who inject drugs are included.

## Inclusion Criteria

Subjects must meet *all* of the following inclusion criteria to be eligible for participation in this study.

1. Age 18-65 years;
2. Current opioid dependence by SCID;
3. Urine positive for opioids during screening, excluding prescribed agonist maintenance therapy;

(4) History of prior opioid overdose;

(5) Previously received take-home naloxone;

(6) No serious illnesses likely to progress clinically during trial;

(7) Able and willing to provide informed consent, provide locator information, communicate in English, adhere to visit schedule.

## Exclusion Criteria

Subjects who meet *any* of the following exclusion criteria are not to be enrolled in this study.

(1) Suicidal ideation by SCID;

(2) Currently participating in another interventional research study that could possible impact the study’s outcomes of interest;

- 1. Any condition that, in the Principal Investigator’s judgment, interferes with safe study participation or adherence to study procedures.

# STUDY PROCEDURES

The study procedures to be conducted for each subject enrolled in the study are presented in tabular form in Appendix 1 and described in the text that follows. Additional information is provided in the study procedures manual.

The investigator will document any deviation from protocol procedures and notify appropriate regulatory authorities (e.g., IRB and/or NIH).

## Subject Enrollment and Treatment Assignment

In-person visits will occur at screening, enrollment, and every four months for 16 months.

## Pretreatment Assessments

### Screening Visit

Subjects will be screened within 30 days of enrollment to determine eligibility for participation in the study. The screening window can be extended to 42 days in exceptional circumstances. Subjects meeting all of the inclusion criteria and none of the exclusion criteria will return to the clinic within 30 days after screening for enrollment into the study.

Informed consent will be obtained at the beginning of the screening visit. After consent is obtained and eligibility is determined, a locator form is completed and HIPAA form is signed for substance abuse record retrieval and review of medical/criminal records. Screening visit will also include a clinical evaluation (Structured Clinical Interview for DSM-IV [SCID] for opioid dependence and suicidal ideation), blood-borne virus testing (rapid HIV/hepatitis C serology [if no confirmed serostatus] +/- CD4+ t cells & HIV viral load), and a urine toxicology test with expanded opioids.

Subjects will be compensated $10 for the screening visit.

### Enrollment Visit

Enrollment tests and procedures will be completed prior to enrollment and receipt of the first counseling session.

On the enrollment visit, participants will be randomized to receive the behavioral intervention (n=40) or TAU (n=20). **Randomization** will be 2:1 using permuted blocks of randomly selected sizes 3 and 6.

Participants will provide a urine toxicology test, and will undergo the baseline CAPI followed by initial counseling for the treatment group and TAU for the reference group. The next visit will be scheduled and participants will be compensated for their time.

Subjects will be compensated $30 for the enrollment visit.

## Monthly Treatment Visits (Month 4, 8, 12 and 16)

Participant will be seen every 4 months.

At months 4, 8 and 12 participants will provide a urine toxicology test, complete the CAPI, and receive counseling for the treatment group and TAU for the reference group.

At month 16, participants will complete activities lists for the monthly visits in addition to completing the SCID and a blood-borne virus testing (rapid HIV/hepatitis C serology [if no confirmed serostatus] +/- CD4+ t cells & HIV viral load). REBOOT Participants will not receive counseling at month 16.

Subjects will be compensated as follows: $35 for month 4 visit, $45 for month 8, $55 for month 12, and $65 for month 16.

## Assessments

## Labs

Urine toxicology test (drugs of abuse screen/expanded opioids, including fentanyl) will be done each visit.

### 5.4.2. Computer Assisted Personal Interview (CAPI)

All participants will receive a CAPI at enrollment and months 4, 8, 12, and 16. The CAPI will **always** be conducted by a staffperson blinded to participant’s study arm and prior to administration of the REBOOT intervention or control arm procedures.

The CAPI includes demographic information (enrollment only), drug use history (enrollment only), extensive opiate overdose questions, drug use and drug-related risk behaviors, partner-by-partner data on injection/substance use/HCV status, quality of life scale (EQ5-D), Center for Epidemiologic Studies Depression Rating Scale, and Severity of Dependence Scale and participant satisfaction with treatment.

Primary and secondary outcome variables will be obtained from standardized scales assessed using CAPI to minimize underreporting of risk activities and enhance standardization.^4^ We will utilize the same overdose measures as in the pilot, which were developed in collaboration with other investigators (Drs Caleb Banta-Green, Traci Green, Amy Bohnert, and Alex Walley) to maximize comparability among overdose studies.

- **Quality of Life Surveys:** The quality of life scale included in this study is the EQ5-D. It will be completed by participants at enrollment and months 4, 8, 12 and 16 regardless of study arm. The Quality of Life Survey will be included in the CAPI.
- **Depression Rating Scale** The depression rating scale included in this study is the Center for Epidemiologic Studies Depression Rating Scale. It will be completed by participants at enrollment and months 4, 8, 12 and 16 regardless of study arm. The Depression Rating Scale will be included in the CAPI.
- **Severity of Dependence Scale:** The Severity of Dependence Scale will be completed by participants at enrollment and months 4, 8, 12 and 16 regardless of study arm. The Severity of Dependence Scale will be included in the CAPI.

**5.4.3. Administrative data**

Date and cause of death will be ascertained from **Vital Records**, as well as contacts listed on locator forms for those participants lost-to-follow-up.

**SUDTx data**, from 4 months pre-enrollment to study completion, will be obtained with appropriate HIPAA/CFR-42 release.

**5.4.4. Data Management**

Data will be maintained in MS Access (tracking and administrative data), QDS warehouse manager (survey data). All data will be backed up nightly at the study site based on San Francisco Department of Public Health protocols.

# ADVERSE EVENTS MANAGEMENT

## 6.1 Definition of Adverse Events and Serious Adverse Events

For this study, we will record and report only adverse events (Aes) directly related to study participation. We will not report opiate overdose events as adverse events.

In the event of study-related Aes, we will use the DAIDS Table for Grading the Severity of Adult and Pediatric Adverse Events version 1.0, December 2004 (Clarification dated August 2009) available at: <http://rsc.tech-res.com/safetyandpharmacovigilance/>

We will record and report all study-related Aes in accordance with these NIH guidelines. Safety monitoring will include the assessment, follow-up, and reporting of clinical Aes and serious Aes (SAEs), and abnormal laboratory results. Each AE will be classified by the study clinician as serious or non-serious, and appropriate reporting procedures will be followed.

A ***serious AE*** is any untoward medical occurrence related to study activities:

- results in death,
- is life-threatening (defined as an event in which the patient was at risk of death at the time of the event; it does not refer to an event which hypothetically might have caused death if it were more severe),
- requires inpatient hospitalization or causes prolongation of existing hospitalization,
- results in persistent or significant disability/incapacity,
- is a congenital anomaly/birth defect,
- is an important medical event (defined as a medical event(s) that may not be immediately life-threatening or result in death or hospitalization but, based upon appropriate medical and scientific judgment, may jeopardize the patient or may require intervention (e.g., medical, surgical) to prevent one of the other serious outcomes listed in the definition above.

An unexpected event is one that is not described with respect to nature, severity, or frequency in the current protocol.

Adverse events classified as “serious” require expeditious handling and reporting to comply with regulatory requirements.

**6.2 Management of SAEs and other study risks:** Participants requiring acute medical care will be treated on-site by a clinician, and, once stabilized, taken to the appropriate hospital for care (most likely, depending on availability, San Francisco General Hospital which is five minutes by ambulance). All participants will be provided with a 24-hour phone number through which the study clinician may be contacted to answer questions or to provide direction in case of emergency**.**

**6.3 Reporting of SAEs**: All Aes that are serious and meet the reporting criteria will be reported to the UCSF IRB in writing, within 10 working days.

SAE reporting will include a narrative which will provide details of relevant screening measures, medical history and physical, treatment compliance, participant reports of SAEs, and any other required information. The completed SAE form will contain: subject’s ID#, gender, age, the title and date of the SAE, and narrative explanation. The SAE form tracks how the research staff was notified of the event, dates of consent, randomization, study screening for inclusion/exclusion, study treatment received, other relevant clinical information, dates and circumstances of the hospitalization/death, whether alcohol or drugs were known to be involved, and participant status at last clinical or research contact. The investigators will state whether the event was expected and assess its relatedness to the study intervention. A summary report of all Aes (including SAEs) will be submitted to the IRB and to NIDA in the annual progress report.

Collection of complete information concerning SAEs is extremely important. Follow-up information which becomes available as the SAE evolves, as well as supporting documentation (e.g., hospital discharge summaries and autopsy reports), should be collected subsequently, if not available at the time of the initial report, and immediately sent using the same procedure as the initial SAE report.

###### An expedited reporting of SAE will adhere to the following guidelines:

1. Apply regardless of the investigator’s assessment of the relatedness of the SAE to the intervention under study.
2. Apply equally to trials requiring an IND and those not requiring an IND.
3. Apply to any SAE that occur during the post-treatment observation period defined by the protocol.
4. Apply to suicidal or homicidal behavior that causes an SAE in the subject or someone else (*e.g.* hospitalization or death).
   1. **CHANGES TO STUDY PROTOCOL**

**7.1 Reporting of IRB actions to NIDA:** Through the study project officer, NIDA will be informed of IRB actions regarding the study. When significant revisions to the protocol are planned, IRB communications will be forwarded to the NIDA PO.

**7.2 Report of changes or amendments to the protocol:** All changes and amendments to the study protocol will require IRB approval prior to their implementation. When appropriate, the NIDA PO will be informed of changes or amendments to the study protocol.

**7.3 Trial stopping rules:** There are no formal trial-stopping rules for this study. No formal interim efficacy analysis will be conducted.

If it becomes clear that the trial puts undue safety risk on study participants, outcomes are poor, or the trial will not achieve its enrollment goals, consideration will be given to stopping the trial, after consultation with the IRB and NIDA PO.

The overall safety risk to study participants will be determined through regular monitoring procedures. Safety issues will be evaluated as they arise; participants are given the pager number of the clinician on call which they can page in the event of an emergency or safety risk. Study clinicians will consult with Dr. Coffin, the medical director and Principal Investigator, on these safety issues on a case-by-case basis as they are reported by the participant. Non-urgent clinical issues that arise during the course of the study are discussed by the team’s research clinicians at the next weekly meeting with the PI Dr. Coffin. During weekly meetings, study clinicians will review all the safety issues and incident adverse events (including lab abnormalities) for the study overall, by system category, and by possible relationship to the behavioral intervention. The PI will alert the DMSB and the NIDA P.O. immediately if at any point the team observes an unexpected frequency of serious Aes possibly related to the intervention.

**7.4 Disclosure of conflicts of interest:** Staff are required to disclose any financial conflicts of interest with the study. Signed documentation of conflicts of interest by study investigators (or lack thereof) will be provided to the IRB as required by the initial and continuing review process.

# STATISTICAL CONSIDERATIONS

## Analysis Objectives

### Analysis Objectives

The primary objectives of this study are:

- To determine the feasibility of a randomized trial with REBOOT.
- To determine acceptability of REBOOT.
- To evaluate the influence of egocentric social network characteristics on overdose events and naloxone use.

The exploratory objectives of this study are:

- To determine the preliminary efficacy estimates of REBOOT in decreasing overdose events (non-fatal or death), decreasing frequency of modifiable overdose / drug-related HIV risk behaviors, and increasing days of abstinence from illicit opioids and in substance use treatment.

## Analysis Conventions

All individual subject data will be listed as measured. All statistical summaries and analyses will be performed using STATA^®^ software.

## Demographic Data and Baseline Characteristics

Demographic and baseline characteristics will be summarized using standard descriptive methods by treatment group and overall.

Demographic data will include sex, self-identified race/ethnicity, and age.

## Data Analysis

### Primary Analysis

Specific Aim 1: To determine feasibility of a randomized trial with REBOOT, we will calculate screening and visit completion rates from the study database, with exact 95% confidence intervals (Cis), overall and by arm. Between-group differences will be assessed using Fisher’s exact and Wilcoxon ranksum tests. We will calculate Kaplan-Meier curves for time to dropout, by group, and test for differences using the log-rank test.

Specific Aim 2: To determine acceptability of REBOOT, we will calculate counseling completion rates from the study database by visit, and tabulate the proportions of active arm participants attending 0-4 counseling sessions. Via CAPI, we will inquire about participant satisfaction with the intervention and belief that it affected their drug use behaviors; responses will be presented as means, medians, or proportions, as appropriate, with 95% Cis.

Specific Aim 3: To evaluate the influence of egocentric social network characteristics on overdose events and naloxone use, we will use GEE Poisson models with robust standard errors to evaluate the association between network size, evaluated at baseline and each return visit, and numbers of experienced and witnessed overdose events in the same period; zero-inflated models will be used if needed. Results from this analysis will inform study design of a full efficacy trial and determine if randomization stratified by network size or targeted sampling strategies to ensure diversity in network size are needed. In addition, we will explore the influence of homophily and assortativity on experienced and witnessed overdose events using similar methods. Newman’s method will be used to calculate assortativity coefficients, a measure of the degree of demographic and risk behavior similarity within participants’ egocentric networks.

### Exploratory Analyses

Exploratory analyses include preliminary efficacy estimates of REBOOT in decreasing overdose events (non-fatal or death), decreasing frequency of modifiable overdose / drug-related HIV risk behaviors, and increasing days of abstinence from illicit opioids and in substance abuse treatment.

## Sample Size

**Sample size justification:** The sample will provide typical precision for a pilot study of feasibility and acceptability outcomes. Specifically, we will be able to estimate the mean of continuous outcomes within margins of sampling error (MSEs; half-widths of 95% confidence intervals) of 0.32 standard deviations (SDs) within the active arm, and 0.26 SDs overall; proportions will be estimated within MSEs of 9-16 percentage points in the active arm and 8-13 percentage points overall. For Aim 3 and the exploratory analyses of treatment efficacy, precision and power will be low, as is also typical for a pilot study. In designing the full-scale trial, these exploratory results will be cautiously interpreted in the light of confidence intervals, plausibility, and prior results from the literature.

# RESPONSIBILITIES

## Investigator Responsibilities

### Good Clinical Practice

The investigator will ensure that this study is conducted in accordance with the principles of the Declaration of Helsinki (as amended in Edinburgh, Tokyo, Venice, Hong Kong, and South Africa), International Conference on Harmonisation (ICH) guidelines, or with the laws and regulations of the country in which the research is conducted, whichever affords the greater protection to the study subject.

The investigator and all applicable sub-investigators will comply with 21 CFR, Part 54, 1998, providing documentation of their conflicts of interest. This documentation must be provided prior to the investigator’s (and any sub-investigator’s) participation in the study. Study completion is defined as the date when the last subject completes the protocol-defined activities.

### Institutional Review Board (IRB) Approval

The investigator (or sponsor as appropriate according to local regulations) will submit this protocol, informed consent form, and any accompanying material to be provided to the subject (such as advertisements, subject information sheets, or descriptions of the study used to obtain informed consent) to an IRB. The investigator will not begin any study subject activities until approval from the IRB has been documented and provided as a letter to the investigator.

Before implementation, the investigator will submit to and receive documented approval from the IRB any modifications made to the protocol or any accompanying material to be provided to the subject after initial IRB approval, with the exception of those necessary to reduce immediate risk to study subjects.

### Informed Consent

Study staff are responsible for obtaining written informed consent from each individual participating in this study after adequate explanation of the aims, methods, objectives, and potential hazards of the study and before undertaking any study-related procedures. The investigator will use the most current IRB approved consent form for documenting written informed consent. Each informed consent will be appropriately signed and dated by the subject or the subject’s legally authorized representative and the person conducting the consent discussion, and also by an impartial witness if required by IRB or by local requirements. The consent form will inform subjects about sample retention and use of retained samples.

### Confidentiality

The investigator will assure that subjects’ anonymity will be strictly maintained and that their identities are protected from unauthorized parties. Only subject initials, date of birth, and a unique identifier (as allowed by local law) will be recorded on any form or biological sample submitted to the Sponsor, IRB, or laboratory. Laboratory specimens will be labeled in such a way as to protect subject identity while allowing the results to be recorded to the proper subject. Refer to specific laboratory instructions. NOTE: If given signed permission by the subject the investigators keeps a locator form showing names, date of birth and addresses for all subjects screened and for all subjects enrolled in the trial. Subject data will be processed in accordance with all applicable regulations.

### Study Files and Retention of Records

The investigator will maintain adequate and accurate records to enable the conduct of the study to be fully documented and the study data to be subsequently verified. These documents should be classified into at least the following two categories: (1) investigator’s study file, and (2) subject clinical source documents.

The investigator’s study file will contain the protocol/amendments, CRF and query forms, IRB and governmental approval with correspondence, informed consent, drug records, staff curriculum vitae and authorization forms, and other appropriate documents and correspondence.

The required source data should include sequential notes containing at least the following information for each subject:

- Subject identification (name, date of birth, gender);
- Documentation that subject meets eligibility criteria;
- Documentation of the reason(s) a consented subject is not enrolled
- Participation in study (including study number);
- Study discussed and date of informed consent;
- Dates of all visits;
- Documentation that protocol specific procedures were performed;
- Results of efficacy parameters, as required by the protocol;
- Participant study start and end date, including treatment arm;
- Record of all adverse events and other safety parameters (start and end date, and including causality and severity);
- Date of study completion and reason for early discontinuation, if it occurs.

### Case Report Forms (CRFs)

For each subject consented, a CRF will be completed by an authorized study staff member whose training for this function is documented according to study procedures. CRF should be completed on the day of the subject visit to enable the sponsor to perform central monitoring of safety data. Prior to database lock (or any interim time points as described in the clinical data management plan), study staff will use his/her log in credentials to confirm that the forms have been reviewed, and that the entries accurately reflect the information in the source documents. The CRF capture the data required per the protocol schedule of events and procedures.

### Protocol Compliance

The investigator is responsible for ensuring the study is conducted in accordance with the procedures and evaluations described in this protocol.

### Study Discontinuation

The investigator reserves the right to terminate the study at any time. Should this be necessary, the investigator will arrange discontinuation procedures and notify the appropriate regulatory authority(ies) and IRB.

**11. APPENDICES**

Appendix 1. Study Procedures Table

Appendix 1. Study Procedures Table

Screening, Enrollment, Monthly Visits

| Procedure | Screening visits | Enrollment | Visit identified by study month | | | |
| --- | --- | --- | --- | --- | --- | --- |
|  |  |  | 4 | 8 | 12 | 16 |
| Informed consent | X |  |  |  |  |  |
| Brief history, SCID | X |  |  |  |  | X |
| Opioid urine test | X | X | X | X | X | X |
| Rapid HIV test* or CD4 & HIV VL and HIV counseling | X |  |  |  |  | X |
| Raid HCV test and HCV counseling | X |  |  |  |  | X |
| Randomization |  | X |  |  |  |  |
| Skills Building & Counseling (treatment arm) |  | X | X | X | X |  |
| CAPI |  | X | X | X | X | X |
| *In the event of a reactive test, confirmatory HIV testing will be performed | | | | | | |

**12. REFERENCES**

1. Coffin PO, Tracy M, Bucciarelli A, Ompad D, Vlahov D, Galea S. Identifying injection drug users at risk of nonfatal overdose. *Acad Emerg Med.* 2007;14(7):616-623.

2. Darke S, Ross J, Hall W. Overdose among heroin users in Sydney, Australia: I. Prevalence and correlates of non-fatal overdose. *Addiction.* 1996;91(3):405-411.

3. Ochoa KC, Hahn JA, Seal KH, Moss AR. Overdosing among young injection drug users in San Francisco. *Addict Behav.* 2001;26(3):453-460.

4. Fischman MW, Foltin RW. Utility of subjective-effects measurements in assessing abuse liability of drugs in humans. *Br J Addict.* 1991;86(12):1563-1570.
